# Differential expression of the *HvCslF6* gene late in grain development may explain quantitative differences in (1,3;1,4)-β-glucan concentration in barley

**DOI:** 10.1007/s11032-015-0208-6

**Published:** 2015-01-20

**Authors:** Sie Chuong Wong, Neil J. Shirley, Alan Little, Kelvin H. P. Khoo, Julian Schwerdt, Geoffrey B. Fincher, Rachel A. Burton, Diane E. Mather

**Affiliations:** 1 ARC Centre of Excellence in Plant Cell Walls, School of Agriculture, Food and Wine, Waite Research Institute, The University of Adelaide, Waite Campus, Glen Osmond, SA 5064 Australia; 2 School of Agriculture, Food and Wine, Waite Research Institute, The University of Adelaide, Waite Campus, Glen Osmond, SA 5064 Australia; 3 Present Address: Faculty of Agriculture and Food Sciences, Universiti Putra Malaysia Bintulu Campus, 97000 Bintulu, Malaysia

**Keywords:** Barley breeding, (1,3;1,4)-β-Glucan concentration, Molecular markers, Grain quality, QTL, Transcript profiles

## Abstract

The cellulose synthase-like gene *HvCslF6*, which is essential for (1,3;1,4)-β-glucan biosynthesis in barley, collocates with quantitative trait loci (QTL) for grain (1,3;1,4)-β-glucan concentration in several populations, including CDC Bold × TR251. Here, an alanine-to-threonine substitution (caused by the only non-synonymous difference between the CDC Bold and TR251 *HvCslF6* alleles) was mapped to a position within HvCSLF6 that seems unlikely to affect enzyme stability or function. Consistent with this, transient expression of full-length *HvCslF6* cDNAs from CDC Bold and TR251 in *Nicotiana* *benthamiana* led to accumulation of similar amounts of (1,3;1,4)-β-glucan accumulation. Monitoring of *HvCslF6* transcripts throughout grain development revealed a significant difference late in grain development (more than 30 days after pollination), with TR251 [the parent with higher grain (1,3;1,4)-β-glucan] exhibiting higher transcript levels than CDC Bold. A similar difference was observed between Beka and Logan, the parents of another population in which a QTL had been mapped in the *HvCslF6* region. Sequencing of a putative promoter region of *HvCslF6* revealed numerous polymorphisms between CDC Bold and TR251, but none between Beka and Logan. While the results of this work indicate that naturally occurring quantitative differences in (1,3;1,4)-β-glucan accumulation may be due to *cis*-regulated differences in *HvCslF6* expression, these could not be attributed to any specific DNA sequence polymorphism. Nevertheless, information on *HvCslF6* sequence polymorphism was used to develop molecular markers that could be used in barley breeding to select for the desired [low or high (1,3;1,4)-β-glucan] allele of the QTL.

**Electronic supplementary material:**

The online version of this article (doi:10.1007/s11032-015-0208-6) contains supplementary material, which is available to authorized users.

## Introduction

Cell walls of cereal crops and other grasses are characterised by the presence of (1,3;1,4)-β-glucans, which are polysaccharides that consist of unbranched and unsubstituted chains of β-d-glucosyl residues linked by both (1,3)- and (1,4)-β-glucosidic linkages. Cell walls in barley (*Hordeum* *vulgare* L.) grain are particularly rich in (1,3;1,4)-β-glucans, with levels ranging between 3 and 7 % by weight in the starchy endosperm of cultivars that are used for malting or for feed (Kato et al. 1995) and considerably higher levels having been detected in wild barley (Henry and Brown 1987).

There has been much interest in the levels of (1,3;1,4)-β-glucan in barley grain, mainly because of filtration difficulties attributed to these polysaccharides during the brewing process. Although barley breeders have generally selected against high levels of (1,3;1,4)-β-glucan in grain or malt, beneficial effects of (1,3;1,4)-β-glucans and other non-starchy polysaccharides in human health and nutrition have also been recognised (Braaten et al. 1994; Brennan and Cleary 2005). The (1,3;1,4)-β-glucans are constituents of dietary fibre, which is believed to greatly reduce the risk of serious human diseases, including type II diabetes, cardiovascular disease and colorectal cancer (Collins et al. 2010). Thus, there has recently been increasing interest in cereal grains with high levels of (1,3;1,4)-β-glucan.

Burton et al. (2006) demonstrated that (1,3;1,4)-β-glucan synthesis in the grasses is mediated, at least in part, by the *CslF* group of cellulose synthase-like genes. Barley has ten *HvCslF* genes (Schreiber et al. 2014), but transcript profiling has shown only two of these (*HvCslF6* and *HvCslF9*) to be transcribed at high levels in developing barley grain (Burton et al. 2008; Schreiber et al. 2014). For *HvCslF6*, transcript levels were high throughout endosperm development. For *HvCslF9*, transcript levels peaked at about 8 days after pollination (DAP) and decreased to very low levels by 15 DAP.

The products of *CslF6* genes are thought to be particularly important for (1,3;1,4)-β-glucan synthesis. In rice (*Oryza* *sativa* L.), *OsCslF6* knockout mutants synthesise very little (1,3;1,4)-β-glucan (Vega-Sánchez et al. 2012). In wheat (*Triticum* *aestivum* L.), (1,3;1,4)-β-glucan content is reduced by RNAi inhibition of *TaCslF6* (Nemeth et al. 2010) and is increased by the addition of barley chromosome 7H (on which *HvCslF6* is located) (Cseh et al. 2013). In barley, overexpression of *HvCslF6* has been shown to substantially increase (1,3;1,4)-β-glucan content (Burton et al. 2011), the lack of (1,3;1,4)-β-glucan synthesis in β-glucan-less (*bgl*) barley has been attributed to a specific mutation in a conserved region of *HvCslF6* (Taketa et al. 2012), and reduced (1,3;1,4)-β-glucan has been attributed to an induced mutation in *HvCslF6* (Hu et al. 2014).

Quantitative trait loci (QTL) contributing to grain (1,3;1,4)-β-glucan content have been mapped in several regions of the barley genome (Han et al. 1995; Igartua et al. 2002; Kim et al. 2011; Molina-Cano et al. 2007; Li et al. 2008; Cory et al. 2012; Islamovic et al. 2013; Steele et al. 2013). Some of these coincide with the genetic map locations of *HvCslF* genes (Burton et al. 2008). Notably, effects on grain (1,3;1,4)-β-glucan concentration have been detected near the *HvCslF6* gene on chromosome 7H using progeny from seven independent cross combinations: Derkado × B83-12/21/5 (Igartua et al. 2002), Beka × Logan (Molina-Cano et al. 2007), CDC Bold × TR251 (Li et al. 2008), H93174006 × Merit (Cory et al. 2012), Falcon × Azhul (Islamovic et al. 2013), Skardu Oldings × Static (Steele et al. 2013) and *m351* × Steptoe (Hu et al. 2014).

According to sequence data presented by Taketa et al. (2012), Cory et al. (2012) and Hu et al. (2014), there is some *HvCslF6* sequence variation between parents that have been used in mapping QTL for (1,3;1,4)-β-glucan concentration. None of the reported sequence differences occur in all of the parental combinations for which QTL for grain (1,3;1,4)-β-glucan concentration have been mapped on chromosome 7H, and only two alter the predicted amino acid sequence of the gene product. Both non-synonymous polymorphisms are in exon3 of *HvCslF6*. Both are single nucleotide polymorphisms (SNPs) that lead to alanine-threonine substitutions in HvCSLF6, one at position 590 (A590T) and the other at position 849 (A849T).

The SNP in the A590T codon (referred to as SNP23 by Taketa et al. (2012) and as SNP_4105 by Cory et al. (2012)) is known to be polymorphic within each of two pairs of parents (CDC Bold and TR251; H93174006 and Merit) for which QTL have been mapped at *HvCslF6*. In each case, the parent whose QTL allele reduces (1,3;1,4)-β-glucan concentration (CDC Bold or Merit) has the Ala^590^ residue. Cory et al. (2012) suggested that the A590T polymorphism might affect phosphorylation of the HvCSLF6 protein. However, according to sequence information provided by Taketa et al. (2012), this SNP is not polymorphic between two other pairs of parents for which grain (1,3;1,4)-β-glucan QTL have been mapped at the *HvCslF6* position: Derkado and B83-12/21/5 (Igartua et al. 2002) and Beka and Logan (Molina-Cano et al. 2007) and it is polymorphic between Steptoe (Thr^590^) and Morex (Ala^590^), for which no grain (1,3;1,4)-β-glucan QTL was detected on chromosome 7H (Han et al. 1995).

The SNP in the A849T codon was discovered in the induced mutant line *m351*, which has a low (1,3;1,4)-β-glucan concentration (Hu et al. 2014). The mutant has the Thr^849^ residue, while its parent cultivar Harrington has Ala^849^. According to Hu et al. (2014), position 849 is within a predicted transmembrane domain, and the replacement of alanine by threonine may affect the enzyme functionality by reducing protein stability.

To further investigate how the A590T substitution might affect the structure and function of the HvCSLF6 protein, we examined its position in relation to known features of the protein. To evaluate the effect of that substitution on (1,3;1,4)-β-glucan synthesis, we expressed the CDC Bold (Ala^590^) and TR251 (Thr^590^) alleles of *HvCslF6* in leaves of *Nicotiana* *benthamiana*. To investigate whether *HvCslF6*-associated QTL effects could be due to differential expression, we monitored *HvCslF6* transcript abundance in developing grain and sequenced the 3′ untranslated region (3′-UTR) and a putative promoter region of *HvCslF6*. We also developed new marker assays for sequence polymorphisms within *HvCslF6*.

## Materials and methods

### Protein modelling

The glycosyl transferase 2 PFAM domain (PF00535) and two flanking transmembrane helices of the *Rhodobacter* *sphaeroides* BcsA crystal structure (Morgan et al. 2013) were aligned to HvCSLF6 using Expresso (Armougom et al. 2006) and manually refined. The structure of non-homologous regions in the alignment were predicted using I-TASSER (Zhang 2008) and used together with BcsA as templates for producing homology models using Modeller (Šali and Blundell 1993). Models were assessed with Modellers DOPE and GA32 functions, and also with ProSA (Wiederstein and Sippl 2007). Top-scoring candidates were optimised using Modeller loop refinement scripts.

### Plant materials

The cultivars CDC Bold, Beka, Logan, Harrington, Morex, Steptoe, Azhul and CDC Fibar and the breeding lines TR251, TR306 and B83-12/21/3 were used in this research. CDC Bold, TR251, Beka, Logan, Derkado and B83-12/21/3 were used because they are the parents of populations in which QTL for grain (1,3;1,4)-β-glucan concentration had been mapped in the *HvCslF6* region Harrington, TR306, Morex and Steptoe were included as two additional pairs of mapping parents that exhibit the A590T polymorphism in HvCSLF6 (Taketa et al. 2012). CDC Fibar and Azhul were included as representatives of two other known alleles of *HvCslF6* (Taketa et al. 2012).

### Plant growth and tissue sampling

In each of three consecutive experiments, seeds of CDC Bold and TR251 were incubated on wet filter paper in the dark at 4 °C for 3 days. Germinated seeds were transferred to pots containing coconut peat, with 5 grains per pot and 5 pots per line. Plants were grown in a glasshouse in Urrbrae, South Australia, Australia, with the day and night temperatures maintained at 23 and 18 °C, respectively. In one of these experiments, the lines Beka, Logan, Harrington and TR306 were included in addition to CDC Bold and TR251. At the seedling stage, a sample of leaf tissue was taken from the third leaf of each plant, frozen in liquid nitrogen and stored at −80 °C.

As individual spikes reached anthesis, the date was recorded and the spikes were covered with a glycerol-embedded paper bag. In the first experiment, developing caryopses were collected from multiple spikes of CDC Bold and TR251 at 6, 8, 12, 16, 20 and 30 DAP. In the second experiment, developing caryopses were collected from multiple spikes of CDC Bold and TR251 every 2 days from 8 to 34 DAP. In addition, some of the CDC Bold and TR251 plants from that experiment were used to make reciprocal crosses, and developing F_1_ caryopses were collected at 16, 20 and 24 DAP. In the third experiment, developing caryopses were collected from multiple spikes of each of CDC Bold, TR251, Beka, Logan, Harrington and TR306 every 2 days from 8 to 38 DAP.

For caryopses collected at 6 or 8 DAP, the embryo was removed and the soft endosperm was squeezed out. For caryopses collected at 12 or more DAP, the maternal pericarp tissue was peeled off and the embryo removed. All extracted endosperm tissues were frozen in liquid nitrogen and stored at −80 °C. At maturity, samples of grain were harvested from each line. For each line, 10 mature grains were sampled at random and milled using a Retsch Mixer Mill MM400.

### DNA extraction, RNA extraction and cDNA synthesis

DNA was isolated from leaf samples using a DNA midi-prep method (Rogowsky et al. 1991) with modifications as described by Pallotta et al. (2000). For testing of marker assays, DNA was isolated from individual seeds of CDC Bold, TR251, Beka, Logan, Steptoe, Morex, B83-12/21/3, Derkado and Azhul using an OKTOPURE robot (LGC Limited, London, UK). A DNA sample of CDC Fibar was provided by Peter Eckstein of the University of Saskatchewan (Saskatoon, Canada).

Total RNA was extracted from samples of barley endosperm tissue (each obtained from at least three plants and consisting of between 50 and 100 mg as described in Burton et al. (2011). For samples from the first experiment, first-strand cDNA was synthesised using the SuperScript^®^ III First-Strand Synthesis System for RT-PCR kit from Invitrogen (Life Technologies, Carlsbad, CA), with the poly-dT replaced by poly-dT tagged with a specific sequence at the 5′ end [5′-ATT CTA GAG GCC GAG GCG GCC GAC ATG TTT (Tn17)]. For samples from the second and third experiments, cDNA was synthesised using SuperScript^®^ VILO™ cDNA synthesis kits from Invitrogen (Life Technologies, Carlsbad, CA), following the manufacturer’s protocol.

### Transient expression of *HvCslF6* in *Nicotianabenthamiana* leaves

Full-length (2.944 kb) cDNAs for *HvCslF6* were PCR-amplified from cDNA synthesised from 8-DAP endosperm from CDC Bold and TR251 using primers HvF6F8 (5′-GCCTGAGCCTGCCATTGTTGGAC-3′) and HvFDQR (5′-TGTCCGGGCAAAGTCATCAA-3′). Phusion High-Fidelity PCR Master Mix and HF Buffer (Thermo Fisher Scientific Australia Pty, Victoria, Australia) were used, with dimethyl sulfoxide (DMSO) adjusted to a total final concentration of 3 % (v/v). The denaturing step was 94 °C for 30 s, the annealing temperature was 66 °C for 30 s, the extension temperature was 72 °C for 90 s, and the thermal cycling steps were repeated for 39 cycles. The amplified full-length *HvCslF6* cDNAs were cloned into the PCR8^®^/GW/TOPO TA vector (Life Technologies, Carlsbad, CA) and subsequently recombined into the pEAQ-HT-DEST-1 vector (Sainsbury et al. 2009) using the Gateway^®^ LR Clonase™ II Enzyme Mix (Life Technologies, Carlsbad, CA).

Two constructs, pDEST-F6-CB (the pEAQ-HT-DEST-1 vector containing the full CDC Bold *HvCslF6* cDNA) and pDEST-F6-TR (the pEAQ-HT-DEST-1 vector containing the full TR251 *HvCslF6* cDNA) were introduced into *Agrobacterium* *tumefaciens* strain AGL1 by heat shock, yielding the strains AGL1:pDEST-F6-CB and AGL1:pDEST-F6-TR. To prepare concentrated infiltration solutions, AGL1:pDEST-F6-CB and AGL1:pDEST-F6-TR were spread on Luria–Bertani (LB) plates supplemented with kanamycin (50 µg/mL) and rifampicin (100 µg/mL) and grown at 28 °C for 2 days, then scraped from the plate into 50-mL Falcon tubes containing MM buffer (10 mM MgCl_2_, 10 mM MES), and adjusted to an OD_600_ of 1.0. Acetosyringone (100 µM) was added, and the samples were incubated at room temperature for 3 h prior to infiltration.

Leaves between 3 and 10 cm in length on 6-week-old plants of *N*. *benthamiana* were inoculated with AGL, AGL1:pDEST-F6-CB and AGL1:pDEST-F6-TR using 50-mL syringes. For each construct and for the control, three or four intact leaves on each of at least ten plants were evenly infiltrated. The inoculated plants were maintained in a greenhouse under natural light for a further 5 or 6 days. The inoculated leaves were collected, frozen in liquid nitrogen, freeze-dried and ground to a fine powder.

### Quantification of (1,3;1,4)-β-glucan

The concentration of (1,3;1,4)-β-glucan was measured in subsamples of the milled mature barley grain and *N*. *benthamiana* leaf tissue using the methods described by McCleary and Codd (1991), with minor modifications to permit analysis of small samples and using a 4.1 % (1,3;1,4)-β-glucan control flour provided with the Beta-Glucan (Mixed Linkage) assay kit (Megazyme International, Ireland). For the milled grain, analysis was conducted for three independent 15-mg subsamples for each barley line from each experiment. For the leaf tissue, samples were washed with 75 % (v/v) ethanol for 10 min at 95 °C to remove monosaccharides and chlorophyll, and analysis was conducted for one 20-mg sample from each inoculated leaf.

### Quantitative PCR

Samples of cDNA were subjected to quantitative PCR (Q-PCR) analysis for *HvCslF6*, with glyceraldehyde 3-phosphate dehydrogenase (GAPDH), heat shock protein (HSP70), cyclophilin and α-tubulin genes as controls for normalisation as used by Burton et al. (2008). Q-PCR reactions and normalisation were performed essentially as described by Burton et al. (2008), with three replicate amplifications carried out for each cDNA sample. For the cDNA samples from F_1_ endosperm, a primer pair (Forward: 5′-CCTCGTCGCTGGACATGGACAT-3′; Reverse: 5′-GGCACCGCTTTCGTCGGTCT-3′) that flanks a SNP in exon1 of *HvCslF6* [SNP1 as reported by Taketa et al. (2012)] was also used for Q-PCR.

### Analysis of the 3′ untranslated region of *HvCslF6*

To obtain the 3′ untranslated region of *HvCslF6* from CDC Bold and TR251, 3′ RACE (rapid amplification of cDNA ends; Scotto-Lavino et al. 2007) was performed using cDNA tagged with the 3′-PCR primer (5′-ATTCTAGAGGCCGAGGCGGCCGACATG-3′) and a 5′ internal primer within exon3 of *HvCslF6* (5′-CAGCACATACCTCCACCCGCT G-3′), with the following thermocycling conditions: initial denaturation at 95 °C for 2 min, 5 cycles of denaturation at 94 °C for 30 s and annealing at 72 °C for 1 min, 5 cycles of denaturation at 94 °C for 30 s and annealing at 70 °C for 1 min, 25 cycles of denaturation at 94 °C for 30 s and final annealing at 60 °C for 30 s, and a final extension at 68 °C for 1 min. Nucleotide sequencing of 3′ RACE amplicons was conducted at the Australian Genomics Research Facility (AGRF, Adelaide, Australia). The resulting sequences were used as BLAST queries against genomic sequence databases (assemblyWGSMorex, assembly_WGSBarke and assembly_WGSBowman) at the IPG Barley BLAST Server (http://webblast.ipk-gatersleben.de/barley/).

### Analysis of a putative promoter region of *HvCslF6*

To obtain the promoter region of *HvCslF6*, PCR primers designed based on sequence upstream from the open reading frame of the cultivar Morex were used to amplify a products of 2,996 bp from genomic DNA of CDC Bold and TR251. Amplicons were sequenced by the Australian Genome Research Facility (Adelaide, SA, Australia). Sequences were assembled and aligned using Geneious 6.1 (Biomatters, available from http://www.geneious.com).

### Development of KASP™ markers for *HvCslF6* sequence polymorphisms

Using Kraken™ software (LGC Limited, London, UK), primers were designed to develop KASP™ marker assays for six of the *HvCslF6* sequence polymorphisms that were reported by Taketa et al. (2012): five SNPs [SNP44, SNP12, SNP14 and SNP21 and an insertion–deletion (indel2)]. The resulting assays were applied to genomic DNA using an automated SNPLine system (LGC Limited, London, UK).

## Results

### Secondary structural predictions

**Fig. 1:**
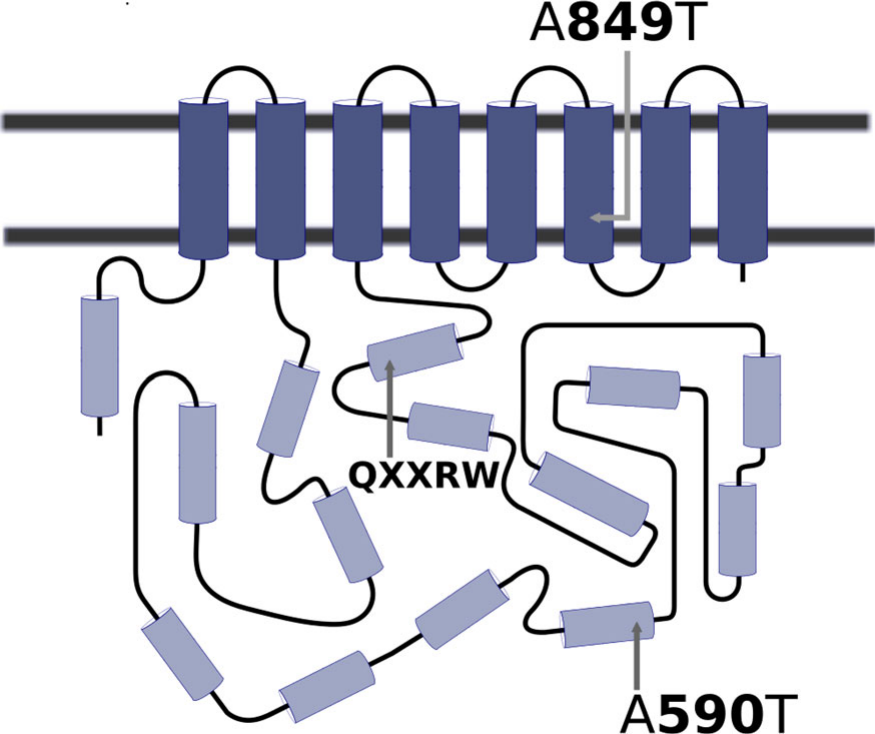
Structural prediction of HvCSLF6 estimating the positions of two alanine-to-threonine substitutions (A590T and A849T) relative to putative transmembrane helices (*dark* *cylinders*) and cytosolic helices (*light* *cylinders*) and the location a core catalytic motif (QXXRW)

Mapping of the two previously identified alanine-to-threonine substitutions in the HvCSLF6 protein onto a two-dimensional representation of the protein (Fig. 1) confirmed that the A849T substitution is located in a trans-membrane helix, in a region in which it could affect the stability of the protein in the plasma membrane and/or its interactions with other (as yet unidentified) partner proteins. In contrast, the A590T substitution lies in an alpha helix close to the surface of the cytosolic region and away from the catalytic site.

### Grain (1,3;1,4)-β-glucan concentration

The concentration of (1,3;1,4)-β-glucan in mature grain was consistently higher for TR251 than for CDC Bold (Online Resource 1). In the experiment in which two other pairs of mapping parents were included, the (1,3;1,4)-β-glucan concentration was higher for Harrington than for TR306 and higher for Beka than for Logan.

### Transient expression of *HvCslF6* in *Nicotianabenthamiana* leaves

**Fig. 2:**
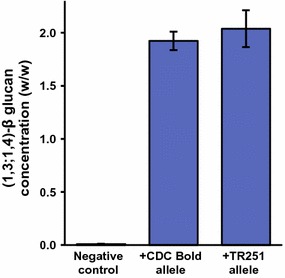
Mean (±SE) concentration of (1,3;1,4)-β-glucan in leaves of *Nicotiana* *benthamiana* after infiltration with *Agrobacterium* *tumefaciens* strains AGL1 (a negative control), AGL1:pDEST-F6-CB (carrying the CDC Bold allele of *HvCslF6*) and AGL1:pDEST-F6-TR (carrying the TR251 allele of *HvCslF6*)

In leaves of *N*. *benthamiana* infiltrated with *Agrobacterium* strain AGL1, the mean concentration of beta-glucan was <0.02 % (w/w). Significantly (*p* < 0.0001) higher concentrations were found in leaves infiltrated with AGL1:pDEST-F6-CB (1.9 % w/w) or Agl1:pDEST-F6-TR (2.0 % w/w) (Fig. 2), with no significant difference (*p* > 0.05) between the mean concentration in leaves expressing the product of the CDC Bold allele of *HvCslF6* and those expressing the product of the TR251 allele.

### Transcript profiles during grain development

**Fig. 3:**
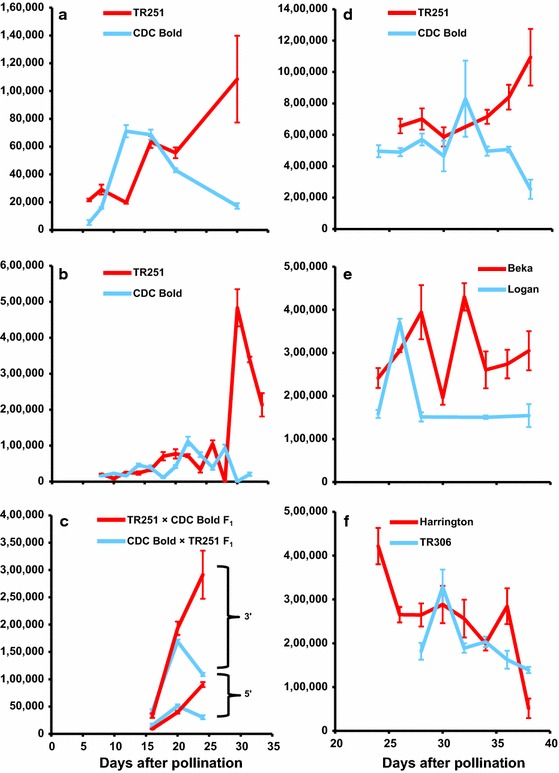
Mean normalised transcript levels (±SD) of *HvCslF6* in developing endosperm of: TR251 and CDC Bold (**a**, **b**, **d**); F_1_ seeds from reciprocal crosses between TR251 and CDC Bold (**c**); Beka and Logan (**e**) and Harrington and TR306 (**f**). The results shown are for endosperm tissue sampled from glasshouse experiments shown in February 2010 (**a**), March 2012 (**b**, **c**) and June 2012 (**d**–**f**). For the endosperm from F_1_ seeds (**c**), Q-PCR primers designed to flank an exonic SNP near the 5′ end of the gene (5′) were used in addition to standard Q-PCR primers complementary to a region near the 3′ end of the gene (3′)

In endosperm tissues sampled late in grain development (at 30 DAP in the first experiment, from 30 to 34 DAP in the second experiment and from 34 to 38 DAP in the third experiment), *HvCslF6* transcript levels were much higher in TR251 [the line with higher grain (1,3;1,4)-β-glucan concentration] than in CDC Bold (Fig. 3a, b, d). In the third experiment, in which Beka, Logan, Harrington and TR306 were also included, a similar difference was observed between Beka and Logan (between 28 and 38 DAP) (Fig. 3e) but not between Harrington and TR306 (Fig. 3f).

**Fig. 4:**
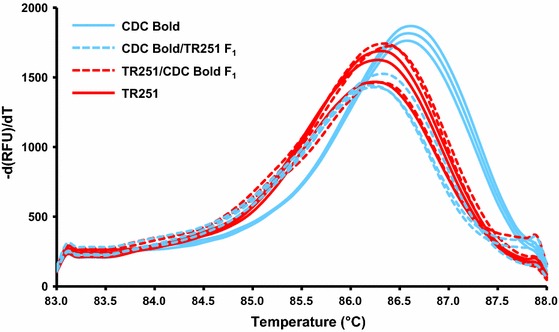
Melt curves for CDC Bold, TR251 and their reciprocal F_1_ crosses. Melt curves were acquired from 83 to 88.0 °C with an increment of 0.1 °C per 5 s, after amplification from cDNA using primers that flank an exonic SNP near the 5′ end of *HvCslF6*

The F_1_ seeds from reciprocal crosses between TR251 and CDC Bold developed and dried down more rapidly than seeds on plants that were allowed to self-pollinate. This may be due to desiccation effects from partial removal of the lemma and palea during crossing. At 24 DAP (when the F_1_ seeds were nearly mature), there was a distinct difference in *HvCslF6* transcript abundance between these reciprocals (Fig. 3c), with much more *HvCslF6* transcript observed in the tissue from seeds for which TR251 was the maternal parent (TR251 × CDC Bold) than for the reciprocal cross (CDC Bold × TR251). This difference was evident for both primer pairs, even though the products amplified with the primers flanking a SNP near the 5′ end of the gene were less abundant than those with the standard primers near the 3′ end of the gene. In both types of F_1_, the melt curves for the products amplified from the SNP-containing region were similar to those for TR251 (*T*_m_ = 86.3 °C) and different from those for CDC Bold (*T*_m_ = 86.6 °C (Fig. 4).

### Analysis of a 3′-untranslated region

Using 3′ RACE, identical 204-bp 3′-UTR regions of *HvCslF6* were sequenced from CDC Bold and TR251. Use of this sequence as a query in a BLASTN search against the assembly_WGSMorex, assembly_WGSBarke and assembly_WGSBowman databases at the IPK Barley BLAST Server (http://webblast.ipk-gatersleben.de/barley/) retrieved three contigs (morex_contig_41513, barke_contig_1801451 and bowman_contig_1981203), each of which also contained an identical 204-bp region (Online Resource 2).

### Analysis of a putative promoter of *HvCslF6*

Within a 2,996-bp putative promoter region upstream from the ATG translation start site of *HvCslF6*, the sequence of TR251 was found to differ from that of CDC Bold by 12 single nucleotide polymorphisms and one eight-nucleotide insertion (Online Resource 3). The Beka, Logan and TR306 sequences in this region were identical to those of CDC Bold, while Harrington differed from CDC Bold by one SNP.

### Marker assays for *HvCslF6* polymorphisms

KASP™ assays (Online Resource 4) were developed for six sequence polymorphisms within *HvCslF6* (Online Resource 5). With just five SNP assays, it is possible to distinguish among almost all *HvCslF6* alleles that are known to occur naturally in cultivated barley (Table 1). The exceptions are the alleles carried by Azhul, B83-12/21/3 and CDC Fibar, which cannot be distinguished from each other with any of the assays developed here (Table 1). According to Taketa et al. (2012), those alleles differ only at SSR1 [with (CATT)_2_ in Azhul vs. (CATT)_3_ in CDC Fibar and B83-12/21/3] and at indel 4 [with (T)_10_ in CDC Fibar vs. (T)_9_ in Azhul and B83-12/21/3].

**Table 1:** Marker genotypes detected in barley lines using KASP™ assays designed to detect six DNA polymorphisms within the *HvCslF6* gene of barley

Marker	Polymorphism^a^	CDC Bold	TR251	Beka	Logan	Harrington	TR306	Steptoe	Morex	B83-12/21/3	Derkado	Azhul	CDC Fibar
*wri51*	558 (SNP4)	G	G	A	G	A	G	G	G	A	A	A	A
*wri52*	1,137 (indel2)^b^	No	∆14	No	No	No	∆14	∆14	No	No	No	No	No
*wri53*	1,398 (SNP12)	G	G	G	G	A	G	G	G	A	G	A	A
*wri54*	1,656 (SNP14)	T	T	T	T	T	T	T	C	T	T	T	T
*wri55*	3,262 (SNP 21)	G	A	A	G	A	–^c^	A	G	A	A	A	A
*wri56*	4,064 (SNP23)	G	A	G	G	G	A	A	G	G	G	G	G

^a^Nucleotide position and SNP or indel designation from Taketa et al. (2012)

^b^The sequence of this 14-bp indel is CCATGAGAAGGAGG

^c^No amplification

## Discussion

Given that QTL affecting grain (1,3;1,4)-β-glucan concentration have been mapped at the *HvCslF6* locus in numerous populations of barley (Han et al. 1995; Igartua et al. 2002; Kim et al. 2011; Molina-Cano et al. 2007; Li et al. 2008; Cory et al. 2012; Islamovic et al. 2013; Steele et al. 2013), there must be one or more DNA sequence polymorphisms in or near *HvCslF6* that influence either the synthesis or degradation of grain (1,3;1,4)-β-glucan. Given the evidence that CSLF6 enzymes are themselves essential for (1,3;1,4)-β-glucan synthesis (Taketa et al. 2012; Vega-Sánchez et al. 2012), it is reasonable to hypothesise that the causal polymorphism(s) are within *HvCslF6* itself or at some closely linked sequence that plays a role in regulating *HvCslF6* transcription or translation.

While there is good evidence that certain induced mutations within *HvCslF6* are responsible for the beta-glucanless phenotype in the mutants OUM125, KM27 and KM30 (Taketa et al. 2012) and for the low-(1,3;1,4)-β-glucan phenotype of the mutant *m351* (Hu et al. 2014), the evidence for effects of naturally occurring *HvCslF6* sequence differences is less convincing. Although there are numerous naturally occurring sequence polymorphisms (Taketa et al. 2012) within *HvCslF6*, only one of these affects the protein sequence, and none are consistently present in the parental combinations for which QTL have been mapped at *HvCslF6*. Among populations in which QTL have been mapped at *HvCslF6*, some (CDC Bold × TR251 and H93174006 × Merit) segregate for a SNP that causes an A590T substitution, while others (Beka × Logan and Derkado × B83-12/21/5) do not. Cory et al. (2012) noted that other pairs of mapping parents (Steptoe and Morex; Harrington and TR306) also exhibit the A590T polymorphism. They interpreted this as supportive of the causal effect of the A590T difference, citing Han et al. (1995) and Mather et al. (1997) for reports of (1,3;1,4)-β-glucan QTL on chromosome 7H. However, those QTL were for (1,3;1,4)-β-glucan extracted from malt, not unmalted grain. Given the potential effects of differential (1,3;1,4)-β-glucanase activity during malting, differences in malt (1,3;1,4)-β-glucan concentration cannot be assumed to be due to differences in grain (1,3;1,4)-β-glucan concentration. In the Steptoe × Morex population, QTL for grain (1,3;1,4)-β-glucan concentration were mapped on chromosomes 1H and 2H but not 7H (Han et al. 1995). In the Harrington × TR306 population, grain (1,3;1,4)-β-glucan concentration was not measured (Mather et al. 1997).

Without an X-ray crystal structure of the HvCSLF6 protein or reliable means of modelling large membrane-bound proteins, it is difficult to predict whether specific amino acid substitutions would affect enzyme activity. Nevertheless, the position of the A890T substitution within a predicted transmembrane domain makes it likely that this difference could affect protein stability and thus enzyme functionality. This region could also be involved in interactions with other partner proteins, if the CSL proteins, like other cellulose synthase proteins, act in complex. In contrast, the A590T substitution seems conservative in that it is not located in any of the eight predicted trans-membrane helices of the protein, nor is it close to the D, D, D, QXXRW amino acid residues that are believed to be involved in catalysis (Richmond and Somerville 2000) or a region of the protein that is found in HvCSLF6 but not in other HvCSL proteins (Burton et al. 2008). Although it is possible that alanine–threonine substitutions could create N-glycosylation, phosphorylation (as suggested by Cory et al. 2012) or other post-translational modification sites, such sites are numerous in a protein of this size.

Consistent with the idea that A590T substitution does not directly affect (1,3;1,4)-β-glucan synthesis, we found that transient expression of the CDC Bold and TR251 alleles led to similar amounts of (1,3;1,4)-β-glucan accumulation in the leaves of *N*. *benthamiana*.

Considering all of the points discussed above, it seems unlikely that the A590T substitution is responsible for QTL detected at or near *HvCslF6*. Another way in which the *HvCslF6* gene could affect grain (1,3;1,4)-β-glucan concentration is through differential expression. Here, this possibility was investigated by comparing the abundance of *HvCslF6* gene transcripts in developing endosperm of CDC Bold and TR251. Based on the transcript levels detected here, it seems that quite low levels of full-length *HvCslF6* transcript are sufficient to support the synthesis of significant amounts of (1,3;1,4)-β-glucan, which in turn constitutes about 70 % by weight of the starchy endosperm walls of barley (Fincher 1975). There is some precedence for this situation, insofar as Dhugga et al. (2004) reported that very low levels of *CslA* mRNA (15 transcripts from a total of 15,000 ESTs) supported the synthesis of very large amounts of mannans in developing guar seeds. If the HvCSLF6 enzyme has a long half-life in the endomembrane system of the cell, slow but steady transcription of the gene could result in the gradual accumulation of high levels of the enzyme. Consistent with this, when rice *OsCslF* genes were expressed in transgenic *Arabidopsis* plants, it took several weeks before (1,3;1,4)-β-glucan could be detected in young leaves (Burton et al. 2006).

In each of three experiments conducted here, *HvCslF6* transcripts were more abundant for TR251 than for CDC Bold during very late stages of endosperm development. A similar difference was observed between Beka and Logan, but not between Harrington and TR306. Based on these results, it seems that the quantitative effects detected for grain (1,3;1,4)-β-glucan concentration at or near *HvCslF6* in the CDC Bold × TR251 and Beka × Logan populations could be due to *cis*-regulated differences in *HvCslF6* transcript abundance late in endosperm development. Such differences could be due to differential transcription of *HvCslF6* and/or to differential degradation of *HvCslF6* transcripts. Although it is somewhat surprising to observe such differences very late in grain development, when the grain would be starting to dry out and starchy endosperm cells would be senescing, they are consistent with previous reports (Coles 1979; Wilson et al. 2012) of (1,3;1,4)-β-glucan accumulation accelerating late in grain development (more than 25 days after pollination).

Consistent with the difference in *HvCslF6* transcript abundance between TR251 and CDC Bold, there was a difference between the reciprocal crosses TR251 × CDC Bold and CDC Bold × TR251. Although this could be due to a maternal effect (greater *HvCslF6* expression in endosperm developing on TR251 plants than in endosperm developing on CDC Bold plants), it seems more likely to be due to an allele dosage effect in the triploid endosperm, in combination with differential expression between the alleles. The exons of the two alleles differ by just three widely separated SNPs, precluding the use of allele-specific Q-PCR to quantify transcripts of individual alleles. Nevertheless, a difference in *T*_m_ between CDC Bold and TR251 amplicons from a SNP-containing region in exon1 of *HvCslF6* made it possible to detect that the cDNA of both reciprocal F_1_s was more similar to that of TR251 than to that of CDC Bold, indicating the transcripts in the hybrid endosperm tissue predominantly represent the TR251 allele, regardless of allele dosage. This observation is consistent with there being *cis*-regulated differential expression between the two alleles. To further investigate regulation of *HvCslF6*, transcript abundance could be quantified for members of a mapping population and expression QTL (eQTL) could be mapped. This was not undertaken here, due to the substantial cost and effort of assessing transcript abundance in developing endosperm of large numbers of plants and also because the results of such an experiment would not be definitive. If (as might be expected) an eQTL was detected in the *HvCslF6* region, this would confirm *cis*-regulation of *HvCslF6* but would not reveal the mechanism of this regulation. If an eQTL was detected elsewhere in the genome (but not at *HvCslF6*), this would demonstrate *trans*-regulation of *HvCslF6*, without shedding any light on the cause of the cause of the grain (1,3;1,4)-β-glucan QTL on chromosome 7H.

To investigate possible causes of the observed differential transcript abundance, a 204-bp 3′-UTR and a putative promoter region for *HvCslF6* were isolated and sequenced from several mapping parents. Neither of these revealed any possible causes for differential transcript abundance between parents with high and low grain (1,3;1,4)-β-glucan. The 3′-UTR sequence was completely conserved across all of the lines sequenced. Within the putative promoter, there were 11 SNPs between CDC Bold and TR251, but none between Beka and Logan.

Although the results presented here indicate that differential abundance of *HvCslF6* transcripts late in endosperm development could be involved in a QTL effect for grain (1,3;1,4)-β-glucan concentration in barley, neither the QTL nor the differential transcript levels could be attributed to any particular sequence polymorphism within *HvCslF6*. Possibly, the causal sequence difference is not within *HvCslF6* itself; it may be in some other closely linked gene or regulatory sequence.

Regardless of whether *HvCslF6* allelic sequence variation has any direct effect on quantitative differences in grain (1,3;1,4)-β-glucan concentration, it is clear from previous reports that the *HvCslF6* region of chromosome 7H can have significant effects on that trait. Accordingly, molecular markers based on *HvCslF6* polymorphisms could be useful for selection of the desired QTL alleles in barley breeding. As there are no sequence polymorphisms in common across all parental combinations for which a QTL has been detected on 7H, no single *HvCslF6*-based molecular marker will be useful in all genetic backgrounds. Cory et al. (2012) developed several markers that could be useful for distinguishing the allele carried by TR251, but they do not distinguish among other possible alleles. The lines tested here include representatives of seven of the eight naturally occurring alleles reported by Taketa et al. (2012). Among those seven alleles, three could not be distinguished from each other by the assays developed here. Failure to develop assays to distinguish reliably among Azhul, B83-12/21/3 and CDC Fibar can be attributed to the fact that they differ from each other only for minor sequence repeats. Although the allele carried by Nishinohoshi was not represented in our panel, it should be readily distinguishable from other alleles based using the assays developed here, as its allele sequence differs from each of the other known alleles at one or more of the positions assayed by these markers. The KASP™ markers presented here provide a set of fluorescence-based assays from which barley breeders can select appropriate assays for different cross combinations. Similar assays could probably be designed to detect induced mutations such as those reported by Taketa et al. (2012) and Hu et al. (2014).

## Electronic supplementary material

Below is the link to the electronic supplementary material.

Concentration of (1,3;1,4)-β-glucan in mature barley grain harvested from three glasshouse experiments (PDF 206 kb)

Annotated sequence of a 204-bp pre-mRNA 3′ untranslated region of *HvCslF6* obtained by 3′ RACE from cDNA of CDC Bold and TR251 barley (PDF 214 kb)

Alignment of sequences from TR251, CDC Bold, Beka, Logan, TR306 and Harrington for a putative promoter region of *HvCslF6* (PDF 2264 kb)

KASP™ marker assays designed to detect six DNA polymorphisms within the *HvCslF6* gene of barley (PDF 118 kb)

Alignment of sequences for eight alleles of *HvCslF6*, showing the positions of polymorphisms for which marker assays were developed (PDF 245 kb)
