# Supplementary material for: Differential expression of the HvCslF6 gene late in grain development may explain quantitative differences in (1,3;1,4)-β-glucan concentration in barley
Source: Mol Breed. 2015 Jan 20;35(1):20. doi: 10.1007/s11032-015-0208-6 (PMC4298655; doi:10.1007/s11032-015-0208-6)
Supplement: Supplementary file 1 — Concentration of (1,3;1,4)-β-glucan in mature barley grain harvested from three glasshouse experiments (PDF 206 kb) [file 11032_2015_208_MOESM1_ESM.pdf]

Article	Differential expression of the *HvCslF6* gene late in grain development may explain quantitative differences in (1,3;1,4)-β-glucan concentration in barley
**Journal**	Molecular Breeding
**Authors**	Sie Chuong Wong, Rachel A Burton, Neil J Shirley, Alan Little, Julian Schwerdt, Kelvin H.P. Khoo, Geoffrey B Fincher and Diane E. Mather
**Corresponding author**	Diane E. Mather (diane.mather@adelaide.edu.au) School of Agriculture, Food and Wine and ARC Centre of Excellence in Plant Cell Walls, Waite Research Institute, University of Adelaide

Online Resource 1 Concentration of (1,3;1,4)-β-glucan in mature barley grain harvested from three glasshouse experiments

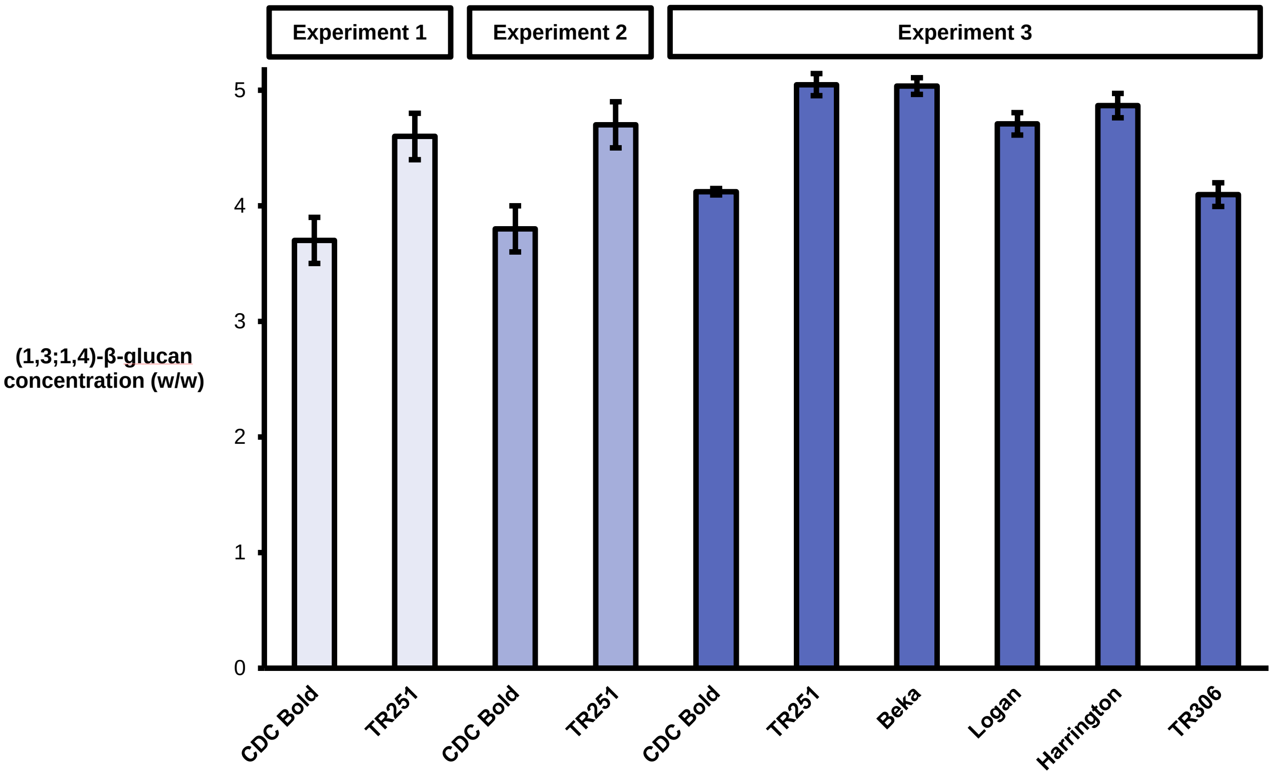

Fig. S1 Mean concentration (w/w) of (1,3;1,4)-β-glucan (± standard error) in mature barley grain harvested from glasshouse experiments sown in February 2010 (Experiment 1) and March 2012 (Experiment 2) and June 2012 (Experiment 3)
