# Supplementary material for: Differential expression of the HvCslF6 gene late in grain development may explain quantitative differences in (1,3;1,4)-β-glucan concentration in barley
Source: Mol Breed. 2015 Jan 20;35(1):20. doi: 10.1007/s11032-015-0208-6 (PMC4298655; doi:10.1007/s11032-015-0208-6)
Supplement: Supplementary file 2 — Annotated sequence of a 204-bp pre-mRNA 3′ untranslated region of HvCslF6 obtained by 3′ RACE from cDNA of CDC Bold and TR251 barley (PDF 214 kb) [file 11032_2015_208_MOESM2_ESM.pdf]

| | |
|---|---|
| **Article** | Differential expression of the *HvCslF6* gene late in grain development may explain quantitative differences in (1,3;1,4)-β-glucan concentration in barley |
| **Journal** | Molecular Breeding |
| **Authors** | Sie Chuong Wong, Rachel A Burton, Neil J Shirley, Alan Little, Julian Schwerdt, Kelvin H.P. Khoo, Geoffrey B Fincher and Diane E. Mather |
| **Corresponding author** | Diane E. Mather (diane.mather@adelaide.edu.au) School of Agriculture, Food and Wine and ARC Centre of Excellence in Plant Cell Walls, Waite Research Institute, University of Adelaide |

**Online Resource 2** Annotated sequence of a 204-bp pre-mRNA 3' untranslated region of *HvCslF6* obtained by 3' RACE from cDNA of CDC Bold and TR251 barley

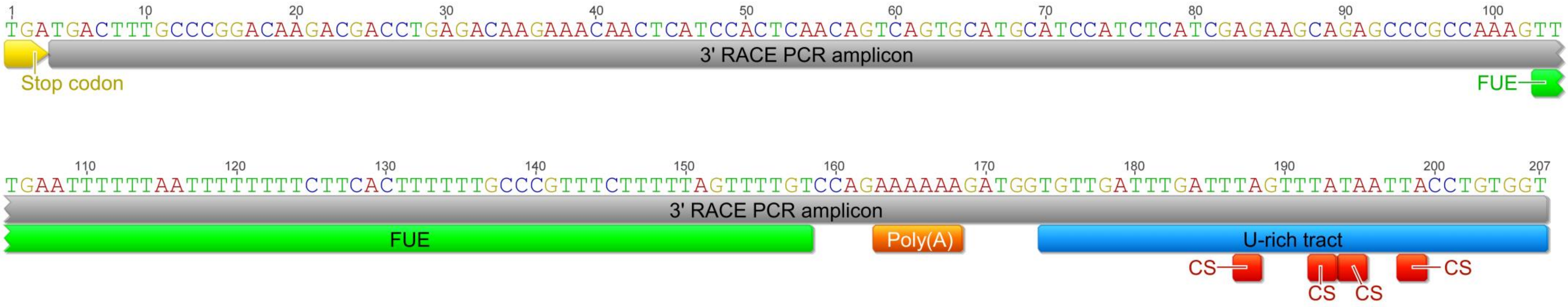


**Fig. S2** Annotated sequence of a 204-bp pre-mRNA 3' untranslated region of *HvCslF6* obtained by 3' RACE from cDNA of CDC Bold and TR251 barley. The far upstream element (FUE, green block) region contains multiple ambiguous UG motifs and is generally located 29 bp upstream of the mRNA cleavage site (CS, red blocks). The poly(A) signal (Poly(A), orange block) of *HvCslF6* is a AAAAAA hexamer. The U-rich tract (blue block) contains multiple putative mRNA cleavage sites which are denoted by TA di-nucleotide motifs at positions 187-188, 192-193, 194-195, or 198-199.
