# Supplementary material for: Differential expression of the HvCslF6 gene late in grain development may explain quantitative differences in (1,3;1,4)-β-glucan concentration in barley
Source: Mol Breed. 2015 Jan 20;35(1):20. doi: 10.1007/s11032-015-0208-6 (PMC4298655; doi:10.1007/s11032-015-0208-6)
Supplement: Supplementary file 3 — Alignment of sequences from TR251, CDC Bold, Beka, Logan, TR306 and Harrington for a putative promoter region of HvCslF6 (PDF 2264 kb) [file 11032_2015_208_MOESM3_ESM.pdf]

**Article**	Differential expression of the *HvCslF6* gene late in grain development may explain quantitative differences in (1,3;1,4)-β-glucan concentration in barley
**Journal**	Molecular Breed (ing
**Authors**	Sie Chuong Wong, Rachel A Burton, Neil J Shirley, Alan Little, Julian Schwerdt, Kelvin H.P. Khoo, Geoffrey B Fincher and Diane E. Mather
**Corresponding author**	Diane E. Mather (diane.mather@adelaide.edu.au) School of Agriculture, Food and Wine and ARC Centre of Excellence in Plant Cell Walls, Waite Research Institute, University of Adelaide

Online Resource 3 Alignment of sequences from TR251, CDC Bold, Beka, Logan, TR306 and Harrington for a putative promoter region of *HvCslF6*.

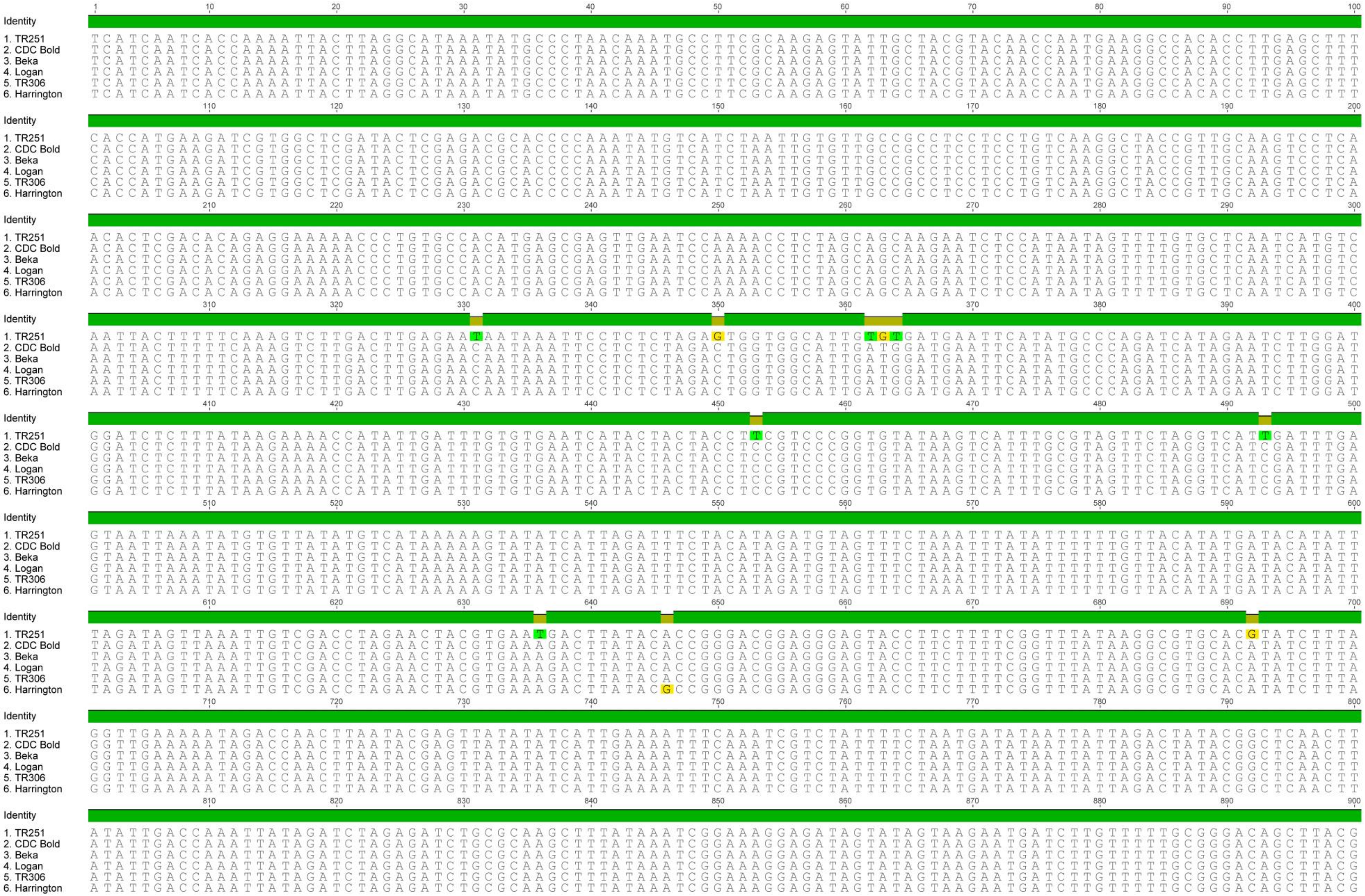

1 10 20 30 40 50 60 70 80 90 100
Identity
1. TR251 TCATCAATCACCAAAATTACTTAGGCATAAATATGCCCTAACAAATGCCTTCGCAAGAGTATTGCTACGTACAACCAATGAAGGCCACACCTTGAGCTTT
2. CDC Bold TCATCAATCACCAAAATTACTTAGGCATAAATATGCCCTAACAAATGCCTTCGCAAGAGTATTGCTACGTACAACCAATGAAGGCCACACCTTGAGCTTT
3. Beka TCATCAATCACCAAAATTACTTAGGCATAAATATGCCCTAACAAATGCCTTCGCAAGAGTATTGCTACGTACAACCAATGAAGGCCACACCTTGAGCTTT
4. Logan TCATCAATCACCAAAATTACTTAGGCATAAATATGCCCTAACAAATGCCTTCGCAAGAGTATTGCTACGTACAACCAATGAAGGCCACACCTTGAGCTTT
5. TR306 TCATCAATCACCAAAATTACTTAGGCATAAATATGCCCTAACAAATGCCTTCGCAAGAGTATTGCTACGTACAACCAATGAAGGCCACACCTTGAGCTTT
6. Harrington TCATCAATCACCAAAATTACTTAGGCATAAATATGCCCTAACAAATGCCTTCGCAAGAGTATTGCTACGTACAACCAATGAAGGCCACACCTTGAGCTTT
110 120 130 140 150 160 170 180 190 200
Identity
1. TR251 CACCATGAAGATCGTGGCTCGATACTCGAGACGCACCCCAAATATGTCATCTAATTGTGTTGCCGCCTCCTCCTGTCAAGGCTACCGTTGCAAGTCCTCA
2. CDC Bold CACCATGAAGATCGTGGCTCGATACTCGAGACGCACCCCAAATATGTCATCTAATTGTGTTGCCGCCTCCTCCTGTCAAGGCTACCGTTGCAAGTCCTCA
3. Beka CACCATGAAGATCGTGGCTCGATACTCGAGACGCACCCCAAATATGTCATCTAATTGTGTTGCCGCCTCCTCCTGTCAAGGCTACCGTTGCAAGTCCTCA
4. Logan CACCATGAAGATCGTGGCTCGATACTCGAGACGCACCCCAAATATGTCATCTAATTGTGTTGCCGCCTCCTCCTGTCAAGGCTACCGTTGCAAGTCCTCA
5. TR306 CACCATGAAGATCGTGGCTCGATACTCGAGACGCACCCCAAATATGTCATCTAATTGTGTTGCCGCCTCCTCCTGTCAAGGCTACCGTTGCAAGTCCTCA
6. Harrington CACCATGAAGATCGTGGCTCGATACTCGAGACGCACCCCAAATATGTCATCTAATTGTGTTGCCGCCTCCTCCTGTCAAGGCTACCGTTGCAAGTCCTCA
210 220 230 240 250 260 270 280 290 300
Identity
1. TR251 ACACTCGACACAGAGGAAAAACCCTGTGCCACATGAGCGAGTTGAATCCAAAACCTCTAGCAGCAAGAATCTCCATAATAGTTTTGTGCTCAATCATGTC
2. CDC Bold ACACTCGACACAGAGGAAAAACCCTGTGCCACATGAGCGAGTTGAATCCAAAACCTCTAGCAGCAAGAATCTCCATAATAGTTTTGTGCTCAATCATGTC
3. Beka ACACTCGACACAGAGGAAAAACCCTGTGCCACATGAGCGAGTTGAATCCAAAACCTCTAGCAGCAAGAATCTCCATAATAGTTTTGTGCTCAATCATGTC
4. Logan ACACTCGACACAGAGGAAAAACCCTGTGCCACATGAGCGAGTTGAATCCAAAACCTCTAGCAGCAAGAATCTCCATAATAGTTTTGTGCTCAATCATGTC
5. TR306 ACACTCGACACAGAGGAAAAACCCTGTGCCACATGAGCGAGTTGAATCCAAAACCTCTAGCAGCAAGAATCTCCATAATAGTTTTGTGCTCAATCATGTC
6. Harrington ACACTCGACACAGAGGAAAAACCCTGTGCCACATGAGCGAGTTGAATCCAAAACCTCTAGCAGCAAGAATCTCCATAATAGTTTTGTGCTCAATCATGTC
310 320 330 340 350 360 370 380 390 400
Identity
1. TR251 AATTACTTTTTCAAAGTCTTGACTTGAGAATAATAAATTCCTCTCTAGAGTGGTGGCATTGTGTGATGAATTCATATGCCCAGATCATAGAATCTTGGAT
2. CDC Bold AATTACTTTTTCAAAGTCTTGACTTGAGAACAATAAATTCCTCTCTAGACTGGTGGCATTGATGGATGAATTCATATGCCCAGATCATAGAATCTTGGAT
3. Beka AATTACTTTTTCAAAGTCTTGACTTGAGAACAATAAATTCCTCTCTAGACTGGTGGCATTGATGGATGAATTCATATGCCCAGATCATAGAATCTTGGAT
4. Logan AATTACTTTTTCAAAGTCTTGACTTGAGAACAATAAATTCCTCTCTAGACTGGTGGCATTGATGGATGAATTCATATGCCCAGATCATAGAATCTTGGAT
5. TR306 AATTACTTTTTCAAAGTCTTGACTTGAGAACAATAAATTCCTCTCTAGACTGGTGGCATTGATGGATGAATTCATATGCCCAGATCATAGAATCTTGGAT
6. Harrington AATTACTTTTTCAAAGTCTTGACTTGAGAACAATAAATTCCTCTCTAGACTGGTGGCATTGATGGATGAATTCATATGCCCAGATCATAGAATCTTGGAT
410 420 430 440 450 460 470 480 490 500
Identity
1. TR251 GGATCTCTTTATAAGAAAACCATATTGATTTGTGTGAATCATACTACTACCTTCGTCCCGGTGTATAAGTCATTTGCGTAGTTCTAGGTCATTGATTTGA
2. CDC Bold GGATCTCTTTATAAGAAAACCATATTGATTTGTGTGAATCATACTACTACCTCCGTCCCGGTGTATAAGTCATTTGCGTAGTTCTAGGTCATCGATTTGA
3. Beka GGATCTCTTTATAAGAAAACCATATTGATTTGTGTGAATCATACTACTACCTCCGTCCCGGTGTATAAGTCATTTGCGTAGTTCTAGGTCATCGATTTGA
4. Logan GGATCTCTTTATAAGAAAACCATATTGATTTGTGTGAATCATACTACTACCTCCGTCCCGGTGTATAAGTCATTTGCGTAGTTCTAGGTCATCGATTTGA
5. TR306 GGATCTCTTTATAAGAAAACCATATTGATTTGTGTGAATCATACTACTACCTCCGTCCCGGTGTATAAGTCATTTGCGTAGTTCTAGGTCATCGATTTGA
6. Harrington GGATCTCTTTATAAGAAAACCATATTGATTTGTGTGAATCATACTACTACCTCCGTCCCGGTGTATAAGTCATTTGCGTAGTTCTAGGTCATCGATTTGA
510 520 530 540 550 560 570 580 590 600
Identity
1. TR251 GTAATTAAATATGTGTTATATGTCATAAAAAGTATATCATTAGATTTCTACATAGATGTAGTTTCTAAATTTATATTTTTTGTTACATATGATACATATT
2. CDC Bold GTAATTAAATATGTGTTATATGTCATAAAAAGTATATCATTAGATTTCTACATAGATGTAGTTTCTAAATTTATATTTTTTGTTACATATGATACATATT
3. Beka GTAATTAAATATGTGTTATATGTCATAAAAAGTATATCATTAGATTTCTACATAGATGTAGTTTCTAAATTTATATTTTTTGTTACATATGATACATATT
4. Logan GTAATTAAATATGTGTTATATGTCATAAAAAGTATATCATTAGATTTCTACATAGATGTAGTTTCTAAATTTATATTTTTTGTTACATATGATACATATT
5. TR306 GTAATTAAATATGTGTTATATGTCATAAAAAGTATATCATTAGATTTCTACATAGATGTAGTTTCTAAATTTATATTTTTTGTTACATATGATACATATT
6. Harrington GTAATTAAATATGTGTTATATGTCATAAAAAGTATATCATTAGATTTCTACATAGATGTAGTTTCTAAATTTATATTTTTTGTTACATATGATACATATT
610 620 630 640 650 660 670 680 690 700
Identity
1. TR251 TAGATAGTTAAATTGTCGACCTAGAACTACGTGAATGACTTATACACCGGGACGGAGGGAGTACCTTCTTTTCGGTTTATAAGGCGTGCACGTATCTTTA
2. CDC Bold TAGATAGTTAAATTGTCGACCTAGAACTACGTGAAAGACTTATACACCGGGACGGAGGGAGTACCTTCTTTTCGGTTTATAAGGCGTGCACATATCTTTA
3. Beka TAGATAGTTAAATTGTCGACCTAGAACTACGTGAAAGACTTATACACCGGGACGGAGGGAGTACCTTCTTTTCGGTTTATAAGGCGTGCACATATCTTTA
4. Logan TAGATAGTTAAATTGTCGACCTAGAACTACGTGAAAGACTTATACACCGGGACGGAGGGAGTACCTTCTTTTCGGTTTATAAGGCGTGCACATATCTTTA
5. TR306 TAGATAGTTAAATTGTCGACCTAGAACTACGTGAAAGACTTATACACCGGGACGGAGGGAGTACCTTCTTTTCGGTTTATAAGGCGTGCACATATCTTTA
6. Harrington TAGATAGTTAAATTGTCGACCTAGAACTACGTGAAAGACTTATACGCCGGGACGGAGGGAGTACCTTCTTTTCGGTTTATAAGGCGTGCACATATCTTTA
710 720 730 740 750 760 770 780 790 800
Identity
1. TR251 GGTTGAAAAATAGACCAACTTAATACGAGTTATATATCATTGAAAATTTCAAATCGTCTATTTTCTAATGATATAATTATTAGACTATACGGCTCAACTT
2. CDC Bold GGTTGAAAAATAGACCAACTTAATACGAGTTATATATCATTGAAAATTTCAAATCGTCTATTTTCTAATGATATAATTATTAGACTATACGGCTCAACTT
3. Beka GGTTGAAAAATAGACCAACTTAATACGAGTTATATATCATTGAAAATTTCAAATCGTCTATTTTCTAATGATATAATTATTAGACTATACGGCTCAACTT
4. Logan GGTTGAAAAATAGACCAACTTAATACGAGTTATATATCATTGAAAATTTCAAATCGTCTATTTTCTAATGATATAATTATTAGACTATACGGCTCAACTT
5. TR306 GGTTGAAAAATAGACCAACTTAATACGAGTTATATATCATTGAAAATTTCAAATCGTCTATTTTCTAATGATATAATTATTAGACTATACGGCTCAACTT
6. Harrington GGTTGAAAAATAGACCAACTTAATACGAGTTATATATCATTGAAAATTTCAAATCGTCTATTTTCTAATGATATAATTATTAGACTATACGGCTCAACTT
810 820 830 840 850 860 870 880 890 900
Identity
1. TR251 ATATTGACCAAATTATAGATCTAGAGATCTGCGCAAGCTTTATAAATCGGAAAGGAGATAGTATAGTAAGAATGATCTTGTTTTTGCGGGACAGCTTACG
2. CDC Bold ATATTGACCAAATTATAGATCTAGAGATCTGCGCAAGCTTTATAAATCGGAAAGGAGATAGTATAGTAAGAATGATCTTGTTTTTGCGGGACAGCTTACG
3. Beka ATATTGACCAAATTATAGATCTAGAGATCTGCGCAAGCTTTATAAATCGGAAAGGAGATAGTATAGTAAGAATGATCTTGTTTTTGCGGGACAGCTTACG
4. Logan ATATTGACCAAATTATAGATCTAGAGATCTGCGCAAGCTTTATAAATCGGAAAGGAGATAGTATAGTAAGAATGATCTTGTTTTTGCGGGACAGCTTACG
5. TR306 ATATTGACCAAATTATAGATCTAGAGATCTGCGCAAGCTTTATAAATCGGAAAGGAGATAGTATAGTAAGAATGATCTTGTTTTTGCGGGACAGCTTACG
6. Harrington ATATTGACCAAATTATAGATCTAGAGATCTGCGCAAGCTTTATAAATCGGAAAGGAGATAGTATAGTAAGAATGATCTTGTTTTTGCGGGACAGCTTACG

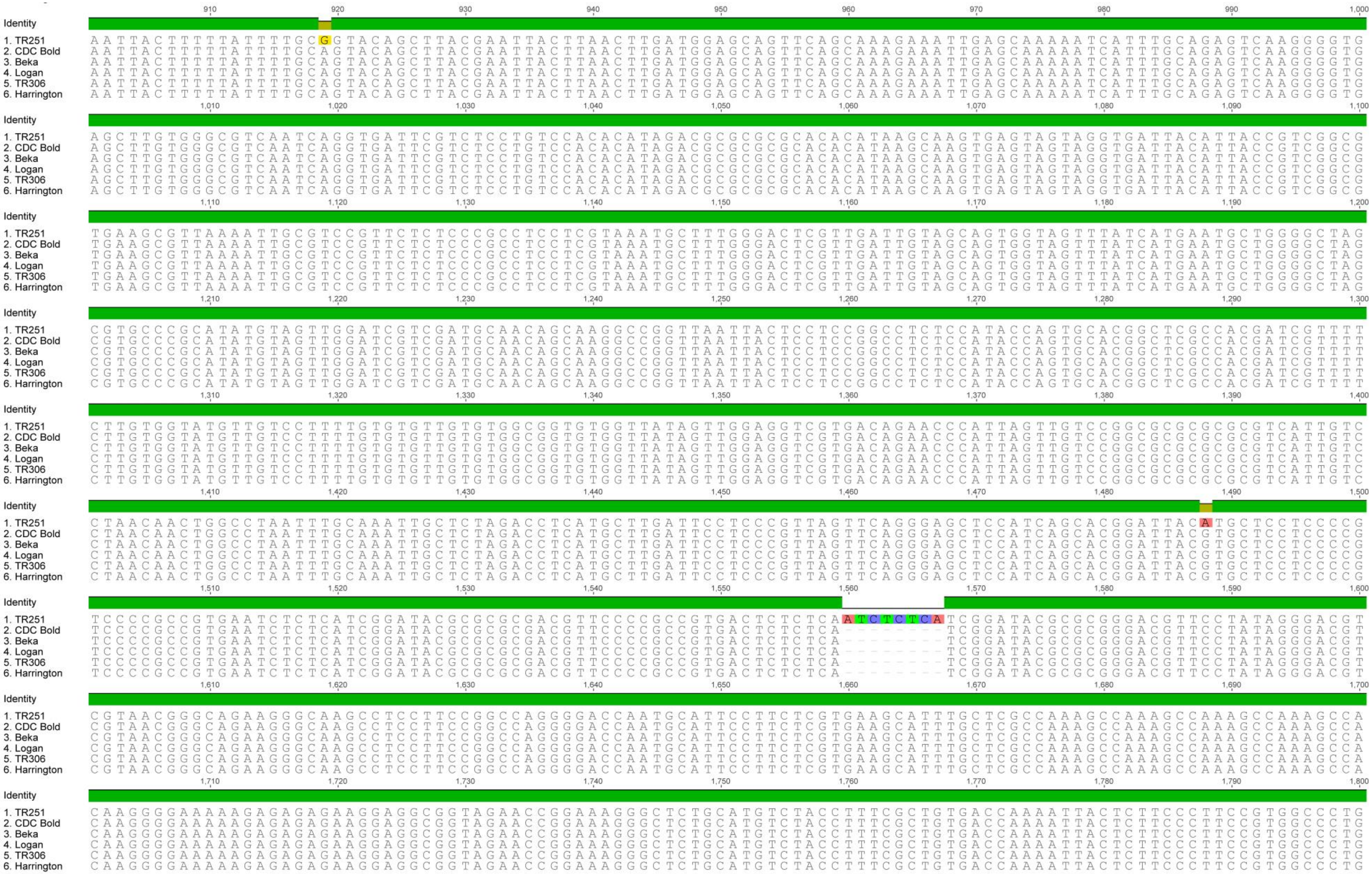
Identity
1. TR251
2. CDC Bold
3. Beka
4. Logan
5. TR306
6. Harrington
910 920 930 940 950 960 970 980 990 1,000
1,010 1,020 1,030 1,040 1,050 1,060 1,070 1,080 1,090 1,100
1,110 1,120 1,130 1,140 1,150 1,160 1,170 1,180 1,190 1,200
1,210 1,220 1,230 1,240 1,250 1,260 1,270 1,280 1,290 1,300
1,310 1,320 1,330 1,340 1,350 1,360 1,370 1,380 1,390 1,400
1,410 1,420 1,430 1,440 1,450 1,460 1,470 1,480 1,490 1,500
1,510 1,520 1,530 1,540 1,550 1,560 1,570 1,580 1,590 1,600
1,610 1,620 1,630 1,640 1,650 1,660 1,670 1,680 1,690 1,700
1,710 1,720 1,730 1,740 1,750 1,760 1,770 1,780 1,790 1,800

	1,810 – 1,900
Identity	
1. TR251	CTGCATGAGCATGGCCCTCCGCTTTTCAACCAGCAAGACAAGAATATGTCGCAATTGCTCCTACTAGTGTTAAACCGATGGATCAATCCTCCAAAATGCA
2. CDC Bold	CTGCATGAGCATGGCCCTCCGCTTTTCAACCAGCAAGACAAGAATATGTCGCAATTGCTCCTACTAGTGTTAAACCGATGGATCAATCCTCCAAAATGCA
3. Beka	CTGCATGAGCATGGCCCTCCGCTTTTCAACCAGCAAGACAAGAATATGTCGCAATTGCTCCTACTAGTGTTAAACCGATGGATCAATCCTCCAAAATGCA
4. Logan	CTGCATGAGCATGGCCCTCCGCTTTTCAACCAGCAAGACAAGAATATGTCGCAATTGCTCCTACTAGTGTTAAACCGATGGATCAATCCTCCAAAATGCA
5. TR306	CTGCATGAGCATGGCCCTCCGCTTTTCAACCAGCAAGACAAGAATATGTCGCAATTGCTCCTACTAGTGTTAAACCGATGGATCAATCCTCCAAAATGCA
6. Harrington	CTGCATGAGCATGGCCCTCCGCTTTTCAACCAGCAAGACAAGAATATGTCGCAATTGCTCCTACTAGTGTTAAACCGATGGATCAATCCTCCAAAATGCA

	1,910 – 2,000
Identity	
1. TR251	TCCCCCAACCAGACCCAACTTGCGCATAAACATCAATGTGCTAAAAATTCCATGGACAGAAGAGAGCGCCGCATCGCATTTGCCGGTGGGCTGCAAGGCC
2. CDC Bold	TCCCCCAACCAGACCCAACTTGCGCATAAACATCAATGTGCTAAAAATTCCATGGACAGAAGAGAGCGCCGCATCGCATTTGCCGGTGGGCTGCAAGGCC
3. Beka	TCCCCCAACCAGACCCAACTTGCGCATAAACATCAATGTGCTAAAAATTCCATGGACAGAAGAGAGCGCCGCATCGCATTTGCCGGTGGGCTGCAAGGCC
4. Logan	TCCCCCAACCAGACCCAACTTGCGCATAAACATCAATGTGCTAAAAATTCCATGGACAGAAGAGAGCGCCGCATCGCATTTGCCGGTGGGCTGCAAGGCC
5. TR306	TCCCCCAACCAGACCCAACTTGCGCATAAACATCAATGTGCTAAAAATTCCATGGACAGAAGAGAGCGCCGCATCGCATTTGCCGGTGGGCTGCAAGGCC
6. Harrington	TCCCCCAACCAGACCCAACTTGCGCATAAACATCAATGTGCTAAAAATTCCATGGACAGAAGAGAGCGCCGCATCGCATTTGCCGGTGGGCTGCAAGGCC

	2,010 – 2,100
Identity	
1. TR251	ACAGCGGAGCAAAGAGGCTTGTACGGTACTCAAACCAGCTCACTGATGCATGGCGATGCTACAGGGACTCACGGGCCACGCTGGCTGGGAGAGAGCAGCA
2. CDC Bold	ACAGCGGAGCAAAGAGGCTTGTACGGTACTCAAACCAGCTCACTGATGCATGGCGATGCTACAGGGACTCACGGGCCACGCTGGCTGGGAGAGAGCAGCA
3. Beka	ACAGCGGAGCAAAGAGGCTTGTACGGTACTCAAACCAGCTCACTGATGCATGGCGATGCTACAGGGACTCACGGGCCACGCTGGCTGGGAGAGAGCAGCA
4. Logan	ACAGCGGAGCAAAGAGGCTTGTACGGTACTCAAACCAGCTCACTGATGCATGGCGATGCTACAGGGACTCACGGGCCACGCTGGCTGGGAGAGAGCAGCA
5. TR306	ACAGCGGAGCAAAGAGGCTTGTACGGTACTCAAACCAGCTCACTGATGCATGGCGATGCTACAGGGACTCACGGGCCACGCTGGCTGGGAGAGAGCAGCA
6. Harrington	ACAGCGGAGCAAAGAGGCTTGTACGGTACTCAAACCAGCTCACTGATGCATGGCGATGCTACAGGGACTCACGGGCCACGCTGGCTGGGAGAGAGCAGCA

	2,110 – 2,200
Identity	
1. TR251	GAAAAGGCGCAAGAAAAGAATCTTTCTCTCAATCAAGGGCAACAACCAAGCACAAGCTCACAAACCCCCCACCAAGTTAAGAAAAAGAAAAAAAAATACA
2. CDC Bold	GAAAAGGCGCAAGAAAAGAATCTTTCTCTCAATCAAGGGCAACAACCAAGCACAAGCTCACAAACCCCCCACCAAGTTAAGAAAAAGAAAAAAAAATACA
3. Beka	GAAAAGGCGCAAGAAAAGAATCTTTCTCTCAATCAAGGGCAACAACCAAGCACAAGCTCACAAACCCCCCACCAAGTTAAGAAAAAGAAAAAAAAATACA
4. Logan	GAAAAGGCGCAAGAAAAGAATCTTTCTCTCAATCAAGGGCAACAACCAAGCACAAGCTCACAAACCCCCCACCAAGTTAAGAAAAAGAAAAAAAAATACA
5. TR306	GAAAAGGCGCAAGAAAAGAATCTTTCTCTCAATCAAGGGCAACAACCAAGCACAAGCTCACAAACCCCCCACCAAGTTAAGAAAAAGAAAAAAAAATACA
6. Harrington	GAAAAGGCGCAAGAAAAGAATCTTTCTCTCAATCAAGGGCAACAACCAAGCACAAGCTCACAAACCCCCCACCAAGTTAAGAAAAAGAAAAAAAAATACA

	2,210 – 2,300
Identity	
1. TR251	GGCACTTCGCATAACAAACAAACTAATCATCATCACCACTCATCGCCCCCGGCACCTTTATTCTACGCCCAGCTCCTCTCTCATTACACTTCTAACATAT
2. CDC Bold	GGCACTTCGCATAACAAACAAACTAATCATCATCACCACTCATCGCCCCCGGCACCTTTATTCTACGCCCAGCTCCTCTCTCATTACACTTCTAACATAT
3. Beka	GGCACTTCGCATAACAAACAAACTAATCATCATCACCACTCATCGCCCCCGGCACCTTTATTCTACGCCCAGCTCCTCTCTCATTACACTTCTAACATAT
4. Logan	GGCACTTCGCATAACAAACAAACTAATCATCATCACCACTCATCGCCCCCGGCACCTTTATTCTACGCCCAGCTCCTCTCTCATTACACTTCTAACATAT
5. TR306	GGCACTTCGCATAACAAACAAACTAATCATCATCACCACTCATCGCCCCCGGCACCTTTATTCTACGCCCAGCTCCTCTCTCATTACACTTCTAACATAT
6. Harrington	GGCACTTCGCATAACAAACAAACTAATCATCATCACCACTCATCGCCCCCGGCACCTTTATTCTACGCCCAGCTCCTCTCTCATTACACTTCTAACATAT

	2,310 – 2,400
Identity	
1. TR251	ATAACAATCAATCGGTGGAGGAGGAGGGACCAATTAATTAATGGATTAGTAATAACAATTCTTATGCGAGCAAAAATATACGTACTCCTGTACTAATAAG
2. CDC Bold	ATAACAATCAATCGGTGGAGGAGGAGGGACCAATTAATTAATGGATTAGTAATAACAATTCTTATGCGAGCAAAAATATACGTACTCCTGTACTAATAAG
3. Beka	ATAACAATCAATCGGTGGAGGAGGAGGGACCAATTAATTAATGGATTAGTAATAACAATTCTTATGCGAGCAAAAATATACGTACTCCTGTACTAATAAG
4. Logan	ATAACAATCAATCGGTGGAGGAGGAGGGACCAATTAATTAATGGATTAGTAATAACAATTCTTATGCGAGCAAAAATATACGTACTCCTGTACTAATAAG
5. TR306	ATAACAATCAATCGGTGGAGGAGGAGGGACCAATTAATTAATGGATTAGTAATAACAATTCTTATGCGAGCAAAAATATACGTACTCCTGTACTAATAAG
6. Harrington	ATAACAATCAATCGGTGGAGGAGGAGGGACCAATTAATTAATGGATTAGTAATAACAATTCTTATGCGAGCAAAAATATACGTACTCCTGTACTAATAAG

	2,410 – 2,500
Identity	
1. TR251	AATCGGAAATGCAAGCTAATCAGAGCTACTGCGTACGTACTAGTTTTCTTGCGCGGGGAGGGCATCACAAATCACATGGGACACGCAGGGGCAGGAATAT
2. CDC Bold	AATCGGAAATGCAAGCTAATCAGAGCTACTGCGTACGTACTAGTTTTCTTGCGCGGGGAGGGCATCACAAATCACATGGGACACGCAGGGGCAGGAATAT
3. Beka	AATCGGAAATGCAAGCTAATCAGAGCTACTGCGTACGTACTAGTTTTCTTGCGCGGGGAGGGCATCACAAATCACATGGGACACGCAGGGGCAGGAATAT
4. Logan	AATCGGAAATGCAAGCTAATCAGAGCTACTGCGTACGTACTAGTTTTCTTGCGCGGGGAGGGCATCACAAATCACATGGGACACGCAGGGGCAGGAATAT
5. TR306	AATCGGAAATGCAAGCTAATCAGAGCTACTGCGTACGTACTAGTTTTCTTGCGCGGGGAGGGCATCACAAATCACATGGGACACGCAGGGGCAGGAATAT
6. Harrington	AATCGGAAATGCAAGCTAATCAGAGCTACTGCGTACGTACTAGTTTTCTTGCGCGGGGAGGGCATCACAAATCACATGGGACACGCAGGGGCAGGAATAT

	2,510 – 2,600
Identity	
1. TR251	GAGTGGAAGCAACCTGGTAGGTAGGTGAGGTGCGGTGCGGTGCGGGCATATGATAAGCATATGGGCTGGGCCGAGCATACACGCGTACATGCATTGCATT
2. CDC Bold	GAGTGGAAGCAACCTGGTAGGTAGGTGAGGTGCGGTGCGGTGCGGGCATATGATAAGCATATGGGCTGGGCCGAGCATACACGCGTACATGCATTGCATT
3. Beka	GAGTGGAAGCAACCTGGTAGGTAGGTGAGGTGCGGTGCGGTGCGGGCATATGATAAGCATATGGGCTGGGCCGAGCATACACGCGTACATGCATTGCATT
4. Logan	GAGTGGAAGCAACCTGGTAGGTAGGTGAGGTGCGGTGCGGTGCGGGCATATGATAAGCATATGGGCTGGGCCGAGCATACACGCGTACATGCATTGCATT
5. TR306	GAGTGGAAGCAACCTGGTAGGTAGGTGAGGTGCGGTGCGGTGCGGGCATATGATAAGCATATGGGCTGGGCCGAGCATACACGCGTACATGCATTGCATT
6. Harrington	GAGTGGAAGCAACCTGGTAGGTAGGTGAGGTGCGGTGCGGTGCGGGCATATGATAAGCATATGGGCTGGGCCGAGCATACACGCGTACATGCATTGCATT

	2,610 – 2,700
Identity	
1. TR251	TGCATACACCGAAGGAAGTGCTTGGCACCACCTACATATAAACGCACCCGCACACCGCTCAAACACAATTTACAACCCGCACACAGCTCCAACCCGTTGG
2. CDC Bold	TGCATACACCGAAGGAAGTGCTTGGCACCACCTACATATAAACGCACCCGCACACCGCTCAAACACAATTTACAACCCGCACACAGCTCCAACCCGTTGG
3. Beka	TGCATACACCGAAGGAAGTGCTTGGCACCACCTACATATAAACGCACCCGCACACCGCTCAAACACAATTTACAACCCGCACACAGCTCCAACCCGTTGG
4. Logan	TGCATACACCGAAGGAAGTGCTTGGCACCACCTACATATAAACGCACCCGCACACCGCTCAAACACAATTTACAACCCGCACACAGCTCCAACCCGTTGG
5. TR306	TGCATACACCGAAGGAAGTGCTTGGCACCACCTACATATAAACGCACCCGCACACCGCTCAAACACAATTTACAACCCGCACACAGCTCCAACCCGTTGG
6. Harrington	TGCATACACCGAAGGAAGTGCTTGGCACCACCTACATATAAACGCACCCGCACACCGCTCAAACACAATTTACAACCCGCACACAGCTCCAACCCGTTGG

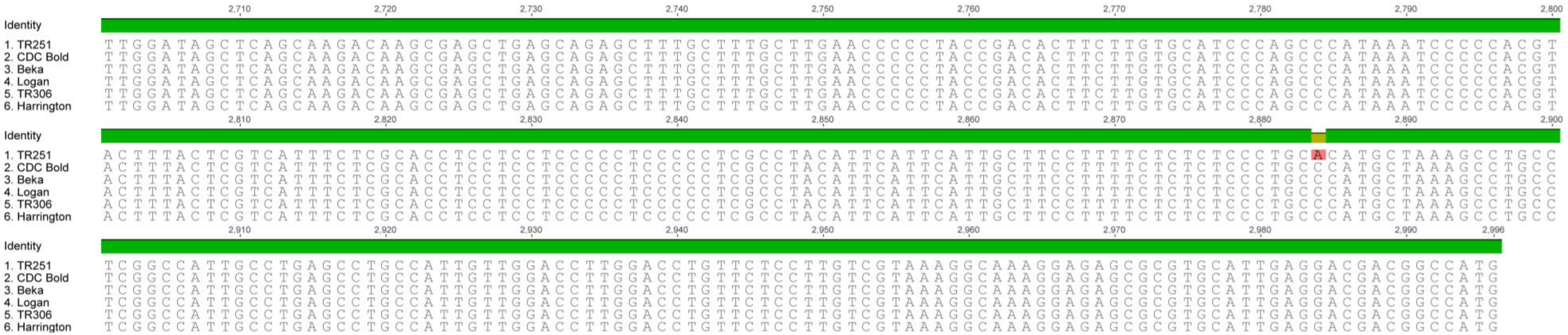

Fig. S3 Alignment of sequences from TR251, CDC Bold, Beka, Logan, TR306 and Harrington for a putative promoter region of *HvCslF6*.
