# Supplementary material for: Differential expression of the HvCslF6 gene late in grain development may explain quantitative differences in (1,3;1,4)-β-glucan concentration in barley
Source: Mol Breed. 2015 Jan 20;35(1):20. doi: 10.1007/s11032-015-0208-6 (PMC4298655; doi:10.1007/s11032-015-0208-6)
Supplement: Supplementary file 4 — KASP™ marker assays designed to detect six DNA polymorphisms within the HvCslF6 gene of barley (PDF 118 kb) [file 11032_2015_208_MOESM4_ESM.pdf]

**Article**	Differential expression of the *HvCslF6* gene late in grain development may explain quantitative differences in (1,3;1,4)-β-glucan concentration in barley
**Journal**	Molecular Breeding
**Authors**	Sie Chuong Wong, Rachel A Burton, Neil J Shirley, Alan Little, Julian Schwerdt, Kelvin H.P. Khoo, Geoffrey B Fincher and Diane E. Mather
**Corresponding author**	Diane E. Mather (diane.mather@adelaide.edu.au) School of Agriculture, Food and Wine and ARC Centre of Excellence in Plant Cell Walls, Waite Research Institute, University of Adelaide

Online Resource 4 KASP™ marker assays designed to detect six DNA polymorphisms within the *HvCslF6* gene of barley

Table S4 KASP™ marker assays designed to detect six DNA polymorphisms within the *HvCslF6* gene of barley

Marker	Polymorphism[a]	Region	Primers	Primer sequences
wri51	558 (SNP4)	Intron 1	wri51_F1	GAAGGTGACCAAGTTCATGCTGGTCGGTGTTAATCAGAGTAATTATTG
			wri51_F2	GAAGGTCGGAGTCAACGGATTAGGTCGGTGTTAATCAGAGTAATTATTA
			wri51_R	GTGCACCTGTCTCGGATTACTCAAA
wri52	1137-1150 (indel2)	Intron 1	wri52_F1	GAAGGTGACCAAGTTCATGCTAAAAGAGAAAAATATTATCATGTCATGTCATGA
			wri52_F2	GAAGGTCGGAGTCAACGGATTAGAGAAAAATATTATCATGTCATGTCATGC
			wri52_R	ATTCGAGCGGTGGCAACACTTCTTT
wri53	1398 (SNP12)	Intron 1	wri53_F1	GAAGGTGACCAAGTTCATGCTTCTACCGCTAATCATTACTACTTTC
			wri53_F2	GAAGGTCGGAGTCAACGGATTCCTTCTACCGCTAATCATTACTACTTTT
			wri53_R	GTGTACTCTCTGTATCTAAATAATTATAAT
wri54	1656 (SNP14)	Intron 1	wri54_F1	GAAGGTGACCAAGTTCATGCTGCCAAGTGTGCCAGCC
			wri54-F2	GAAGGTCGGAGTCAACGGATTTGCTGCCAAGTGTGCCAGCT
			wri54_R	CAAGGAGAGAGCTGGCAAGTCAAT
wri55	3262 (SNP 21)	Intron 2	wri55_F1	GAAGGTGACCAAGTTCATGCTGAGTAGGAGTAATGCAGATTTACTATTG
			wri55_F2	GAAGGTCGGAGTCAACGGATTGGAGTAGGAGTAATGCAGATTTACTATTA
			wri55_F3	CAAGGTTCTGTAATTGTGCTTCCTTCTTT
wri56	4064 (SNP23)[b]	Exon 3	wri56_F1	GAAGGTGACCAAGTTCATGCTCCCTCAGCCGCCGCGGT
			wri56_F2	GAAGGTCGGAGTCAACGGATTCCTCAGCCGCCGCGGC
			wri56_R	CGTCGCACCCGTCGCCCTA

[a] Nucleotide position and SNP or indel designation from Taketa et al. (2011)

[b] This SNP causes an alanine-threonine substitution at position 590 of the translated protein
