# Supplementary material for: Differential expression of the HvCslF6 gene late in grain development may explain quantitative differences in (1,3;1,4)-β-glucan concentration in barley
Source: Mol Breed. 2015 Jan 20;35(1):20. doi: 10.1007/s11032-015-0208-6 (PMC4298655; doi:10.1007/s11032-015-0208-6)
Supplement: Supplementary file 5 — Alignment of sequences for eight alleles of HvCslF6, showing the positions of polymorphisms for which marker assays were developed (PDF 245 kb) [file 11032_2015_208_MOESM5_ESM.pdf]

**Article**	Differential expression of the *HvCslF6* gene late in grain development may explain quantitative differences in (1,3;1,4)-β-glucan concentration in barley
**Journal**	Molecular Breeding
**Authors**	Sie Chuong Wong, Rachel A Burton, Neil J Shirley, Alan Little, Julian Schwerdt, Kelvin H.P. Khoo, Geoffrey B Fincher and Diane E. Mather
**Corresponding author**	Diane E. Mather (diane.mather@adelaide.edu.au) School of Agriculture, Food and Wine and ARC Centre of Excellence in Plant Cell Walls, Waite Research Institute, University of Adelaide

Online Resource 5 Alignment of sequences for eight alleles of *HvCslF6*, showing the positions of polymorphisms for which marker assays were developed

```
                              1        10        20        30        40        50        60
                              |         |         |         |         |         |         |
        AB621318.1 - TR251    ATGGCGCCAGCGGTGGCCGGAGGGGGCCGCGTGCGGAGCAATGAGCCGGTTGCTGCTGCT
    AB621312.1 - CDC Fibar    ATGGCGCCAGCGGTGGCCGGAGGGGGCCGCGTGCGGAGCAATGAGCCGGTTGCTGCTGCT
        AB621313.1 - Azhul    ATGGCGCCAGCGGTGGCCGGAGGGGGCCGCGTGCGGAGCAATGAGCCGGTTGCTGCTGCT
  AB621325.1 - B83-12/21/3    ATGGCGCCAGCGGTGGCCGGAGGGGGCCGCGTGCGGAGCAATGAGCCGGTTGCTGCTGCT
         AB621322.1 - Beka    ATGGCGCCAGCGGTGGCCGGAGGGGGCCGCGTGCGGAGCAATGAGCCGGTTGCTGCTGCT
     AB621321.1 - CDC Bold    ATGGCGCCAGCGGTGGCCGGAGGGGGCCGCGTGCGGAGCAATGAGCCGGTTGCTGCTGCT
        AB621332.1 - Morex    ATGGCGCCAGCGGTGGCCGGAGGGGGCCGCGTGCGGAGCAATGAGCCGGTTGCTGCTGCT
 AB621319.1 - Nishinohoshi    ATGGCGCCAGCGGTGGCCGGAGGGGGCCGCGTGCGGAGCAATGAGCCGGTTGCTGCTGCT

        AB621318.1 - TR251    GCCGCCGCGCCGGCGGCCAGCGGCAAGCCCTGCGTGTGCGGCTTCCAGGTTTGCGCCTGC
    AB621312.1 - CDC Fibar    GCCGCCGCGCCGGCGGCCAGCGGCAAGCCCTGCGTGTGCGGCTTCCAGGTTTGCGCCTGC
        AB621313.1 - Azhul    GCCGCCGCGCCGGCGGCCAGCGGCAAGCCCTGCGTGTGCGGCTTCCAGGTTTGCGCCTGC
  AB621325.1 - B83-12/21/3    GCCGCCGCGCCGGCGGCCAGCGGCAAGCCCTGCGTGTGCGGCTTCCAGGTTTGCGCCTGC
         AB621322.1 - Beka    GCCGCCGCGCCGGCGGCCAGCGGCAAGCCCTGCGTGTGCGGCTTCCAGGTTTGCGCCTGC
     AB621321.1 - CDC Bold    GCCGCCGCGCCGGCGGCCAGCGGCAAGCCCTGCGTGTGCGGCTTCCAGGTTTGCGCCTGC
        AB621332.1 - Morex    GCCGCCGCGCCGGCGGCCAGCGGCAAGCCCTGCGTGTGCGGCTTCCAGGTTTGCGCCTGC
 AB621319.1 - Nishinohoshi    GCCGCCGCGCCGGCGGCCAGCGGCAAGCCCTGCGTGTGCGGCTTCCAGGTTTGCGCCTGC

        AB621318.1 - TR251    ACGGGGTCGGCCGCGGTGGCCTCCGCCGCCTCGTCGCTGGACATGGACATCGTAGCCATG
    AB621312.1 - CDC Fibar    ACGGGGTCGGCCGCGGTGGCCTCCGCCGCCTCGTCGCTGGACATGGACATCGTGGCCATG
        AB621313.1 - Azhul    ACGGGGTCGGCCGCGGTGGCCTCCGCCGCCTCGTCGCTGGACATGGACATCGTGGCCATG
  AB621325.1 - B83-12/21/3    ACGGGGTCGGCCGCGGTGGCCTCCGCCGCCTCGTCGCTGGACATGGACATCGTGGCCATG
         AB621322.1 - Beka    ACGGGGTCGGCCGCGGTGGCCTCCGCCGCCTCGTCGCTGGACATGGACATCGTGGCCATG
     AB621321.1 - CDC Bold    ACGGGGTCGGCCGCGGTGGCCTCCGCCGCCTCGTCGCTGGACATGGACATCGTGGCCATG
        AB621332.1 - Morex    ACGGGGTCGGCCGCGGTGGCCTCCGCCGCCTCGTCGCTGGACATGGACATCGTGGCCATG
 AB621319.1 - Nishinohoshi    ACGGGGTCGGCCGCGGTGGCCTCCGCCGCCTCGTCGCTGGACATGGACATCGTGGCCATG

        AB621318.1 - TR251    GGGCAGATCGGCGCCGTCAACGACGAGAGCTGGGTGGGCGTGGAGCTCGGCGAAGATGGC
    AB621312.1 - CDC Fibar    GGGCAGATCGGCGCCGTCAACGACGAGAGCTGGGTGGGCGTGGAGCTCGGCGAAGATGGC
        AB621313.1 - Azhul    GGGCAGATCGGCGCCGTCAACGACGAGAGCTGGGTGGGCGTGGAGCTCGGCGAAGATGGC
  AB621325.1 - B83-12/21/3    GGGCAGATCGGCGCCGTCAACGACGAGAGCTGGGTGGGCGTGGAGCTCGGCGAAGATGGC
         AB621322.1 - Beka    GGGCAGATCGGCGCCGTCAACGACGAGAGCTGGGTGGGCGTGGAGCTCGGCGAAGATGGC
     AB621321.1 - CDC Bold    GGGCAGATCGGCGCCGTCAACGACGAGAGCTGGGTGGGCGTGGAGCTCGGCGAAGATGGC
        AB621332.1 - Morex    GGGCAGATCGGCGCCGTCAACGACGAGAGCTGGGTGGGCGTGGAGCTCGGCGAAGATGGC
 AB621319.1 - Nishinohoshi    GGGCAGATCGGCGCCGTCAACGACGAGAGCTGGGTGGGCGTGGAGCTCGGCGAAGATGGC

        AB621318.1 - TR251    GAGACCGACGAAAGCGGTGCCGCCGTTGACGACCGCCCCGTATTCCGCACCGAGAAGATC
    AB621312.1 - CDC Fibar    GAGACCGACGAAAGCGGTGCCGCCGTTGACGACCGCCCCGTATTCCGCACCGAGAAGATC
        AB621313.1 - Azhul    GAGACCGACGAAAGCGGTGCCGCCGTTGACGACCGCCCCGTATTCCGCACCGAGAAGATC
  AB621325.1 - B83-12/21/3    GAGACCGACGAAAGCGGTGCCGCCGTTGACGACCGCCCCGTATTCCGCACCGAGAAGATC
         AB621322.1 - Beka    GAGACCGACGAAAGCGGTGCCGCCGTTGACGACCGCCCCGTATTCCGCACCGAGAAGATC
     AB621321.1 - CDC Bold    GAGACCGACGAAAGCGGTGCCGCCGTTGACGACCGCCCCGTATTCCGCACCGAGAAGATC
        AB621332.1 - Morex    GAGACCGACGAAAGCGGTGCCGCCGTTGACGACCGCCCCGTATTCCGCACCGAGAAGATC
 AB621319.1 - Nishinohoshi    GAGACCGACGAAAGCGGTGCCGCCGTTGACGACCGCCCCGTATTCCGCACCGAGAAGATC

        AB621318.1 - TR251    AAGGGTGTCCTCCTCCACCCCTACCGGTACGTCCTGCTCCCACAACTAAACAGAAACTCC
    AB621312.1 - CDC Fibar    AAGGGTGTCCTCCTCCACCCCTACCGGTACGTCCTGCTCCCACAACTAAACAGAAACTCC
        AB621313.1 - Azhul    AAGGGTGTCCTCCTCCACCCCTACCGGTACGTCCTGCTCCCACAACTAAACAGAAACTCC
  AB621325.1 - B83-12/21/3    AAGGGTGTCCTCCTCCACCCCTACCGGTACGTCCTGCTCCCACAACTAAACAGAAACTCC
         AB621322.1 - Beka    AAGGGTGTCCTCCTCCACCCCTACCGGTACGTCCTGCTCCCACAACTAAACAGAAACTCC
     AB621321.1 - CDC Bold    AAGGGTGTCCTCCTCCACCCCTACCGGTACGTCCTGCTCCCACAACTAAACAGAAACTCC
        AB621332.1 - Morex    AAGGGTGTCCTCCTCCACCCCTACCGGTACGTCCTGCTCCCACAACTAAACAGAAACTCC
 AB621319.1 - Nishinohoshi    AAGGGTGTCCTCCTCCACCCCTACCGGTACGTTCTGCTCCCACAACTAAACAGAAACTCC

        AB621318.1 - TR251    CTATATCTGCGTCACACTCAACAATTAATCCAACTAAGTCTCTCTACTACTATAGTATTT
    AB621312.1 - CDC Fibar    CTATATCTGCGTCACACTCAACAATTAATCCAACTAAGTCTCTCTACTACTCTAGTATTT
        AB621313.1 - Azhul    CTATATCTGCGTCACACTCAACAATTAATCCAACTAAGTCTCTCTACTACTCTAGTATTT
  AB621325.1 - B83-12/21/3    CTATATCTGCGTCACACTCAACAATTAATCCAACTAAGTCTCTCTACTACTCTAGTATTT
         AB621322.1 - Beka    CTATATCTGCGTCACACTCAACAATTAATCCAACTAAGTCTCTCTACTACTCTAGTATTT
     AB621321.1 - CDC Bold    CTATATCTGCGTCACACTCAACAATTAATCCAACTAAGTCTCTCTACTACTCTAGTATTT
        AB621332.1 - Morex    CTATATCTGCGTCACACTCAACAATTAATCCAACTAAGTCTCTCTACTACTCTAGTATTT
 AB621319.1 - Nishinohoshi    CTATATCTGCGTCACACTCAACAATTAATCCAACTAAGTCTCTCTACTACTCTAGTATTT

        AB621318.1 - TR251    ATTTTTACTCTCTATCTGCACAACAAGCGCTACTACAATTAACCCAACAAGCACCACGCC
    AB621312.1 - CDC Fibar    ATTTTTACTCTCTATCTGCACAACAAGCGCTACTACAATTAACCCAACAAGCACCACGCC
        AB621313.1 - Azhul    ATTTTTACTCTCTATCTGCACAACAAGCGCTACTACAATTAACCCAACAAGCACCACGCC
  AB621325.1 - B83-12/21/3    ATTTTTACTCTCTATCTGCACAACAAGCGCTACTACAATTAACCCAACAAGCACCACGCC
         AB621322.1 - Beka    ATTTTTACTCTCTATCTGCACAACAAGCGCTACTACAATTAACCCAACAAGCACCACGCC
     AB621321.1 - CDC Bold    ATTTTTACTCTCTATCTGCACAACAAGCGCTACTACAATTAACCCAACAAGCACCACGCC
        AB621332.1 - Morex    ATTTTTACTCTCTATCTGCACAACAAGCGCTACTACAATTAACCCAACAAGCACCACGCC
 AB621319.1 - Nishinohoshi    ATTTTTACTCTCTATCTGCACAACAAGCGCTACTACAATTAACCCAACAAGCACCACGCC

        AB621318.1 - TR251    AGGTTGACAGTCAGGATAATTTGATCTTGACCGGAGTAAGTACTAGTACTAGGTCGGTGT
    AB621312.1 - CDC Fibar    AGGTTGACAGTCAGGATAATTTGATCTTGACCGGAGTAAGTACTAGTACTAGGTCGGTGT
        AB621313.1 - Azhul    AGGTTGACAGTCAGGATAATTTGATCTTGACCGGAGTAAGTACTAGTACTAGGTCGGTGT
  AB621325.1 - B83-12/21/3    AGGTTGACAGTCAGGATAATTTGATCTTGACCGGAGTAAGTACTAGTACTAGGTCGGTGT
         AB621322.1 - Beka    AGGTTGACAGTCAGGATAATTTGATCTTGACCGGAGTAAGTACTAGTACTAGGTCGGTGT
     AB621321.1 - CDC Bold    AGGTTGACAGTCAGGATAATTTGATCTTGACCGGAGTAAGTACTAGTACTAGGTCGGTGT
        AB621332.1 - Morex    AGGTTGACAGTCAGGATAATTTGATCTTGACCGGAGTAAGTACTAGTACTAGGTCGGTGT
 AB621319.1 - Nishinohoshi    AGGTTGACAGTCAGGATAATTTGATCTTGACCGGAGTAAGTACTAGTACTAGGTCGGTGT
```

```
                                        SNP4
      AB621318.1 - TR251    TAATCAGAGTAATTATTGCACTAGTTAATTAAAATTTGAGTAATCCGAGACAGGTGCACG
  AB621312.1 - CDC Fibar    TAATCAGAGTAATTATTACACTAGTTAATTAAAATTTGAGTAATCCGAGACAGGTGCACG
      AB621313.1 - Azhul    TAATCAGAGTAATTATTACACTAGTTAATTAAAATTTGAGTAATCCGAGACAGGTGCACG
AB621325.1 - B83-12/21/3    TAATCAGAGTAATTATTACACTAGTTAATTAAAATTTGAGTAATCCGAGACAGGTGCACG
       AB621322.1 - Beka    TAATCAGAGTAATTATTACACTAGTTAATTAAAATTTGAGTAATCCGAGACAGGTGCACG
   AB621321.1 - CDC Bold    TAATCAGAGTAATTATTGCACTAGTTAATTAAAATTTGAGTAATCCGAGACAGGTGCACG
      AB621332.1 - Morex    TAATCAGAGTAATTATTGCACTAGTTAATTAAAATTTGAGTAATCCGAGACAGGTGCACG
AB621319.1 - Nishinohoshi    TAATCAGAGTAATTATTGCACTAGTTAATTAAAATTTGAGTAATCCGAGACAGGTGCACG

      AB621318.1 - TR251    TTAGGGCCGGGCCAATGATCGCTCGAATCCACCCAAAATAGCGCGTCCCGGTGTGGGCTG
  AB621312.1 - CDC Fibar    TTAGGGCCGGGCCAATGATGGCTCGAATCCACCCAAAATAGCGCGTCCCGGTGTGGGCTG
      AB621313.1 - Azhul    TTAGGGCCGGGCCAATGATGGCTCGAATCCACCCAAAATAGCGCGTCCCGGTGTGGGCTG
AB621325.1 - B83-12/21/3    TTAGGGCCGGGCCAATGATGGCTCGAATCCACCCAAAATAGCGCGTCCCGGTGTGGGCTG
       AB621322.1 - Beka    TTAGGGCCGGGCCAATGATGGCTCGAATCCACCCAAAATAGCGCGTCCCGGTGTGGGCTG
   AB621321.1 - CDC Bold    TTAGGGCCGGGCCAATGATGGCTCGAATCCACCCAAAATAGCGCGTCCCGGTGTGGGCTG
      AB621332.1 - Morex    TTAGGGCCGGGCCAATGATGGCTCGAATCCACCCAAAATAGCGCGTCCCGGTGTGGGCTG
AB621319.1 - Nishinohoshi    TTAGGGCCGGGCCAATGATGGCTCGAATCCACCCAAAATAGCGCGTCCCGGTGTGGGCTG

      AB621318.1 - TR251    TCGGCTCGGTGCTTCTTCCTTCCATTTTACTAGTCGCAGTCACTGCAGCTTGGGCCCACG
  AB621312.1 - CDC Fibar    TCGGCTCGGTGCTTCTTCCTTCCATTTTACTAGTCGCAGTCACTGCAGCTTGGGCCCACG
      AB621313.1 - Azhul    TCGGCTCGGTGCTTCTTCCTTCCATTTTACTAGTCGCAGTCACTGCAGCTTGGGCCCACG
AB621325.1 - B83-12/21/3    TCGGCTCGGTGCTTCTTCCTTCCATTTTACTAGTCGCAGTCACTGCAGCTTGGGCCCACG
       AB621322.1 - Beka    TCGGCTCGGTGCTTCTTCCTTCCATTTTACTAGTCGCAGTCACTGCAGCTTGGGCCCACG
   AB621321.1 - CDC Bold    TCGGCTCGGTGCTTCTTCCTTCCATTTTACTAGTCGCAGTCACTGCAGCTTGGGCCCACG
      AB621332.1 - Morex    TCGGCTCGGTGCTTCTTCCTTCCATTTTACTAGTCGCAGTCACTGCAGCTTGGGCCCACG
AB621319.1 - Nishinohoshi    TCGGCTCGGTGCTTCTTCCTTCCATTTTACTAGTCGCAGTCACTGCAGCTTGGGCCCACG

      AB621318.1 - TR251    GGAGGGGACGTTAGCCGTTGGGCCTGCCTGGCAGGTGGGCCCCGGTGGCCACCCTGGCGG
  AB621312.1 - CDC Fibar    GGAGGGGACGTTAGCCGTTGGGCCTGCCTGGCAGGTGGGCCCCGGTGGCCACCCTGGCGG
      AB621313.1 - Azhul    GGAGGGGACGTTAGCCGTTGGGCCTGCCTGGCAGGTGGGCCCCGGTGGCCACCCTGGCGG
AB621325.1 - B83-12/21/3    GGAGGGGACGTTAGCCGTTGGGCCTGCCTGGCAGGTGGGCCCCGGTGGCCACCCTGGCGG
       AB621322.1 - Beka    GGAGGGGACGTTAGCCGTTGGGCCTGCCTGGCAGGTGGGCCCCGGTGGCCACCCTGGCGG
   AB621321.1 - CDC Bold    GGAGGGGACGTTAGCCGTTGGGCCTGCCTGGCAGGTGGGCCCCGGTGGCCACCCTGGCGG
      AB621332.1 - Morex    GGAGGGGACGTTAGCCGTTGGGCCTGCCTGGCAGGTGGGCCCCGGTGGCCACCCTGGCGG
AB621319.1 - Nishinohoshi    GGAGGGGACGTTAGCCGTTGGGCCTGCCTGGCAGGTGGGCCCCGGTGGCCACCCTGGCGG

      AB621318.1 - TR251    CTCATAAATCCTTGCTACTTTGGAGCTGTAATGGACGCTCTGCAATAGCAATAGGAATCC
  AB621312.1 - CDC Fibar    CTCATAAATCCTTGCTACTTTGGAGCTGTAATGGACGCTCTGCAATAGCAATAGGAATCC
      AB621313.1 - Azhul    CTCATAAATCCTTGCTACTTTGGAGCTGTAATGGACGCTCTGCAATAGCAATAGGAATCC
AB621325.1 - B83-12/21/3    CTCATAAATCCTTGCTACTTTGGAGCTGTAATGGACGCTCTGCAATAGCAATAGGAATCC
       AB621322.1 - Beka    CTCATAAATCCTTGCTACTTTGGAGCTGTAATGGACGCTCTGCAATAGCAATAGGAATCC
   AB621321.1 - CDC Bold    CTCATAAATCCTTGCTACTTTGGAGCTGTAATGGACGCTCTGCAATAGCAATAGGAATCC
      AB621332.1 - Morex    CTCATAAATCCTTGCTACTTTGGAGCTGTAATGGACGCTCTGCAATAGCAATAGGAATCC
AB621319.1 - Nishinohoshi    CTCATAAATCCTTGCTACTTTGGAGCTGTAATGGACGCTCTGCAATAGCAATAGGAATCC

      AB621318.1 - TR251    GAGGTGAAACGACGACAGTGGGCATGGCATGGCTTGCATGTGAATCCAAGCCACATCATT
  AB621312.1 - CDC Fibar    GAGGTGAAACGACGACAGTGGGCATGGCATGGCTTGCATGTGAATCCAAGCCACATCATT
      AB621313.1 - Azhul    GAGGTGAAACGACGACAGTGGGCATGGCATGGCTTGCATGTGAATCCAAGCCACATCATT
AB621325.1 - B83-12/21/3    GAGGTGAAACGACGACAGTGGGCATGGCATGGCTTGCATGTGAATCCAAGCCACATCATT
       AB621322.1 - Beka    GAGGTGAAACGACGACAGTGGGCATGGCATGGCTTGCATGTGAATCCAAGCCACATCATT
   AB621321.1 - CDC Bold    GAGGTGAAACGACGACAGTGGGCATGGCATGGCTTGCATGTGAATCCAAGCCACATCATT
      AB621332.1 - Morex    GAGGTGAAACGACGACAGTGGGCATGGCATGGCTTGCATGTGAATCCAAGCCACATCATT
AB621319.1 - Nishinohoshi    GAGGTGAAACGACGACAGTGGGCATGGCATGGCTTGCATGTGAATCCAAGCCACATCATT

      AB621318.1 - TR251    AAAAGCATCCTCCCTGGGCACGTCGCGGTGAGAAAGTTGGATAAACTTTTGGGGGTTCGG
  AB621312.1 - CDC Fibar    AAAAGCATCCTCCCTGGGCACGTCGCGGTGAGAAAGTTGGATAAACTTTTGGGGGTTCGG
      AB621313.1 - Azhul    AAAAGCATCCTCCCTGGGCACGTCGCGGTGAGAAAGTTGGATAAACTTTTGGGGGTTCGG
AB621325.1 - B83-12/21/3    AAAAGCATCCTCCCTGGGCACGTCGCGGTGAGAAAGTTGGATAAACTTTTGGGGGTTCGG
       AB621322.1 - Beka    AAAAGCATCCTCCCTGGGCACGTCGCGGTGAGAAAGTTGGATAAACTTTTGGGGGTTCGG
   AB621321.1 - CDC Bold    AAAAGCATCCTCCCTGGGCACGTCGCGGTGAGAAAGTTGGATAAACTTTTGGGGGTTCGG
      AB621332.1 - Morex    AAAAGCATCCTCCCTGGGCACGTCGCGGTGAGAAAGTTGGATAAACTTTTGGGGGTTCGG
AB621319.1 - Nishinohoshi    AAAAGCATCCTCCCTGGGCACGTCGCGGTGAGAAAGTTGGATAAACTTTTGGGGGTTCGG

      AB621318.1 - TR251    ACAAGATGAGAAAAAGCAAGTAACATGGCCCTTTTTTGGCACCGAAGAAATCCTATGTAC
  AB621312.1 - CDC Fibar    ACAAGATGAGAAAAAGCAAGTAACATGTCCCTTTTTTGGCACCGAAGAAATCCTATGTAC
      AB621313.1 - Azhul    ACAAGATGAGAAAAAGCAAGTAACATGTCCCTTTTTTGGCACCGAAGAAATCCTATGTAC
AB621325.1 - B83-12/21/3    ACAAGATGAGAAAAAGCAAGTAACATGTCCCTTTTTTGGCACCGAAGAAATCCTATGTAC
       AB621322.1 - Beka    ACAAGATGAGAAAAAGCAAGTAACATGTCCCTTTTTTGGCACCGAAGAAATCCTATGTAC
   AB621321.1 - CDC Bold    ACAAGATGAGAAAAAGCAAGTAACATGTCCCTTTTTTGGCACCGAAGAAATCCTATGTAC
      AB621332.1 - Morex    ACAAGATGAGAAAAAGCAAGTAACATGTCCCTTTTTTGGCACCGAAGAAATCCTATGTAC
AB621319.1 - Nishinohoshi    ACAAGATGAGAAAAAGCAAGTAACATGTCCCTTTTTTGGCACCGAAGAAATCCTATGTAC

      AB621318.1 - TR251    GGCGAGTTTTTTCTGCATCTAGTTATGGGTAGATGTACGTTAGTTTTTGTTGAGCGTTTT
  AB621312.1 - CDC Fibar    GGCGAGTTTTTTCTGCATCTAGTTATGGGTAGATGTACGTTAGCTTTTGTTGAGCGTTTT
      AB621313.1 - Azhul    GGCGAGTTTTTTCTGCATCTAGTTATGGGTAGATGTACGTTAGCTTTTGTTGAGCGTTTT
AB621325.1 - B83-12/21/3    GGCGAGTTTTTTCTGCATCTAGTTATGGGTAGATGTACGTTAGCTTTTGTTGAGCGTTTT
       AB621322.1 - Beka    GGCGAGTTTTTTCTGCATCTAGTTATGGGTAGATGTACGTTAGCTTTTGTTGAGCGTTTT
   AB621321.1 - CDC Bold    GGCGAGTTTTTTCTGCATCTAGTTATGGGTAGATGTACGTTAGTTTTTGTTGAGCGTTTT
      AB621332.1 - Morex    GGCGAGTTTTTTCTGCATCTAGTTATGGGTAGATGTACGTTAGTTTTTGTTGAGCGTTTT
AB621319.1 - Nishinohoshi    GGCGAGTTTTTTCTGCATCTAGTTATGGGTAGATGTACGTTAGTTTTTGTTGAGCGTTTT
```

```
      AB621318.1 - TR251    ATGTGCTACATATATGGAGAAAAAGAGAAAAATATTATCATGTCATGTCATGCCATG---
  AB621312.1 - CDC Fibar    ATGTGCTACATATATGGAGAAAAAGAGAAAAATATTATCATGTCATGTCATGCCATGCCA
      AB621313.1 - Azhul    ATGTGCTACATATATGGAGAAAAAGAGAAAAATATTATCATGTCATGTCATGCCATGCCA
 AB621325.1 - B83-12/21/3    ATGTGCTACATATATGGAGAAAAAGAGAAAAATATTATCATGTCATGTCATGCCATGCCA
       AB621322.1 - Beka    ATGTGCTACATATATGGAGAAAAAGAGAAAAATATTATCATGTCATGTCATGCCATGCCA
   AB621321.1 - CDC Bold    ATGTGCTACATATATGGAGAAAAAGAGAAAAATATTATCATGTCATGTCATGCCATGCCA
      AB621332.1 - Morex    ATGTGCTACATATATGGAGAAAAAGAGAAAAATATTATCATGTCATGTCATGCCATGCCA
AB621319.1 - Nishinohoshi    ATGTGCTACATATATGGAGAAAAAGAGAAAAATATTATCATGTCATGTCATGCCATGCCA

                              Indel2
      AB621318.1 - TR251    -----------AGAAGAAAAAGAAGTGTTGCCACCGCTCGAATGCCTCTTTTTTCTTTCG
  AB621312.1 - CDC Fibar    TGAGAAGGAGGAGAAGAAAAATAAGTGTTGCCACCGCTCGAATGCCTTTTTTTTCTTTCG
      AB621313.1 - Azhul    TGAGAAGGAGGAGAAGAAAAATAAGTGTTGCCACCGCTCGAATGCCTTTTTTTTCTTTCG
 AB621325.1 - B83-12/21/3    TGAGAAGGAGGAGAAGAAAAATAAGTGTTGCCACCGCTCGAATGCCTTTTTTTTCTTTCG
       AB621322.1 - Beka    TGAGAAGGAGGAGAAGAAAAATAAGTGTTGCCACCGCTCGAATGCCTTTTTTTTCTTTCG
   AB621321.1 - CDC Bold    TGAGAAGGAGGAGAAGAAAAAGAAGTGTTGCCACCGCTCGAATGCCTTTTTTTTCTTTCG
      AB621332.1 - Morex    TGAGAAGGAGGAGAAGAAAAAGAAGTGTTGCCACCGCTCGAATGCCTTTTTTTTCTTTCG
AB621319.1 - Nishinohoshi    TGAGAAGGAGGAGAAGAAAAAGAAGTGTTGCCACCGCTCGAATGCCTTTTTTTTCTTTCG

      AB621318.1 - TR251    GAAGGATGCGTGAGTCATGTTGGCACCGAGAAAAGCCATATTAAAGTGGCAGAGTTACAA
  AB621312.1 - CDC Fibar    GAAGGATGCGTAAGTCATGTTGGCACCGAGAAAAGCCATATTAAAGTGGCAGAGTTACAA
      AB621313.1 - Azhul    GAAGGATGCGTAAGTCATGTTGGCACCGAGAAAAGCCATATTAAAGTGGCAGAGTTACAA
 AB621325.1 - B83-12/21/3    GAAGGATGCGTAAGTCATGTTGGCACCGAGAAAAGCCATATTAAAGTGGCAGAGTTACAA
       AB621322.1 - Beka    GAAGGATGCGTAAGTCATGTTGGCACCGAGAAAAGCCATATTAAAGTGGCAGAGTTACAA
   AB621321.1 - CDC Bold    GAAGGATGCGTGAGTCATGTTGGCACCGAGAAAAGCCATATTAAAGTGGCAGAGTTACAA
      AB621332.1 - Morex    GAAGGATGCGTGAGTCATGTTGGCACCGAGAAAAGCCATATTAAAGTGGCAGAGTTACAA
AB621319.1 - Nishinohoshi    GAAGGATGCGTGAGTCATGTTGGCACCGAGAAAAGCCATATTAAAGTGGCAGAGTTACAA

      AB621318.1 - TR251    CTCAGAATAAATGCGGGTGTTACAAAAACACTAGGATATGTGAAGGGCACTCGGCACAAC
  AB621312.1 - CDC Fibar    CTCAGAATAAATGCGGGTGTTACAAAAACACTAGGATATGTGAAGGGCACTCGGCACAAC
      AB621313.1 - Azhul    CTCAGAATAAATGCGGGTGTTACAAAAACACTAGGATATGTGAAGGGCACTCGGCACAAC
 AB621325.1 - B83-12/21/3    CTCAGAATAAATGCGGGTGTTACAAAAACACTAGGATATGTGAAGGGCACTCGGCACAAC
       AB621322.1 - Beka    CTCAGAATAAATGCGGGTGTTACAAAAACACTAGGATATGTGAAGGGCACTCGGCACAAC
   AB621321.1 - CDC Bold    CTCAGAATAAATGCGGGTGTTACAAAAACACTAGGATATGTGAAGGGCACTCGGCACAAC
      AB621332.1 - Morex    CTCAGAATAAATGCGGGTGTTACAAAAACACTAGGATATGTGAAGGGCACTCGGCACAAC
AB621319.1 - Nishinohoshi    CTCAGAATAAATGCGGGTGTTACAAAAACACTAGGATATGTGAAGGGCACTCGGCACAAC

      AB621318.1 - TR251    ACTTTAAGACTACACAATTGAAAAAGTGTACTCTCTGTATCTAAATAATTATAATTGAAA
  AB621312.1 - CDC Fibar    ACTTTAAGACTACACAATTGAAAAAGTGTACTCTCTGTATCTAAATAATTATAATTGAAA
      AB621313.1 - Azhul    ACTTTAAGACTACACAATTGAAAAAGTGTACTCTCTGTATCTAAATAATTATAATTGAAA
 AB621325.1 - B83-12/21/3    ACTTTAAGACTACACAATTGAAAAAGTGTACTCTCTGTATCTAAATAATTATAATTGAAA
       AB621322.1 - Beka    ACTTTAAGACTACACAATTGAAAAAGTGTACTCTCTGTATCTAAATAATTATAATTGAAA
   AB621321.1 - CDC Bold    ACTTTAAGACTACACAATTGAAAAAGTGTACTCTCTGTATCTAAATAATTATAATTGAAA
      AB621332.1 - Morex    ACTTTAAGACTACACAATTGAAAAAGTGTACTCTCTGTATCTAAATAATTATAATTGAAA
AB621319.1 - Nishinohoshi    ACTTTAAGACTACACAATTGAAAAAGTGTACTCTCTGTATCTAAATAATTATAATTGAAA

                                           SNP12
      AB621318.1 - TR251    AGAATTAATCTATATATGAAAGTAGTAATGATTAGCGGTAGAAGGTTCCAACGACTTTTT
  AB621312.1 - CDC Fibar    AGAATTAATCTATATATAAAAGTAGTAATGATTAGCGGTAGAAGGTTCCAACGACTTTTT
      AB621313.1 - Azhul    AGAATTAATCTATATATAAAAGTAGTAATGATTAGCGGTAGAAGGTTCCAACGACTTTTT
 AB621325.1 - B83-12/21/3    AGAATTAATCTATATATAAAAGTAGTAATGATTAGCGGTAGAAGGTTCCAACGACTTTTT
       AB621322.1 - Beka    AGAATTAATCTATATATGAAAGTAGTAATGATTAGCGGTAGAAGGTTCCAACGACTTTTT
   AB621321.1 - CDC Bold    AGAATTAATCTATATATGAAAGTAGTAATGATTAGCGGTAGAAGGTTCCAACGACTTTTT
      AB621332.1 - Morex    AGAATTAATCTATATATGAAAGTAGTAATGATTAGCGGTAGAAGGTTCCAACGACTTTTT
AB621319.1 - Nishinohoshi    AGAATTAATCTATATATGAAAGTAGTAATGATTAGCGGTAGAAGGTTCCAACGACTTTTT

      AB621318.1 - TR251    TGGCGCCAATAGCAAGAAGAAAGAAAAAGAAAAAAATCTTTACTGTACAGTA--ACGAGA
  AB621312.1 - CDC Fibar    TGGCGCCAATAGCAAGAAGAAAGAAAAAGAAAAAAATCTTTACTGTACAGTATAACGAGA
      AB621313.1 - Azhul    TGGCGCCAATAGCAAGAAGAAAGAAAAAGAAAAAAATCTTTACTGTACAGTATAACGAGA
 AB621325.1 - B83-12/21/3    TGGCGCCAATAGCAAGAAGAAAGAAAAAGAAAAAAATCTTTACTGTACAGTATAACGAGA
       AB621322.1 - Beka    TGGCGCCAATAGCAAGAAGAAAGAAAAAGAAAAAAATCTTTACTGTACAGTATAACGAGA
   AB621321.1 - CDC Bold    TGGCGCCAATAGCAAGAAGAAAGAAAAAGAAAAAAATCTTTACTGTACAGTATAACGAGA
      AB621332.1 - Morex    TGGCGCCAATAGCAAGAAGAAAGAAAAAGAAAAAAATCTTTACTGTACAGTATAACGAGA
AB621319.1 - Nishinohoshi    TGGCGCCAATAGCAAGAAGAAAGAAAAAGAAAAAAATCTTTACTGTACAGTATAACGAGA

      AB621318.1 - TR251    AAAGAGGCCCATTAATAGAGCAACGAATCCAGCGGCACCACCTCTGGCGGTCACGTCCAT
  AB621312.1 - CDC Fibar    AAAGAGGCCCATTAATAGAGCAACGAATCCAGCGGCACCACCTCTGGCGGTCACGTCCAT
      AB621313.1 - Azhul    AAAGAGGCCCATTAATAGAGCAACGAATCCAGCGGCACCACCTCTGGCGGTCACGTCCAT
 AB621325.1 - B83-12/21/3    AAAGAGGCCCATTAATAGAGCAACGAATCCAGCGGCACCACCTCTGGCGGTCACGTCCAT
       AB621322.1 - Beka    AAAGAGGCCCATTAATAGAGCAACGAATCCAGCGGCACCACCTCTGGCGGTCACGTCCAT
   AB621321.1 - CDC Bold    AAAGAGGCCCATTAATAGAGCAACGAATCGAGCGGCACCACCTCTGGCGGTCACGTCCAT
      AB621332.1 - Morex    AAAGAGGCCCATTAATAGAGCAACGAATCGAGCGGCACCACCTCTGGCGGTCACGTCCAT
AB621319.1 - Nishinohoshi    AAAGAGGCCCATTAATAGAGCAACGAATCGAGCGGCACCACCTCTGGCGGTCACGTCCAT

      AB621318.1 - TR251    GCCCTCGCACGACGGATGAGGCCCGGGGGGTCCTACTGACAGCCGAAGCATGTCGGTGCT
  AB621312.1 - CDC Fibar    GCCCTCGCACGACGGATGAGGCCCGGGGGGTCCTACTGACAGCCGAAGCATGTCGGTGCT
      AB621313.1 - Azhul    GCCCTCGCACGACGGATGAGGCCCGGGGGGTCCTACTGACAGCCGAAGCATGTCGGTGCT
 AB621325.1 - B83-12/21/3    GCCCTCGCACGACGGATGAGGCCCGGGGGGTCCTACTGACAGCCGAAGCATGTCGGTGCT
       AB621322.1 - Beka    GCCCTCGCACGACGGATGAGGCCCGGGGGGTCCTACTGACAGCCGAAGCATGTCGGTGCT
   AB621321.1 - CDC Bold    GCCCTCGCACGACGGATGAGGCCCGGGGGGTCCTACTGACAGCCGAAGCATGTCGGTGCT
      AB621332.1 - Morex    GCCCTCGCACGACGGATGAGGCCCGGGGGGTCCTACTGACAGCCGAAGCATGTCGGTGCT
AB621319.1 - Nishinohoshi    GCCCTCGCACGACGGATGAGGCCCGGGGGGTCCTACTGACAGCCGAAGCATGTCGGTGCT
```

```
                                                                      SNP14
       AB621318.1 - TR251   CAAACACGGCGCCGTTTGCTGCCAAGTGTGCCAGCTCGCACTCATTGACTTGCCAGCTCT
   AB621312.1 - CDC Fibar   CAAACACGGCGCCGTTTGCTGCCAAGTGTGCCAGCTCGCACTCATTGACTTGCCAGCTCT
       AB621313.1 - Azhul   CAAACACGGCGCCGTTTGCTGCCAAGTGTGCCAGCTCGCACTCATTGACTTGCCAGCTCT
 AB621325.1 - B83-12/21/3   CAAACACGGCGCCGTTTGCTGCCAAGTGTGCCAGCTCGCACTCATTGACTTGCCAGCTCT
        AB621322.1 - Beka   CAAACACGGCGCCGTTTGCTGCCAAGTGTGCCAGCTCGCACTCATTGACTTGCCAGCTCT
    AB621321.1 - CDC Bold   CAAACACGGCGCCGTTTGCTGCCAAGTGTGCCAGCTCGCACTCATTGACTTGCCAGCTCT
       AB621332.1 - Morex   CAAACACGGCGCCGTTTGCTGCCAAGTGTGCCAGCCCGCACTCATTGACTTGCCAGCTCT
AB621319.1 - Nishinohoshi   CAAACACGGCGCCGTTTGCTGCCAAGTGTGCCAGCTCGCACTCATTGACTTGCCAGCTCT

       AB621318.1 - TR251   CTCCTTGGTTGTCAATGAGAACATGATGCCTTTTGGCATTTGCAAACTTATTAAAACTAG
   AB621312.1 - CDC Fibar   CTCCTTGGTTGTCAATGAGAACATGATGCCTTTTGGCATTTGCAAACTTATTAAAACTAG
       AB621313.1 - Azhul   CTCCTTGGTTGTCAATGAGAACATGATGCCTTTTGGCATTTGCAAACTTATTAAAACTAG
 AB621325.1 - B83-12/21/3   CTCCTTGGTTGTCAATGAGAACATGATGCCTTTTGGCATTTGCAAACTTATTAAAACTAG
        AB621322.1 - Beka   CTCCTTGGTTGTCAATGAGAACATGATGCCTTTTGGCATTTGCAAACTTATTAAAACTAG
    AB621321.1 - CDC Bold   CTCCTTGGTTGTCAATGAGAACATGATGCCTTTTGGCATTTGCAAACTTATTAAAACTAG
       AB621332.1 - Morex   CTCCTTGGTTGTCAATGAGAACATGATGCCTTTTGGCATTTGCAAACTTATTAAAACTAG
AB621319.1 - Nishinohoshi   CTCCTTGGTTGTCAATGAGAACATGATGCCTTTTGGCATTTGCAAACTTATTAAAACTAG

       AB621318.1 - TR251   CTGTCGTCCGATAGGGAAAAGAAAAGAAAAGAAAAGAATAAGAAAAAAAGGACAAAGAGA
   AB621312.1 - CDC Fibar   CTGTCGTCCGATAGGGAAAAGAAAAGAAAAGAAAAGAATAAGAAAAAAAGGACAAAGAGA
       AB621313.1 - Azhul   CTGTCGTCCGATAGGGAAAAGAAAAGAAAAGAAAAGAATAAGAAAAAAAGGACAAAGAGA
 AB621325.1 - B83-12/21/3   CTGTCGTCCGATAGGGAAAAGAAAAGAAAAGAAAAGAATAAGAAAAAAAGGACAAAGAGA
        AB621322.1 - Beka   CTGTCGTCCGATAGGGAAAAGAAAAGAAAAGAAAAGAATAAGAAAAAAAGGACAAAGAGA
    AB621321.1 - CDC Bold   CTGTCGTCCGATAGGGAAAAGAAAAGAAAAGAAAAGAATAAGAAAAAAAGGACAAAGAGA
       AB621332.1 - Morex   CTGTCGTCCGATAGGGAAAAGAAAAGAAAAGAAAAGAATAAGAAAAAAAGGACAAAGAGA
AB621319.1 - Nishinohoshi   CTGTCGTCCGATAGGGAAAAGAAAAGAAAAGAAAAGAATAAGAAAAAAAGGACAAAGAGA

       AB621318.1 - TR251   AAAGATGAACATGGCGCATGTTCCCTCCAATAATTGCAGGCACCAACACTGGGTCGATTA
   AB621312.1 - CDC Fibar   AAAGATGAACATGGCGCATGTTCCCTCCAATAATTGCAGGCACCAACACTGGGTCGATTA
       AB621313.1 - Azhul   AAAGATGAACATGGCGCATGTTCCCTCCAATAATTGCAGGCACCAACACTGGGTCGATTA
 AB621325.1 - B83-12/21/3   AAAGATGAACATGGCGCATGTTCCCTCCAATAATTGCAGGCACCAACACTGGGTCGATTA
        AB621322.1 - Beka   AAAGATGAACATGGCGCATGTTCCCTCCAATAATTGCAGGCACCAACACTGGGTCGATTA
    AB621321.1 - CDC Bold   AAAGATGAACATGGCGCATGTTCCCTCCAATAATTGCAGGCACCAACACTGGGTCGATTA
       AB621332.1 - Morex   AAAGATGAACATGGCGCATGTTCCCTCCAATAATTGCAGGCACCAACACTGGGTCGATTA
AB621319.1 - Nishinohoshi   AAAGATGAACATGGCGCATGTTCCCTCCAATAATTGCAGGCACCAACACTGGGTCGATTA

       AB621318.1 - TR251   ATCCAACAACAATATTTTACTATACCAGACGAGAGTACAGTAGTCGGGTGATGATGGACT
   AB621312.1 - CDC Fibar   ATCCAACAACAATATTTTACTATACCAGACGAGAGTACAGTAGTCGGGTGATGATGGACT
       AB621313.1 - Azhul   ATCCAACAACAATATTTTACTATACCAGACGAGAGTACAGTAGTCGGGTGATGATGGACT
 AB621325.1 - B83-12/21/3   ATCCAACAACAATATTTTACTATACCAGACGAGAGTACAGTAGTCGGGTGATGATGGACT
        AB621322.1 - Beka   ATCCAACAACAATATTTTACTATACCAGACGAGAGTACAGTAGTCGGGTGATGATGGACT
    AB621321.1 - CDC Bold   ATCCAACAACAATATTTTACTATACCAGACGAGAGTACAGTAGTCGGGTGATGATGGACT
       AB621332.1 - Morex   ATCCAACAACAATATTTTACTATACCAGACGAGAGTACAGTAGTCGGGTGATGATGGACT
AB621319.1 - Nishinohoshi   ATCCAACAACAATATTTTACTATACCAGACGAGAGTACAGTAGTCGGGTGATGATGGACT

       AB621318.1 - TR251   GTAACTGACTGAGTATGAATGACTGTAATGCAGGGTGCTGATTTTCGTTCGTCTGATCGC
   AB621312.1 - CDC Fibar   GTAACTGACTGAGTATGAATGACTGTAATGCAGGGTGCTGATTTTCGTTCGTCTGATCGC
       AB621313.1 - Azhul   GTAACTGACTGAGTATGAATGACTGTAATGCAGGGTGCTGATTTTCGTTCGTCTGATCGC
 AB621325.1 - B83-12/21/3   GTAACTGACTGAGTATGAATGACTGTAATGCAGGGTGCTGATTTTCGTTCGTCTGATCGC
        AB621322.1 - Beka   GTAACTGACTGAGTATGAATGACTGTAATGCAGGGTGCTGATTTTCGTTCGTCTGATCGC
    AB621321.1 - CDC Bold   GTAACTGACTGAGTATGAATGACTGTAATGCAGGGTGCTGATTTTCGTTCGTCTGATCGC
       AB621332.1 - Morex   GTAACTGACTGAGTATGAATGACTGTAATGCAGGGTGCTGATTTTCGTTCGTCTGATCGC
AB621319.1 - Nishinohoshi   GTAACTGACTGAGTATGAATGACTGTAATGCAGGGTGCTGATTTTCGTTCGTCTGATCGC

       AB621318.1 - TR251   CTTCACGCTGTTCGTGATCTGGCGTATCTCCCACAAGAACCCAGACGCGATGTGGCTGTG
   AB621312.1 - CDC Fibar   CTTCACGCTGTTCGTGATCTGGCGTATCTCCCACAAGAACCCAGACGCGATGTGGCTGTG
       AB621313.1 - Azhul   CTTCACGCTGTTCGTGATCTGGCGTATCTCCCACAAGAACCCAGACGCGATGTGGCTGTG
 AB621325.1 - B83-12/21/3   CTTCACGCTGTTCGTGATCTGGCGTATCTCCCACAAGAACCCAGACGCGATGTGGCTGTG
        AB621322.1 - Beka   CTTCACGCTGTTCGTGATCTGGCGTATCTCCCACAAGAACCCAGACGCGATGTGGCTGTG
    AB621321.1 - CDC Bold   CTTCACGCTGTTCGTGATCTGGCGTATCTCCCACAAGAACCCAGACGCGATGTGGCTGTG
       AB621332.1 - Morex   CTTCACGCTGTTCGTGATCTGGCGTATCTCCCACAAGAACCCAGACGCGATGTGGCTGTG
AB621319.1 - Nishinohoshi   CTTCACGCTGTTCGTGATCTGGCGTATCTCCCACAAGAACCCAGACGCGATGTGGCTGTG

       AB621318.1 - TR251   GGTGACATCCATCTGCGGCGAGTTCTGGTTCGGTTTCTCGTGGCTGCTGGATCAGCTGCC
   AB621312.1 - CDC Fibar   GGTGACATCCATCTGCGGCGAGTTCTGGTTCGGTTTCTCGTGGCTGCTGGATCAGCTGCC
       AB621313.1 - Azhul   GGTGACATCCATCTGCGGCGAGTTCTGGTTCGGTTTCTCGTGGCTGCTGGATCAGCTGCC
 AB621325.1 - B83-12/21/3   GGTGACATCCATCTGCGGCGAGTTCTGGTTCGGTTTCTCGTGGCTGCTGGATCAGCTGCC
        AB621322.1 - Beka   GGTGACATCCATCTGCGGCGAGTTCTGGTTCGGTTTCTCGTGGCTGCTGGATCAGCTGCC
    AB621321.1 - CDC Bold   GGTGACATCCATCTGCGGCGAGTTCTGGTTCGGTTTCTCGTGGCTGCTGGATCAGCTGCC
       AB621332.1 - Morex   GGTGACATCCATCTGCGGCGAGTTCTGGTTCGGTTTCTCGTGGCTGCTGGATCAGCTGCC
AB621319.1 - Nishinohoshi   GGTGACATCCATCTGCGGCGAGTTCTGGTTCGGTTTCTCGTGGCTGCTGGATCAGCTGCC

       AB621318.1 - TR251   CAAGCTGAACCCCATCAACCGCGTGCCGGACCTGGCGGTGCTGCGGCAGCGCTTCGACCG
   AB621312.1 - CDC Fibar   CAAGCTGAACCCCATCAACCGCGTGCCGGACCTGGCGGTGCTGCGGCAGCGCTTCGACCG
       AB621313.1 - Azhul   CAAGCTGAACCCCATCAACCGCGTGCCGGACCTGGCGGTGCTGCGGCAGCGCTTCGACCG
 AB621325.1 - B83-12/21/3   CAAGCTGAACCCCATCAACCGCGTGCCGGACCTGGCGGTGCTGCGGCAGCGCTTCGACCG
        AB621322.1 - Beka   CAAGCTGAACCCCATCAACCGCGTGCCGGACCTGGCGGTGCTGCGGCAGCGCTTCGACCG
    AB621321.1 - CDC Bold   CAAGCTGAACCCCATCAACCGCGTGCCGGACCTGGCGGTGCTGCGGCAGCGCTTCGACCG
       AB621332.1 - Morex   CAAGCTGAACCCCATCAACCGCGTGCCGGACCTGGCGGTGCTGCGGCAGCGCTTCGACCG
AB621319.1 - Nishinohoshi   CAAGCTGAACCCCATCAACCGCGTGCCGGACCTGGCGGTGCTGCGGCAGCGCTTCGACCG
```

```
      AB621318.1 - TR251   CCCCGACGGCACCTCCACGCTCCCGGGGCTGGACATCTTCGTCACCACGGCCGACCCCAT
  AB621312.1 - CDC Fibar   CCCCGACGGCACCTCCACGCTCCCGGGGCTGGACATCTTCGTCACCACGGCCGACCCCAT
      AB621313.1 - Azhul   CCCCGACGGCACCTCCACGCTCCCGGGGCTGGACATCTTCGTCACCACGGCCGACCCCAT
AB621325.1 - B83-12/21/3   CCCCGACGGCACCTCCACGCTCCCGGGGCTGGACATCTTCGTCACCACGGCCGACCCCAT
       AB621322.1 - Beka   CCCCGACGGCACCTCCACGCTCCCGGGGCTGGACATCTTCGTCACCACGGCCGACCCCAT
   AB621321.1 - CDC Bold   CCCCGACGGCACCTCCACGCTCCCGGGGCTGGACATCTTCGTCACCACGGCCGACCCCAT
      AB621332.1 - Morex   CCCCGACGGCACCTCCACGCTCCCGGGGCTGGACATCTTCGTCACCACGGCCGACCCCAT
AB621319.1 - Nishinohoshi  CCCCGACGGCACCTCCACGCTCCCGGGGCTGGACATCTTCGTCACCACGGCCGACCCCAT

      AB621318.1 - TR251   CAAGGAGCCCATCCTCTCCACCGCCAACTCGGTGCTCTCCATCCTGGCCGCCGACTACCC
  AB621312.1 - CDC Fibar   CAAGGAGCCCATCCTCTCCACCGCCAACTCGGTGCTCTCCATCCTGGCCGCCGACTACCC
      AB621313.1 - Azhul   CAAGGAGCCCATCCTCTCCACCGCCAACTCGGTGCTCTCCATCCTGGCCGCCGACTACCC
AB621325.1 - B83-12/21/3   CAAGGAGCCCATCCTCTCCACCGCCAACTCGGTGCTCTCCATCCTGGCCGCCGACTACCC
       AB621322.1 - Beka   CAAGGAGCCCATCCTCTCCACCGCCAACTCGGTGCTCTCCATCCTGGCCGCCGACTACCC
   AB621321.1 - CDC Bold   CAAGGAGCCCATCCTCTCCACCGCCAACTCGGTGCTCTCCATCCTGGCCGCCGACTACCC
      AB621332.1 - Morex   CAAGGAGCCCATCCTCTCCACCGCCAACTCGGTGCTCTCCATCCTGGCCGCCGACTACCC
AB621319.1 - Nishinohoshi  CAAGGAGCCCATCCTCTCCACCGCCAACTCGGTGCTCTCCATCCTGGCCGCCGACTACCC

      AB621318.1 - TR251   CGTGGACCGCAACACATGCTACGTCTCCGACGACAGTGGCATGCTGCTCACCTACGAGGC
  AB621312.1 - CDC Fibar   CGTGGACCGCAACACATGCTACGTCTCCGACGACAGTGGCATGCTGCTCACCTACGAGGC
      AB621313.1 - Azhul   CGTGGACCGCAACACATGCTACGTCTCCGACGACAGTGGCATGCTGCTCACCTACGAGGC
AB621325.1 - B83-12/21/3   CGTGGACCGCAACACATGCTACGTCTCCGACGACAGTGGCATGCTGCTCACCTACGAGGC
       AB621322.1 - Beka   CGTGGACCGCAACACATGCTACGTCTCCGACGACAGTGGCATGCTGCTCACCTACGAGGC
   AB621321.1 - CDC Bold   CGTGGACCGCAACACATGCTACGTCTCCGACGACAGTGGCATGCTGCTCACCTACGAGGC
      AB621332.1 - Morex   CGTGGACCGCAACACATGCTACGTCTCCGACGACAGTGGCATGCTGCTCACCTACGAGGC
AB621319.1 - Nishinohoshi  CGTGGACCGCAACACATGCTACGTCTCCGACGACAGTGGCATGCTGCTCACCTACGAGGC

      AB621318.1 - TR251   CCTGGCAGAGTCCTCCAAGTTCGCCACGCTCTGGGTGCCCTTCTGCCGCAAGCACGGGAT
  AB621312.1 - CDC Fibar   CCTGGCAGAGTCCTCCAAGTTCGCCACGCTCTGGGTGCCCTTCTGCCGCAAGCACGGGAT
      AB621313.1 - Azhul   CCTGGCAGAGTCCTCCAAGTTCGCCACGCTCTGGGTGCCCTTCTGCCGCAAGCACGGGAT
AB621325.1 - B83-12/21/3   CCTGGCAGAGTCCTCCAAGTTCGCCACGCTCTGGGTGCCCTTCTGCCGCAAGCACGGGAT
       AB621322.1 - Beka   CCTGGCAGAGTCCTCCAAGTTCGCCACGCTCTGGGTGCCCTTCTGCCGCAAGCACGGGAT
   AB621321.1 - CDC Bold   CCTGGCAGAGTCCTCCAAGTTCGCCACGCTCTGGGTGCCCTTCTGCCGCAAGCACGGGAT
      AB621332.1 - Morex   CCTGGCAGAGTCCTCCAAGTTCGCCACGCTCTGGGTGCCCTTCTGCCGCAAGCACGGGAT
AB621319.1 - Nishinohoshi  CCTGGCAGAGTCCTCCAAGTTCGCCACGCTCTGGGTGCCCTTCTGCCGCAAGCACGGGAT

      AB621318.1 - TR251   CGAGCCCAGGGGTCCGGAGAGCTACTTCGAGCTCAAGTCACACCCTTACATGGGGAGAGC
  AB621312.1 - CDC Fibar   CGAGCCCAGGGGTCCGGAGAGCTACTTCGAGCTCAAGTCACACCCTTACATGGGGAGAGC
      AB621313.1 - Azhul   CGAGCCCAGGGGTCCGGAGAGCTACTTCGAGCTCAAGTCACACCCTTACATGGGGAGAGC
AB621325.1 - B83-12/21/3   CGAGCCCAGGGGTCCGGAGAGCTACTTCGAGCTCAAGTCACACCCTTACATGGGGAGAGC
       AB621322.1 - Beka   CGAGCCCAGGGGTCCGGAGAGCTACTTCGAGCTCAAGTCACACCCTTACATGGGGAGAGC
   AB621321.1 - CDC Bold   CGAGCCCAGGGGTCCGGAGAGCTACTTCGAGCTCAAGTCACACCCTTACATGGGGAGAGC
      AB621332.1 - Morex   CGAGCCCAGGGGTCCGGAGAGCTACTTCGAGCTCAAGTCACACCCTTACATGGGGAGAGC
AB621319.1 - Nishinohoshi  CGAGCCCAGGGGTCCGGAGAGCTACTTCGAGCTCAAGTCACACCCTTACATGGGGAGAGC

      AB621318.1 - TR251   CCAGGACGAGTTCGTCAACGACCGCCGCCGCGTTCGCAAGGAGTACGACGAGTTCAAGGC
  AB621312.1 - CDC Fibar   CCAGGACGAGTTCGTCAACGACCGCCGCCGCGTTCGCAAGGAGTACGACGAGTTCAAGGC
      AB621313.1 - Azhul   CCAGGACGAGTTCGTCAACGACCGCCGCCGCGTTCGCAAGGAGTACGACGAGTTCAAGGC
AB621325.1 - B83-12/21/3   CCAGGACGAGTTCGTCAACGACCGCCGCCGCGTTCGCAAGGAGTACGACGAGTTCAAGGC
       AB621322.1 - Beka   CCAGGACGAGTTCGTCAACGACCGCCGCCGCGTTCGCAAGGAGTACGACGAGTTCAAGGC
   AB621321.1 - CDC Bold   CCAGGACGAGTTCGTCAACGACCGCCGCCGCGTTCGCAAGGAGTACGACGAGTTCAAGGC
      AB621332.1 - Morex   CCAGGACGAGTTCGTCAACGACCGCCGCCGCGTTCGCAAGGAGTACGACGAGTTCAAGGC
AB621319.1 - Nishinohoshi  CCAGGACGAGTTCGTCAACGACCGCCGCCGCGTTCGCAAGGAGTACGACGAGTTCAAGGC

      AB621318.1 - TR251   CAGGATCAACAGCCTGGAGCATGACATCAAGCAGCGCAACGACGGGTACAACGCCGCCAT
  AB621312.1 - CDC Fibar   CAGGATCAACAGCCTGGAGCATGACATCAAGCAGCGCAACGACGGGTACAACGCCGCCAT
      AB621313.1 - Azhul   CAGGATCAACAGCCTGGAGCATGACATCAAGCAGCGCAACGACGGGTACAACGCCGCCAT
AB621325.1 - B83-12/21/3   CAGGATCAACAGCCTGGAGCATGACATCAAGCAGCGCAACGACGGGTACAACGCCGCCAT
       AB621322.1 - Beka   CAGGATCAACAGCCTGGAGCATGACATCAAGCAGCGCAACGACGGGTACAACGCCGCCAT
   AB621321.1 - CDC Bold   CAGGATCAACAGCCTGGAGCATGACATCAAGCAGCGCAACGACGGGTACAACGCCGCCAT
      AB621332.1 - Morex   CAGGATCAACAGCCTGGAGCATGACATCAAGCAGCGCAACGACGGGTACAACGCCGCCAT
AB621319.1 - Nishinohoshi  CAGGATCAACAGCCTGGAGCATGACATCAAGCAGCGCAACGACGGGTACAACGCCGCCAT

      AB621318.1 - TR251   TGCCCACAGCCAAGGCGTGCCCCGGCCCACCTGGATGGCGGACGGCACCCAGTGGGAGGG
  AB621312.1 - CDC Fibar   TGCCCACAGCCAAGGCGTGCCCCGGCCCACCTGGATGGCGGACGGCACCCAGTGGGAGGG
      AB621313.1 - Azhul   TGCCCACAGCCAAGGCGTGCCCCGGCCCACCTGGATGGCGGACGGCACCCAGTGGGAGGG
AB621325.1 - B83-12/21/3   TGCCCACAGCCAAGGCGTGCCCCGGCCCACCTGGATGGCGGACGGCACCCAGTGGGAGGG
       AB621322.1 - Beka   TGCCCACAGCCAAGGCGTGCCCCGGCCCACCTGGATGGCGGACGGCACCCAGTGGGAGGG
   AB621321.1 - CDC Bold   TGCCCACAGCCAAGGCGTGCCCCGGCCCACCTGGATGGCGGACGGCACCCAGTGGGAGGG
      AB621332.1 - Morex   TGCCCACAGCCAAGGCGTGCCCCGGCCCACCTGGATGGCGGACGGCACCCAGTGGGAGGG
AB621319.1 - Nishinohoshi  TGCCCACAGCCAAGGCGTGCCCCGGCCCACCTGGATGGCGGACGGCACCCAGTGGGAGGG

      AB621318.1 - TR251   CACATGGGTCGACGCCTCCGAGAACCACCGCAGGGGCGACCACGCCGGCATCGTACTGGT
  AB621312.1 - CDC Fibar   CACATGGGTCGACGCCTCCGAGAACCACCGCAGGGGCGACCACGCCGGCATCGTACTGGT
      AB621313.1 - Azhul   CACATGGGTCGACGCCTCCGAGAACCACCGCAGGGGCGACCACGCCGGCATCGTACTGGT
AB621325.1 - B83-12/21/3   CACATGGGTCGACGCCTCCGAGAACCACCGCAGGGGCGACCACGCCGGCATCGTACTGGT
       AB621322.1 - Beka   CACATGGGTCGACGCCTCCGAGAACCACCGCAGGGGCGACCACGCCGGCATCGTACTGGT
   AB621321.1 - CDC Bold   CACATGGGTCGACGCCTCCGAGAACCACCGCAGGGGCGACCACGCCGGCATCGTACTGGT
      AB621332.1 - Morex   CACATGGGTCGACGCCTCCGAGAACCACCGCAGGGGCGACCACGCCGGCATCGTACTGGT
AB621319.1 - Nishinohoshi  CACATGGGTCGACGCCTCCGAGAACCACCGCAGGGGCGACCACGCCGGCATCGTACTGGT
```

```
      AB621318.1 - TR251   CAGTATCCATCCATCTTTCTGCTGCTTATATTACTCTTAGGTTACTCTTATCGTCTCTTT
  AB621312.1 - CDC Fibar   CAGTATCCATCCATCTTTCTGCTGCTTATATTACTCTTAGGTTACTCTTATCGTCTCTTT
      AB621313.1 - Azhul   CAGTATCCATCCATCTTTCTGCTGCTTATATTACTCTTAGGTTACTCTTATCGTCTCTTT
 AB621325.1 - B83-12/21/3   CAGTATCCATCCATCTTTCTGCTGCTTATATTACTCTTAGGTTACTCTTATCGTCTCTTT
       AB621322.1 - Beka   CAGTATCCATCCATCTTTCTGCTGCTTATATTACTCTTAGGTTACTCTTATCGTCTCTTT
   AB621321.1 - CDC Bold   CAGTATCCATCCATCTTTCTGCTGCTTATATTACTCTTAGGTTACTCTTATCGTCTCTTT
      AB621332.1 - Morex   CAGTATCCATCCATCTTTCTGCTGCTTATATTACTCTTAGGTTACTCTTATCGTCTCTTT
AB621319.1 - Nishinohoshi   CAGTATCCATCCATCTTTCTGCTGCTTATATTACTCTTAGGTTACTCTTATCGTCTCTTT

      AB621318.1 - TR251   CCTATACCGTACATGCATGCATGCTGCTATTCTTGGAATCGTGGTTGGTTACTACTCCAC
  AB621312.1 - CDC Fibar   CCTATACTGTACATGCATGCATGCTGCTATTCTTGGAATCGTGGTTGGTTACTACTCCAC
      AB621313.1 - Azhul   CCTATACTGTACATGCATGCATGCTGCTATTCTTGGAATCGTGGTTGGTTACTACTCCAC
 AB621325.1 - B83-12/21/3   CCTATACTGTACATGCATGCATGCTGCTATTCTTGGAATCGTGGTTGGTTACTACTCCAC
       AB621322.1 - Beka   CCTATACTGTACATGCATGCATGCTGCTATTCTTGGAATCGTGGTTGGTTACTACTCCAC
   AB621321.1 - CDC Bold   CCTATACTGTACATGCATGCATGCTGCTATTCTTGGAATCGTGGTTGGTTACTACTCCAC
      AB621332.1 - Morex   CCTATACTGTACATGCATGCATGCTGCTATTCTTGGAATCGTGGTTGGTTACTACTCCAC
AB621319.1 - Nishinohoshi   CCTATACTGTACATGCATGCATGCTGCTATTCTTGGAATCGTGGTTGGTTACTACTCCAC

      AB621318.1 - TR251   CATGCAAAAATAACAAGAAGAGGAATCTTGGTTAGTTAGGGCCTCGTTGTTATATTAGTG
  AB621312.1 - CDC Fibar   CATGCAAAAATAACAAGAAGAGGAATCTTGGTTAGTTAGGGCCTCGTTGTTATATTAGTG
      AB621313.1 - Azhul   CATGCAAAAATAACAAGAAGAGGAATCTTGGTTAGTTAGGGCCTCGTTGTTATATTAGTG
 AB621325.1 - B83-12/21/3   CATGCAAAAATAACAAGAAGAGGAATCTTGGTTAGTTAGGGCCTCGTTGTTATATTAGTG
       AB621322.1 - Beka   CATGCAAAAATAACAAGAAGAGGAATCTTGGTTAGTTAGGGCCTCGTTGTTATATTAGTG
   AB621321.1 - CDC Bold   CATGCAAAAATAACAAGAAGAGGAATCTTGGTTAGTTAGGGCCTCGTTGTTATATTAGTG
      AB621332.1 - Morex   CATGCAAAAATAACAAGAAGAGGAATCTTGGTTAGTTAGGGCCTCGTTGTTATATTAGTG
AB621319.1 - Nishinohoshi   CATGCAAAAATAACAAGAAGAGGAATCTTGGTTAGTTAGGGCCTCGTTGTTATATTAGTG

      AB621318.1 - TR251   GCCATCTGATGTGATGCCTGCCGGCTGTGCCCATCCATATCCATGGAAGATTTCGACAGA
  AB621312.1 - CDC Fibar   GCCATCTGATGTGATGCCTGCCGGCTGTGCCCATCCATATCCATGGAAGATTTCGACAGA
      AB621313.1 - Azhul   GCCATCTGATGTGATGCCTGCCGGCTGTGCCCATCCATATCCATGGAAGATTTCGACAGA
 AB621325.1 - B83-12/21/3   GCCATCTGATGTGATGCCTGCCGGCTGTGCCCATCCATATCCATGGAAGATTTCGACAGA
       AB621322.1 - Beka   GCCATCTGATGTGATGCCTGCCGGCTGTGCCCATCCATATCCATGGAAGATTTCGACAGA
   AB621321.1 - CDC Bold   GCCATCTGATGTGATGCCTGCCGGCTGTGCCCATCCATATCCATGGAAGATTTCGACAGA
      AB621332.1 - Morex   GCCATCTGATGTGATGCCTGCCGGCTGTGCCCATCCATATCCATGGAAGATTTCGACAGA
AB621319.1 - Nishinohoshi   GCCATCTGATGTGATGCCTGCCGGCTGTGCCCATCCATATCCATGGAAGATTTCGACAGA

      AB621318.1 - TR251   ATCGACGTGGTGATAGTTGAGAGTGCAACCACCACCCAGAGCCAGCCAAGCACATGCATG
  AB621312.1 - CDC Fibar   ATCGACGTGGTGATAGTCGAGAGTGCAACCACCACCCAGAGCCAGCCAAGCACATGCATG
      AB621313.1 - Azhul   ATCGACGTGGTGATAGTCGAGAGTGCAACCACCACCCAGAGCCAGCCAAGCACATGCATG
 AB621325.1 - B83-12/21/3   ATCGACGTGGTGATAGTCGAGAGTGCAACCACCACCCAGAGCCAGCCAAGCACATGCATG
       AB621322.1 - Beka   ATCGACGTGGTGATAGTCGAGAGTGCAACCACCACCCAGAGCCAGCCAAGCACATGCATG
   AB621321.1 - CDC Bold   ATCGACGTGGTGATAGTCGAGAGTGCAACCACCACCCAGAGCCAGCCAAGCACATGCATG
      AB621332.1 - Morex   ATCGACGTGGTGATAGTCGAGAGTGCAACCACCACCCAGAGCCAGCCAAGCACATGCATG
AB621319.1 - Nishinohoshi   ATCGACGTGGTGATAGTCGAGAGTGCAACCACCACCCAGAGCCAGCCAAGCACATGCATG

      AB621318.1 - TR251   CTTCTCTTCTCGTCTCGTCGTGTGGCCAGCAGCGCATTCATGCTATTGCTGTGACGAGGG
  AB621312.1 - CDC Fibar   CTTCTCTTCTCGTCTCGTCGTGTGGCCAGCAGCGCATTCATGCTATTGCTGTGACGAGGG
      AB621313.1 - Azhul   CTTCTCTTCTCGTCTCGTCGTGTGGCCAGCAGCGCATTCATGCTATTGCTGTGACGAGGG
 AB621325.1 - B83-12/21/3   CTTCTCTTCTCGTCTCGTCGTGTGGCCAGCAGCGCATTCATGCTATTGCTGTGACGAGGG
       AB621322.1 - Beka   CTTCTCTTCTCGTCTCGTCGTGTGGCCAGCAGCGCATTCATGCTATTGCTGTGACGAGGG
   AB621321.1 - CDC Bold   CTTCTCTTCTCGTCTCGTCGTGTGGCCAGCAGCGCATTCATGCTATTGCTGTGACGAGGG
      AB621332.1 - Morex   CTTCTCTTCTCGTCTCGTCGTGTGGCCAGCAGCGCATTCATGCTATTGCTGTGACGAGGG
AB621319.1 - Nishinohoshi   CTTCTCTTCTCGTCTCGTCGTGTGGCCAGCAGCGCATTCATGCTATTGCTGTGACGAGGG

      AB621318.1 - TR251   AGGAATGGTGGTTGGGGTGGTCCTTTCCCCCCGACAGCACTACAGCCTCCACTTTATGAC
  AB621312.1 - CDC Fibar   AGGAATGGTGGTTGGGGTGGTCCTTTCCCCCCGACAGCACTACAGCCTCCACTTTATGAC
      AB621313.1 - Azhul   AGGAATGGTGGTTGGGGTGGTCCTTTCCCCCCGACAGCACTACAGCCTCCACTTTATGAC
 AB621325.1 - B83-12/21/3   AGGAATGGTGGTTGGGGTGGTCCTTTCCCCCCGACAGCACTACAGCCTCCACTTTATGAC
       AB621322.1 - Beka   AGGAATGGTGGTTGGGGTGGTCCTTTCCCCCCGACAGCACTACAGCCTCCACTTTATGAC
   AB621321.1 - CDC Bold   AGGAATGGTAGTTGGGGTGGTCCTTTCCCCCCGACAGCACTACAGCCTCCACTTTATGAC
      AB621332.1 - Morex   AGGAATGGTAGTTGGGGTGGTCCTTTCCCCCCGACAGCACTACAGCCTCCACTTTATGAC
AB621319.1 - Nishinohoshi   AGGAATGGTAGTTGGGGTGGTCCTTTCCCCCCGACAGCACTACAGCCTCCACTTTATGAC

      AB621318.1 - TR251   CCATTTAATTCATCGGCCCTGCTTTGTTGTAACCGCCTTCTCACCTCAATCAATCATTCA
  AB621312.1 - CDC Fibar   CCATTTAATTCACCGGCCCTGCTTTGTTGTAACCGCCTTCTCACCTCAATCAATCATTCA
      AB621313.1 - Azhul   CCATTTAATTCACCGGCCCTGCTTTGTTGTAACCGCCTTCTCACCTCAATCAATCATTCA
 AB621325.1 - B83-12/21/3   CCATTTAATTCACCGGCCCTGCTTTGTTGTAACCGCCTTCTCACCTCAATCAATCATTCA
       AB621322.1 - Beka   CCATTTAATTCACCGGCCCTGCTTTGTTGTAACCGCCTTCTCACCTCAATCAATCATTCA
   AB621321.1 - CDC Bold   CCATTTAATTCACCGGCCCTGCTTTGTTGTAACCGCCTTCTCATCTCAATCAATCATTCA
      AB621332.1 - Morex   CCATTTAATTCACCGGCCCTGCTTTGTTGTAACCGCCTTCTCATCTCAATCAATCATTCA
AB621319.1 - Nishinohoshi   CCATTTAATTCACCGGCCCTGCTTTGTTGTAACCGCCTTCTCACCTCAATCAATCATTCA

      AB621318.1 - TR251   TTCATTCATAAGTTTACTCACTCTTTGTTACTACTCGAACCACTAATCAGGAAGGAGTAG
  AB621312.1 - CDC Fibar   TTCATTCATAAGTTTACTCACTCTTTGTTACTACTCGAACCACTAATCAGGAAGGAGTAG
      AB621313.1 - Azhul   TT----CATAAGTTTACTCACTCTTTGTTACTACTCGAACCACTAATCAGGAAGGAGTAG
 AB621325.1 - B83-12/21/3   TTCATTCATAAGTTTACTCACTCTTTGTTACTACTCGAACCACTAATCAGGAAGGAGTAG
       AB621322.1 - Beka   TTCATTCATAAGTTTACTCACTCTTTGTTACTACTCGAACCACTAATCAGGAAGGAGTAG
   AB621321.1 - CDC Bold   TTCATTCATAAGTTTACTCACTCTTTGTTACTACTCGAACCACTAATCAGGAAGGAGTAG
      AB621332.1 - Morex   TTCATTCATAAGTTTACTCACTCTTTGTTACTACTCGAACCACTAATCAGGAAGGAGTAG
AB621319.1 - Nishinohoshi   TTCATTCATAAGTTTACTCACTCTTTGTTACTACTCGAACCACTAATCAGGAAGGAGTAG
```

```
                                                 SNP21
         AB621318.1 - TR251   GAGTAATGCAGATTTACTATTAACAGTTAAAGGAGTAAAAAGAAGGAAGCACAATTACAG
     AB621312.1 - CDC Fibar   GAGTAATGCAGATTTACTATTAACAGTTAAAGGAGTAAAAAGAAGGAAGCACAATTACAG
         AB621313.1 - Azhul   GAGTAATGCAGATTTACTATTAACAGTTAAAGGAGTAAAAAGAAGGAAGCACAATTACAG
   AB621325.1 - B83-12/21/3   GAGTAATGCAGATTTACTATTAACAGTTAAAGGAGTAAAAAGAAGGAAGCACAATTACAG
          AB621322.1 - Beka   GAGTAATGCAGATTTACTATTAACAGTTAAAGGAGTAAAAAGAAGGAAGCACAATTACAG
      AB621321.1 - CDC Bold   GAGTAATGCAGATTTACTATTGACAGTTAAAGGAGTAAAAAGAAGGAAGCACAATTACAG
         AB621332.1 - Morex   GAGTAATGCAGATTTACTATTGACAGTTAAAGGAGTAAAAAGAAGGAAGCACAATTACAG
 AB621319.1 - Nishinohoshi   GAGTAATGCAGATTTACTATTAACAGTTAAAGGAGTAAAAAGAAGGAAGCACAATTACAG

         AB621318.1 - TR251   AACCTTGTTTTTTTTT-ACTACTGTACGTAAGGTGTAAGAATGGAGTGCTGACAGAGAAT
     AB621312.1 - CDC Fibar   AACCTTGTTTTTTTTTTACTACTGTACGTAAGGTGTAAGAATGGAGTGCTGACAGAGAAT
         AB621313.1 - Azhul   AACCTTGTTTTTTTTT-ACTACTGTACGTAAGGTGTAAGAATGGAGTGCTGACAGAGAAT
   AB621325.1 - B83-12/21/3   AACCTTGTTTTTTTTT-ACTACTGTACGTAAGGTGTAAGAATGGAGTGCTGACAGAGAAT
          AB621322.1 - Beka   AACCTTGTTTTTTTTT-ACTACTGTACGTAAGGTGTAAGAATGGAGTGCTGACAGAGAAT
      AB621321.1 - CDC Bold   AACCTTGTTTTTTTTT-ACTACTGTACGTAAGGTGTAAGAATGGAGTGCTGACAGAGAAT
         AB621332.1 - Morex   AACCTTGTTTTTTTTT-ACTACTGTACGTAAGGTGTAAGAATGGAGTGCTGACAGAGAAT
 AB621319.1 - Nishinohoshi   AACCTTGTTTTTTTTT-ACTACTGTACGTAAGGTGTAAGAATGGAGCGCTGACAGAGAAT

         AB621318.1 - TR251   GGATGCAGGTGCTGCTGAACCACCCGAGCCACCGCCGGCAGACGGGCCCGCCGGCGAGCG
     AB621312.1 - CDC Fibar   GGATGCAGGTGCTGCTGAACCACCCGAGCCACCGCCGGCAGACGGGCCCGCCGGCGAGCG
         AB621313.1 - Azhul   GGATGCAGGTGCTGCTGAACCACCCGAGCCACCGCCGGCAGACGGGCCCGCCGGCGAGCG
   AB621325.1 - B83-12/21/3   GGATGCAGGTGCTGCTGAACCACCCGAGCCACCGCCGGCAGACGGGCCCGCCGGCGAGCG
          AB621322.1 - Beka   GGATGCAGGTGCTGCTGAACCACCCGAGCCACCGCCGGCAGACGGGCCCGCCGGCGAGCG
      AB621321.1 - CDC Bold   GGATGCAGGTGCTGCTGAACCACCCGAGCCACCGCCGGCAGACGGGCCCGCCGGCGAGCG
         AB621332.1 - Morex   GGATGCAGGTGCTGCTGAACCACCCGAGCCACCGCCGGCAGACGGGCCCGCCGGCGAGCG
 AB621319.1 - Nishinohoshi   GGATGCAGGTGCTGCTGAACCACCCGAGCCACCGCCGGCAGACGGGCCCGCCGGCGAGCG

         AB621318.1 - TR251   CTGACAACCCACTGGACTTGAGCGGCGTGGATGTGCGTCTCCCCATGCTGGTGTACGTGT
     AB621312.1 - CDC Fibar   CTGACAACCCACTGGACTTGAGCGGCGTGGATGTGCGTCTCCCCATGCTGGTGTACGTGT
         AB621313.1 - Azhul   CTGACAACCCACTGGACTTGAGCGGCGTGGATGTGCGTCTCCCCATGCTGGTGTACGTGT
   AB621325.1 - B83-12/21/3   CTGACAACCCACTGGACTTGAGCGGCGTGGATGTGCGTCTCCCCATGCTGGTGTACGTGT
          AB621322.1 - Beka   CTGACAACCCACTGGACTTGAGCGGCGTGGATGTGCGTCTCCCCATGCTGGTGTACGTGT
      AB621321.1 - CDC Bold   CTGACAACCCACTGGACTTGAGCGGCGTGGATGTGCGTCTCCCCATGCTGGTGTACGTGT
         AB621332.1 - Morex   CTGACAACCCACTGGACTTGAGCGGCGTGGATGTGCGTCTCCCCATGCTGGTGTACGTGT
 AB621319.1 - Nishinohoshi   CTGACAACCCACTGGACTTGAGCGGCGTGGATGTGCGTCTCCCCATGCTGGTGTACGTGT

         AB621318.1 - TR251   CCCGTGAGAAGCGCCCCGGGCACGACCACCAGAAGAAGGCCGGTGCCATGAACGCGCTTA
     AB621312.1 - CDC Fibar   CCCGTGAGAAGCGCCCCGGGCACGACCACCAGAAGAAGGCCGGTGCCATGAACGCGCTTA
         AB621313.1 - Azhul   CCCGTGAGAAGCGCCCCGGGCACGACCACCAGAAGAAGGCCGGTGCCATGAACGCGCTTA
   AB621325.1 - B83-12/21/3   CCCGTGAGAAGCGCCCCGGGCACGACCACCAGAAGAAGGCCGGTGCCATGAACGCGCTTA
          AB621322.1 - Beka   CCCGTGAGAAGCGCCCCGGGCACGACCACCAGAAGAAGGCCGGTGCCATGAACGCGCTTA
      AB621321.1 - CDC Bold   CCCGTGAGAAGCGCCCCGGGCACGACCACCAGAAGAAGGCCGGTGCCATGAACGCGCTTA
         AB621332.1 - Morex   CCCGTGAGAAGCGCCCCGGGCACGACCACCAGAAGAAGGCCGGTGCCATGAACGCGCTTA
 AB621319.1 - Nishinohoshi   CCCGTGAGAAGCGCCCCGGGCACGACCACCAGAAGAAGGCCGGTGCCATGAACGCGCTTA

         AB621318.1 - TR251   CCCGCGCCTCGGCGCTGCTCTCCAACTCCCCCTTCATCCTCAACCTCGACTGCGATCATT
     AB621312.1 - CDC Fibar   CCCGCGCCTCGGCGCTGCTCTCCAACTCCCCCTTCATCCTCAACCTCGACTGCGATCATT
         AB621313.1 - Azhul   CCCGCGCCTCGGCGCTGCTCTCCAACTCCCCCTTCATCCTCAACCTCGACTGCGATCATT
   AB621325.1 - B83-12/21/3   CCCGCGCCTCGGCGCTGCTCTCCAACTCCCCCTTCATCCTCAACCTCGACTGCGATCATT
          AB621322.1 - Beka   CCCGCGCCTCGGCGCTGCTCTCCAACTCCCCCTTCATCCTCAACCTCGACTGCGATCATT
      AB621321.1 - CDC Bold   CCCGCGCCTCGGCGCTGCTCTCCAACTCCCCCTTCATCCTCAACCTCGACTGCGATCATT
         AB621332.1 - Morex   CCCGCGCCTCGGCGCTGCTCTCCAACTCCCCCTTCATCCTCAACCTCGACTGCGATCATT
 AB621319.1 - Nishinohoshi   CCCGCGCCTCGGCGCTGCTCTCCAACTCCCCCTTCATCCTCAACCTCGACTGCGATCATT

         AB621318.1 - TR251   ACATCAACAACTCCCAGGCCCTTCGCGCCGGCATCTGCTTCATGGTGGGACGGGACAGCG
     AB621312.1 - CDC Fibar   ACATCAACAACTCCCAGGCCCTTCGCGCCGGCATCTGCTTCATGGTGGGACGGGACAGCG
         AB621313.1 - Azhul   ACATCAACAACTCCCAGGCCCTTCGCGCCGGCATCTGCTTCATGGTGGGACGGGACAGCG
   AB621325.1 - B83-12/21/3   ACATCAACAACTCCCAGGCCCTTCGCGCCGGCATCTGCTTCATGGTGGGACGGGACAGCG
          AB621322.1 - Beka   ACATCAACAACTCCCAGGCCCTTCGCGCCGGCATCTGCTTCATGGTGGGACGGGACAGCG
      AB621321.1 - CDC Bold   ACATCAACAACTCCCAGGCCCTTCGCGCCGGCATCTGCTTCATGGTGGGACGGGACAGCG
         AB621332.1 - Morex   ACATCAACAACTCCCAGGCCCTTCGCGCCGGCATCTGCTTCATGGTGGGACGGGACAGCG
 AB621319.1 - Nishinohoshi   ACATCAACAACTCCCAGGCCCTTCGCGCCGGCATCTGCTTCATGGTGGGACGGGACAGCG

         AB621318.1 - TR251   ACACGGTTGCCTTCGTCCAGTTCCCGCAGCGCTTCGAGGGCGTCGACCCCACCGACCTCT
     AB621312.1 - CDC Fibar   ACACGGTTGCCTTCGTCCAGTTCCCGCAGCGCTTCGAGGGCGTCGACCCCACCGACCTCT
         AB621313.1 - Azhul   ACACGGTTGCCTTCGTCCAGTTCCCGCAGCGCTTCGAGGGCGTCGACCCCACCGACCTCT
   AB621325.1 - B83-12/21/3   ACACGGTTGCCTTCGTCCAGTTCCCGCAGCGCTTCGAGGGCGTCGACCCCACCGACCTCT
          AB621322.1 - Beka   ACACGGTTGCCTTCGTCCAGTTCCCGCAGCGCTTCGAGGGCGTCGACCCCACCGACCTCT
      AB621321.1 - CDC Bold   ACACGGTTGCCTTCGTCCAGTTCCCGCAGCGCTTCGAGGGCGTCGACCCCACCGACCTCT
         AB621332.1 - Morex   ACACGGTTGCCTTCGTCCAGTTCCCGCAGCGCTTCGAGGGCGTCGACCCCACCGACCTCT
 AB621319.1 - Nishinohoshi   ACACGGTTGCCTTCGTCCAGTTCCCGCAGCGCTTCGAGGGCGTCGACCCCACCGACCTCT

         AB621318.1 - TR251   ACGCCAACCACAACCGCATCTTCTTCGACGGCACCCTCCGTGCCCTGGACGGCATGCAGG
     AB621312.1 - CDC Fibar   ACGCCAACCACAACCGCATCTTCTTCGACGGCACCCTCCGTGCCCTGGACGGCATGCAGG
         AB621313.1 - Azhul   ACGCCAACCACAACCGCATCTTCTTCGACGGCACCCTCCGTGCCCTGGACGGCATGCAGG
   AB621325.1 - B83-12/21/3   ACGCCAACCACAACCGCATCTTCTTCGACGGCACCCTCCGTGCCCTGGACGGCATGCAGG
          AB621322.1 - Beka   ACGCCAACCACAACCGCATCTTCTTCGACGGCACCCTCCGTGCCCTGGACGGCATGCAGG
      AB621321.1 - CDC Bold   ACGCCAACCACAACCGCATCTTCTTCGACGGCACCCTCCGTGCCCTGGACGGCATGCAGG
         AB621332.1 - Morex   ACGCCAACCACAACCGCATCTTCTTCGACGGCACCCTCCGTGCCCTGGACGGCATGCAGG
 AB621319.1 - Nishinohoshi   ACGCCAACCACAACCGCATCTTCTTCGACGGCACCCTCCGTGCCCTGGACGGCATGCAGG
```

```
    AB621318.1 - TR251       GCCCCATCTACGTCGGCACTGGGTGTCTCTTCCGCCGCATCACCGTCTACGGCTTCGACC
AB621312.1 - CDC Fibar       GCCCCATCTACGTCGGCACTGGGTGTCTCTTCCGCCGCATCACCGTCTACGGCTTCGACC
    AB621313.1 - Azhul       GCCCCATCTACGTCGGCACTGGGTGTCTCTTCCGCCGCATCACCGTCTACGGCTTCGACC
AB621325.1 - B83-12/21/3     GCCCCATCTACGTCGGCACTGGGTGTCTCTTCCGCCGCATCACCGTCTACGGCTTCGACC
     AB621322.1 - Beka       GCCCCATCTACGTCGGCACTGGGTGTCTCTTCCGCCGCATCACCGTCTACGGCTTCGACC
 AB621321.1 - CDC Bold       GCCCCATCTACGTCGGCACTGGGTGTCTCTTCCGCCGCATCACCGTCTACGGCTTCGACC
    AB621332.1 - Morex       GCCCCATCTACGTCGGCACTGGGTGTCTCTTCCGCCGCATCACCGTCTACGGCTTCGACC
AB621319.1 - Nishinohoshi    GCCCCATCTACGTCGGCACTGGGTGTCTCTTCCGCCGCATCACCGTCTACGGCTTCGACC

    AB621318.1 - TR251       CGCCGAGGATCAACGTCGGCGGTCCCTGCTTCCCCAGGCTCGCCGGGCTCTTCGCCAAGA
AB621312.1 - CDC Fibar       CGCCGAGGATCAACGTCGGCGGTCCCTGCTTCCCCAGGCTCGCCGGGCTCTTCGCCAAGA
    AB621313.1 - Azhul       CGCCGAGGATCAACGTCGGCGGTCCCTGCTTCCCCAGGCTCGCCGGGCTCTTCGCCAAGA
AB621325.1 - B83-12/21/3     CGCCGAGGATCAACGTCGGCGGTCCCTGCTTCCCCAGGCTCGCCGGGCTCTTCGCCAAGA
     AB621322.1 - Beka       CGCCGAGGATCAACGTCGGCGGTCCCTGCTTCCCCAGGCTCGCCGGGCTCTTCGCCAAGA
 AB621321.1 - CDC Bold       CGCCGAGGATCAACGTCGGCGGTCCCTGCTTCCCCAGGCTCGCCGGGCTCTTCGCCAAGA
    AB621332.1 - Morex       CGCCGAGGATCAACGTCGGCGGTCCCTGCTTCCCCAGGCTCGCCGGGCTCTTCGCCAAGA
AB621319.1 - Nishinohoshi    CGCCGAGGATCAACGTCGGCGGTCCCTGCTTCCCCAGGCTCGCCGGGCTCTTCGCCAAGA

    AB621318.1 - TR251       CCAAGTACGAGAAGCCCGGGCTCGAGATGACCACGGCCAAGGCCAAGGCCGCGCCCGTGC
AB621312.1 - CDC Fibar       CCAAGTACGAGAAGCCCGGGCTCGAGATGACCACGGCCAAGGCCAAGGCCGCGCCCGTGC
    AB621313.1 - Azhul       CCAAGTACGAGAAGCCCGGGCTCGAGATGACCACGGCCAAGGCCAAGGCCGCGCCCGTGC
AB621325.1 - B83-12/21/3     CCAAGTACGAGAAGCCCGGGCTCGAGATGACCACGGCCAAGGCCAAGGCCGCGCCCGTGC
     AB621322.1 - Beka       CCAAGTACGAGAAGCCCGGGCTCGAGATGACCACGGCCAAGGCCAAGGCCGCGCCCGTGC
 AB621321.1 - CDC Bold       CCAAGTACGAGAAGCCCGGGCTCGAGATGACCACGGCCAAGGCCAAGGCCGCGCCCGTGC
    AB621332.1 - Morex       CCAAGTACGAGAAGCCCGGGCTCGAGATGACCACGGCCAAGGCCAAGGCCGCGCCCGTGC
AB621319.1 - Nishinohoshi    CCAAGTACGAGAAGCCCGGGCTCGAGATGACCACGGCCAAGGCCAAGGCCGCGCCCGTGC

    AB621318.1 - TR251       CCGCCAAGGGTAAGCACGGCTTCTTGCCACTGCCCAAGAAGACGTACGGCAAGTCGGACG
AB621312.1 - CDC Fibar       CCGCCAAGGGTAAGCACGGCTTCTTGCCACTGCCCAAGAAGACGTACGGCAAGTCGGACG
    AB621313.1 - Azhul       CCGCCAAGGGTAAGCACGGCTTCTTGCCACTGCCCAAGAAGACGTACGGCAAGTCGGACG
AB621325.1 - B83-12/21/3     CCGCCAAGGGTAAGCACGGCTTCTTGCCACTGCCCAAGAAGACGTACGGCAAGTCGGACG
     AB621322.1 - Beka       CCGCCAAGGGTAAGCACGGCTTCTTGCCACTGCCCAAGAAGACGTACGGCAAGTCGGACG
 AB621321.1 - CDC Bold       CCGCCAAGGGTAAGCACGGCTTCTTGCCACTGCCCAAGAAGACGTACGGCAAGTCGGACG
    AB621332.1 - Morex       CCGCCAAGGGTAAGCACGGCTTCTTGCCACTGCCCAAGAAGACGTACGGCAAGTCGGACG
AB621319.1 - Nishinohoshi    CCGCCAAGGGTAAGCACGGCTTCTTGCCACTGCCCAAGAAGACGTACGGCAAGTCGGACG

                                                                     SNP23
    AB621318.1 - TR251       CCTTCGTGGACACCATCCCGCGCGCGTCGCACCCGTCGCCCTACACCGCGGCGGCTGAGG
AB621312.1 - CDC Fibar       CCTTCGTGGACACCATCCCGCGCGCGTCGCACCCGTCGCCCTACGCCGCGGCGGCTGAGG
    AB621313.1 - Azhul       CCTTCGTGGACACCATCCCGCGCGCGTCGCACCCGTCGCCCTACGCCGCGGCGGCTGAGG
AB621325.1 - B83-12/21/3     CCTTCGTGGACACCATCCCGCGCGCGTCGCACCCGTCGCCCTACGCCGCGGCGGCTGAGG
     AB621322.1 - Beka       CCTTCGTGGACACCATCCCGCGCGCGTCGCACCCGTCGCCCTACGCCGCGGCGGCTGAGG
 AB621321.1 - CDC Bold       CCTTCGTGGACACCATCCCGCGCGCGTCGCACCCGTCGCCCTACGCCGCGGCGGCTGAGG
    AB621332.1 - Morex       CCTTCGTGGACACCATCCCGCGCGCGTCGCACCCGTCGCCCTACGCCGCGGCGGCTGAGG
AB621319.1 - Nishinohoshi    CCTTCGTGGACACCATCCCGCGCGCGTCGCACCCGTCGCCCTACGCCGCGGCGGCTGAGG

    AB621318.1 - TR251       GGATCGTGGCCGACGAGGCGACCATCGTCGAGGCGGTGAACGTGACGGCCGCCGCGTTCG
AB621312.1 - CDC Fibar       GGATCGTGGCCGACGAGGCGACCATCGTCGAGGCGGTGAACGTGACGGCCGCCGCGTTCG
    AB621313.1 - Azhul       GGATCGTGGCCGACGAGGCGACCATCGTCGAGGCGGTGAACGTGACGGCCGCCGCGTTCG
AB621325.1 - B83-12/21/3     GGATCGTGGCCGACGAGGCGACCATCGTCGAGGCGGTGAACGTGACGGCCGCCGCGTTCG
     AB621322.1 - Beka       GGATCGTGGCCGACGAGGCGACCATCGTCGAGGCGGTGAACGTGACGGCCGCCGCGTTCG
 AB621321.1 - CDC Bold       GGATCGTGGCCGACGAGGCGACCATCGTCGAGGCGGTGAACGTGACGGCCGCCGCGTTCG
    AB621332.1 - Morex       GGATCGTGGCCGACGAGGCGACCATCGTCGAGGCGGTGAACGTGACGGCCGCCGCGTTCG
AB621319.1 - Nishinohoshi    GGATCGTGGCCGACGAGGCGACCATCGTCGAGGCGGTGAACGTGACGGCCGCCGCGTTCG

    AB621318.1 - TR251       AGAAGAAGACCGGCTGGGGCAAAGAGATCGGCTGGGTGTACGACACCGTCACGGAGGACG
AB621312.1 - CDC Fibar       AGAAGAAGACCGGCTGGGGCAAAGAGATCGGCTGGGTGTACGACACCGTCACGGAGGACG
    AB621313.1 - Azhul       AGAAGAAGACCGGCTGGGGCAAAGAGATCGGCTGGGTGTACGACACCGTCACGGAGGACG
AB621325.1 - B83-12/21/3     AGAAGAAGACCGGCTGGGGCAAAGAGATCGGCTGGGTGTACGACACCGTCACGGAGGACG
     AB621322.1 - Beka       AGAAGAAGACCGGCTGGGGCAAAGAGATCGGCTGGGTGTACGACACCGTCACGGAGGACG
 AB621321.1 - CDC Bold       AGAAGAAGACCGGCTGGGGCAAAGAGATCGGCTGGGTGTACGACACCGTCACGGAGGACG
    AB621332.1 - Morex       AGAAGAAGACCGGCTGGGGCAAAGAGATCGGCTGGGTGTACGACACCGTCACGGAGGACG
AB621319.1 - Nishinohoshi    AGAAGAAGACCGGCTGGGGCAAAGAGATCGGCTGGGTGTACGACACCGTCACGGAGGACG

    AB621318.1 - TR251       TGGTCACCGGCTACCGGATGCATATCAAGGGGTGGCGGTCACGCTACTGCTCCATCTACC
AB621312.1 - CDC Fibar       TGGTCACCGGCTACCGGATGCATATCAAGGGGTGGCGGTCACGCTACTGCTCCATCTACC
    AB621313.1 - Azhul       TGGTCACCGGCTACCGGATGCATATCAAGGGGTGGCGGTCACGCTACTGCTCCATCTACC
AB621325.1 - B83-12/21/3     TGGTCACCGGCTACCGGATGCATATCAAGGGGTGGCGGTCACGCTACTGCTCCATCTACC
     AB621322.1 - Beka       TGGTCACCGGCTACCGGATGCATATCAAGGGGTGGCGGTCACGCTACTGCTCCATCTACC
 AB621321.1 - CDC Bold       TGGTCACCGGCTACCGGATGCATATCAAGGGGTGGCGGTCACGCTACTGCTCCATCTACC
    AB621332.1 - Morex       TGGTCACCGGCTACCGGATGCATATCAAGGGGTGGCGGTCACGCTACTGCTCCATCTACC
AB621319.1 - Nishinohoshi    TGGTCACCGGCTACCGGATGCATATCAAGGGGTGGCGGTCACGCTACTGCTCCATCTACC

    AB621318.1 - TR251       CACACGCCTTCATCGGCACCGCCCCCATCAACCTCACGGAGAGGCTCTTCCAGGTGCTCC
AB621312.1 - CDC Fibar       CACACGCCTTCATCGGCACCGCCCCCATCAACCTCACGGAGAGGCTCTTCCAGGTGCTCC
    AB621313.1 - Azhul       CACACGCCTTCATCGGCACCGCCCCCATCAACCTCACGGAGAGGCTCTTCCAGGTGCTCC
AB621325.1 - B83-12/21/3     CACACGCCTTCATCGGCACCGCCCCCATCAACCTCACGGAGAGGCTCTTCCAGGTGCTCC
     AB621322.1 - Beka       CACACGCCTTCATCGGCACCGCCCCCATCAACCTCACGGAGAGGCTCTTCCAGGTGCTCC
 AB621321.1 - CDC Bold       CACACGCCTTCATCGGCACCGCCCCCATCAACCTCACGGAGAGGCTCTTCCAGGTGCTCC
    AB621332.1 - Morex       CACACGCCTTCATCGGCACCGCCCCCATCAACCTCACGGAGAGGCTCTTCCAGGTGCTCC
AB621319.1 - Nishinohoshi    CACACGCCTTCATCGGCACCGCCCCCATCAACCTCACGGAGAGGCTCTTCCAGGTGCTCC
```

```
       AB621318.1 - TR251   GCTGGTCCACGGGATCCCTCGAGATCTTCTTCTCCAAGAACAACCCGCTCTTCGGCAGCA
   AB621312.1 - CDC Fibar   GCTGGTCCACGGGATCCCTCGAGATCTTCTTCTCCAAGAACAACCCGCTCTTCGGCAGCA
       AB621313.1 - Azhul   GCTGGTCCACGGGATCCCTCGAGATCTTCTTCTCCAAGAACAACCCGCTCTTCGGCAGCA
 AB621325.1 - B83-12/21/3   GCTGGTCCACGGGATCCCTCGAGATCTTCTTCTCCAAGAACAACCCGCTCTTCGGCAGCA
        AB621322.1 - Beka   GCTGGTCCACGGGATCCCTCGAGATCTTCTTCTCCAAGAACAACCCGCTCTTCGGCAGCA
    AB621321.1 - CDC Bold   GCTGGTCCACGGGATCCCTCGAGATCTTCTTCTCCAAGAACAACCCGCTCTTCGGCAGCA
       AB621332.1 - Morex   GCTGGTCCACGGGATCCCTCGAGATCTTCTTCTCCAAGAACAACCCGCTCTTCGGCAGCA
AB621319.1 - Nishinohoshi   GCTGGTCCACGGGATCCCTCGAGATCTTCTTCTCCAAGAACAACCCGCTCTTCGGCAGCA

       AB621318.1 - TR251   CATACCTCCACCCGCTGCAGCGCGTCGCCTACATCAACATCACCACTTACCCCTTCACCG
   AB621312.1 - CDC Fibar   CATACCTCCACCCGCTGCAGCGCGTCGCCTACATCAACATCACCACTTACCCCTTCACCG
       AB621313.1 - Azhul   CATACCTCCACCCGCTGCAGCGCGTCGCCTACATCAACATCACCACTTACCCCTTCACCG
 AB621325.1 - B83-12/21/3   CATACCTCCACCCGCTGCAGCGCGTCGCCTACATCAACATCACCACTTACCCCTTCACCG
        AB621322.1 - Beka   CATACCTCCACCCGCTGCAGCGCGTCGCCTACATCAACATCACCACTTACCCCTTCACCG
    AB621321.1 - CDC Bold   CATACCTCCACCCGCTGCAGCGCGTCGCCTACATCAACATCACCACTTACCCCTTCACCG
       AB621332.1 - Morex   CATACCTCCACCCGCTGCAGCGCGTCGCCTACATCAACATCACCACTTACCCCTTCACCG
AB621319.1 - Nishinohoshi   CATACCTCCACCCGCTGCAGCGCGTCGCCTACATCAACATCACCACTTACCCCTTCACCG

       AB621318.1 - TR251   CCATCTTCCTCATCTTCTACACCACCGTGCCGGCGCTATCCTTCGTCACCGGCCACTTCA
   AB621312.1 - CDC Fibar   CCATCTTCCTCATCTTCTACACCACCGTGCCGGCGCTATCCTTCGTCACCGGCCACTTCA
       AB621313.1 - Azhul   CCATCTTCCTCATCTTCTACACCACCGTGCCGGCGCTATCCTTCGTCACCGGCCACTTCA
 AB621325.1 - B83-12/21/3   CCATCTTCCTCATCTTCTACACCACCGTGCCGGCGCTATCCTTCGTCACCGGCCACTTCA
        AB621322.1 - Beka   CCATCTTCCTCATCTTCTACACCACCGTGCCGGCGCTATCCTTCGTCACCGGCCACTTCA
    AB621321.1 - CDC Bold   CCATCTTCCTCATCTTCTACACCACCGTGCCGGCGCTATCCTTCGTCACCGGCCACTTCA
       AB621332.1 - Morex   CCATCTTCCTCATCTTCTACACCACCGTGCCGGCGCTATCCTTCGTCACCGGCCACTTCA
AB621319.1 - Nishinohoshi   CCATCTTCCTCATCTTCTACACCACCGTGCCGGCGCTATCCTTCGTCACCGGCCACTTCA

       AB621318.1 - TR251   TCGTGCAGCGCCCGACCACCATGTTCTACGTCTACCTGGGCATCGTGCTATCCACGCTGC
   AB621312.1 - CDC Fibar   TCGTGCAGCGCCCGACCACCATGTTCTACGTCTACCTGGGCATCGTGCTATCCACGCTGC
       AB621313.1 - Azhul   TCGTGCAGCGCCCGACCACCATGTTCTACGTCTACCTGGGCATCGTGCTATCCACGCTGC
 AB621325.1 - B83-12/21/3   TCGTGCAGCGCCCGACCACCATGTTCTACGTCTACCTGGGCATCGTGCTATCCACGCTGC
        AB621322.1 - Beka   TCGTGCAGCGCCCGACCACCATGTTCTACGTCTACCTGGGCATCGTGCTATCCACGCTGC
    AB621321.1 - CDC Bold   TCGTGCAGCGCCCGACCACCATGTTCTACGTCTACCTGGGCATCGTGCTATCCACGCTGC
       AB621332.1 - Morex   TCGTGCAGCGCCCGACCACCATGTTCTACGTCTACCTGGGCATCGTGCTATCCACGCTGC
AB621319.1 - Nishinohoshi   TCGTGCAGCGCCCGACCACCATGTTCTACGTCTACCTGGGCATCGTGCTATCCACGCTGC

       AB621318.1 - TR251   TCGTCATCGCCGTGCTGGAGGTCAAGTGGGCCGGGGTCACAGTCTTCGAGTGGTTCAGGA
   AB621312.1 - CDC Fibar   TCGTCATCGCCGTGCTGGAGGTCAAGTGGGCCGGGGTCACAGTCTTCGAGTGGTTCAGGA
       AB621313.1 - Azhul   TCGTCATCGCCGTGCTGGAGGTCAAGTGGGCCGGGGTCACAGTCTTCGAGTGGTTCAGGA
 AB621325.1 - B83-12/21/3   TCGTCATCGCCGTGCTGGAGGTCAAGTGGGCCGGGGTCACAGTCTTCGAGTGGTTCAGGA
        AB621322.1 - Beka   TCGTCATCGCCGTGCTGGAGGTCAAGTGGGCCGGGGTCACAGTCTTCGAGTGGTTCAGGA
    AB621321.1 - CDC Bold   TCGTCATCGCCGTGCTGGAGGTCAAGTGGGCCGGGGTCACAGTCTTCGAGTGGTTCAGGA
       AB621332.1 - Morex   TCGTCATCGCCGTGCTGGAGGTCAAGTGGGCCGGGGTCACAGTCTTCGAGTGGTTCAGGA
AB621319.1 - Nishinohoshi   TCGTCATCGCCGTGCTGGAGGTCAAGTGGGCCGGGGTCACAGTCTTCGAGTGGTTCAGGA

       AB621318.1 - TR251   ACGGCCAGTTCTGGATGACAGCAAGTTGCTCCGCCTACCTCGCCGCCGTCTGCCAGGTGC
   AB621312.1 - CDC Fibar   ACGGCCAGTTCTGGATGACAGCAAGTTGCTCCGCCTACCTCGCCGCCGTCTGCCAGGTGC
       AB621313.1 - Azhul   ACGGCCAGTTCTGGATGACAGCAAGTTGCTCCGCCTACCTCGCCGCCGTCTGCCAGGTGC
 AB621325.1 - B83-12/21/3   ACGGCCAGTTCTGGATGACAGCAAGTTGCTCCGCCTACCTCGCCGCCGTCTGCCAGGTGC
        AB621322.1 - Beka   ACGGCCAGTTCTGGATGACAGCAAGTTGCTCCGCCTACCTCGCCGCCGTCTGCCAGGTGC
    AB621321.1 - CDC Bold   ACGGCCAGTTCTGGATGACAGCAAGTTGCTCCGCCTACCTCGCCGCCGTCTGCCAGGTGC
       AB621332.1 - Morex   ACGGCCAGTTCTGGATGACAGCAAGTTGCTCCGCCTACCTCGCCGCCGTCTGCCAGGTGC
AB621319.1 - Nishinohoshi   ACGGCCAGTTCTGGATGACAGCAAGTTGCTCCGCCTACCTCGCCGCCGTCTGCCAGGTGC

       AB621318.1 - TR251   TGACCAAGGTGATATTCCGGCGGGACATCTCCTTCAAGCTCACATCCAAGCTACCCTCGG
   AB621312.1 - CDC Fibar   TGACCAAGGTGATATTCCGGCGGGACATCTCCTTCAAGCTCACATCCAAGCTACCCTCGG
       AB621313.1 - Azhul   TGACCAAGGTGATATTCCGGCGGGACATCTCCTTCAAGCTCACATCCAAGCTACCCTCGG
 AB621325.1 - B83-12/21/3   TGACCAAGGTGATATTCCGGCGGGACATCTCCTTCAAGCTCACATCCAAGCTACCCTCGG
        AB621322.1 - Beka   TGACCAAGGTGATATTCCGGCGGGACATCTCCTTCAAGCTCACATCCAAGCTACCCTCGG
    AB621321.1 - CDC Bold   TGACCAAGGTGATATTCCGGCGGGACATCTCCTTCAAGCTCACATCCAAGCTACCCTCGG
       AB621332.1 - Morex   TGACCAAGGTGATATTCCGGCGGGACATCTCCTTCAAGCTCACATCCAAGCTACCCTCGG
AB621319.1 - Nishinohoshi   TGACCAAGGTGATATTCCGGCGGGACATCTCCTTCAAGCTCACATCCAAGCTACCCTCGG

       AB621318.1 - TR251   GAGACGAGAAGAAGGACCCCTACGCCGACCTCTACGTGGTGCGCTGGACGCCGCTCATGA
   AB621312.1 - CDC Fibar   GAGACGAGAAGAAGGACCCCTACGCCGACCTCTACGTGGTGCGCTGGACGCCGCTCATGA
       AB621313.1 - Azhul   GAGACGAGAAGAAGGACCCCTACGCCGACCTCTACGTGGTGCGCTGGACGCCGCTCATGA
 AB621325.1 - B83-12/21/3   GAGACGAGAAGAAGGACCCCTACGCCGACCTCTACGTGGTGCGCTGGACGCCGCTCATGA
        AB621322.1 - Beka   GAGACGAGAAGAAGGACCCCTACGCCGACCTCTACGTGGTGCGCTGGACGCCGCTCATGA
    AB621321.1 - CDC Bold   GAGACGAGAAGAAGGACCCCTACGCCGACCTCTACGTGGTGCGCTGGACGCCGCTCATGA
       AB621332.1 - Morex   GAGACGAGAAGAAGGACCCCTACGCCGACCTCTACGTGGTGCGCTGGACGCCGCTCATGA
AB621319.1 - Nishinohoshi   GAGACGAGAAGAAGGACCCCTACGCCGACCTCTACGTGGTGCGCTGGACGCCGCTCATGA

       AB621318.1 - TR251   TCACACCCATCATCATCATCTTCGTCAACATCATCGGATCCGCCGTGGCCTTCGCCAAGG
   AB621312.1 - CDC Fibar   TTACACCCATCATCATCATCTTCGTCAACATCATCGGATCCGCCGTGGCCTTCGCCAAGG
       AB621313.1 - Azhul   TTACACCCATCATCATCATCTTCGTCAACATCATCGGATCCGCCGTGGCCTTCGCCAAGG
 AB621325.1 - B83-12/21/3   TTACACCCATCATCATCATCTTCGTCAACATCATCGGATCCGCCGTGGCCTTCGCCAAGG
        AB621322.1 - Beka   TTACACCCATCATCATCATCTTCGTCAACATCATCGGATCCGCCGTGGCCTTCGCCAAGG
    AB621321.1 - CDC Bold   TTACACCCATCATCATCATCTTCGTCAACATCATCGGATCCGCCGTGGCCTTCGCCAAGG
       AB621332.1 - Morex   TTACACCCATCATCATCATCTTCGTCAACATCATCGGATCCGCCGTGGCCTTCGCCAAGG
AB621319.1 - Nishinohoshi   TTACACCCATCATCATCATCTTCGTCAACATCATCGGATCCGCCGTGGCCTTCGCCAAGG
```

```
        AB621318.1 - TR251   TTCTCGACGGCGAGTGGACGCACTGGCTCAAGGTCGCCGGCGGCGTCTTCTTCAACTTCT
   AB621312.1 - CDC Fibar   TTCTCGACGGCGAGTGGACGCACTGGCTCAAGGTCGCCGGCGGCGTCTTCTTCAACTTCT
        AB621313.1 - Azhul   TTCTCGACGGCGAGTGGACGCACTGGCTCAAGGTCGCCGGCGGCGTCTTCTTCAACTTCT
  AB621325.1 - B83-12/21/3   TTCTCGACGGCGAGTGGACGCACTGGCTCAAGGTCGCCGGCGGCGTCTTCTTCAACTTCT
         AB621322.1 - Beka   TTCTCGACGGCGAGTGGACGCACTGGCTCAAGGTCGCCGGCGGCGTCTTCTTCAACTTCT
    AB621321.1 - CDC Bold   TTCTCGACGGCGAGTGGACGCACTGGCTCAAGGTCGCCGGCGGCGTCTTCTTCAACTTCT
        AB621332.1 - Morex   TTCTCGACGGCGAGTGGACGCACTGGCTCAAGGTCGCCGGCGGCGTCTTCTTCAACTTCT
AB621319.1 - Nishinohoshi   TTCTCGACGGCGAGTGGACGCACTGGCTCAAGGTCGCCGGCGGCGTCTTCTTCAACTTCT

        AB621318.1 - TR251   GGGTGCTCTTCCACCTCTACCCCTTCGCCAAGGGCATCCTGGGGAAGCACGGAAAGACGC
   AB621312.1 - CDC Fibar   GGGTGCTCTTCCACCTCTACCCCTTCGCCAAGGGCATCCTGGGGAAGCACGGAAAGACGC
        AB621313.1 - Azhul   GGGTGCTCTTCCACCTCTACCCCTTCGCCAAGGGCATCCTGGGGAAGCACGGAAAGACGC
  AB621325.1 - B83-12/21/3   GGGTGCTCTTCCACCTCTACCCCTTCGCCAAGGGCATCCTGGGGAAGCACGGAAAGACGC
         AB621322.1 - Beka   GGGTGCTCTTCCACCTCTACCCCTTCGCCAAGGGCATCCTGGGGAAGCACGGAAAGACGC
    AB621321.1 - CDC Bold   GGGTGCTCTTCCACCTCTACCCCTTCGCCAAGGGCATCCTGGGGAAGCACGGAAAGACGC
        AB621332.1 - Morex   GGGTGCTCTTCCACCTCTACCCCTTCGCCAAGGGCATCCTGGGGAAGCACGGAAAGACGC
AB621319.1 - Nishinohoshi   GGGTGCTCTTCCACCTCTACCCCTTCGCCAAGGGCATCCTGGGGAAGCACGGAAAGACGC

        AB621318.1 - TR251   CAGTCGTGGTGCTCGTCTGGTGGGCATTCACCTTCGTCATCACCGCCGTGCTCTACATCA
   AB621312.1 - CDC Fibar   CAGTCGTGGTGCTCGTCTGGTGGGCATTCACCTTCGTCATCACCGCCGTGCTCTACATCA
        AB621313.1 - Azhul   CAGTCGTGGTGCTCGTCTGGTGGGCATTCACCTTCGTCATCACCGCCGTGCTCTACATCA
  AB621325.1 - B83-12/21/3   CAGTCGTGGTGCTCGTCTGGTGGGCATTCACCTTCGTCATCACCGCCGTGCTCTACATCA
         AB621322.1 - Beka   CAGTCGTGGTGCTCGTCTGGTGGGCATTCACCTTCGTCATCACCGCCGTGCTCTACATCA
    AB621321.1 - CDC Bold   CAGTCGTGGTGCTCGTCTGGTGGGCATTCACCTTCGTCATCACCGCCGTGCTCTACATCA
        AB621332.1 - Morex   CAGTCGTGGTGCTCGTCTGGTGGGCATTCACCTTCGTCATCACCGCCGTGCTCTACATCA
AB621319.1 - Nishinohoshi   CAGTCGTGGTGCTCGTCTGGTGGGCATTCACCTTCGTCATCACCGCCGTGCTCTACATCA

        AB621318.1 - TR251   ACATCCCCCACATGCATACCTCGGGAGGCAAGCACACAACGGTGCATGGTCACCATGGCA
   AB621312.1 - CDC Fibar   ACATCCCCCACATGCATACCTCGGGAGGCAAGCACACAACGGTGCATGGTCACCATGGCA
        AB621313.1 - Azhul   ACATCCCCCACATGCATACCTCGGGAGGCAAGCACACAACGGTGCATGGTCACCATGGCA
  AB621325.1 - B83-12/21/3   ACATCCCCCACATGCATACCTCGGGAGGCAAGCACACAACGGTGCATGGTCACCATGGCA
         AB621322.1 - Beka   ACATCCCCCACATGCATACCTCGGGAGGCAAGCACACAACGGTGCATGGTCACCATGGCA
    AB621321.1 - CDC Bold   ACATCCCCCACATGCATACCTCGGGAGGCAAGCACACAACGGTGCATGGTCACCATGGCA
        AB621332.1 - Morex   ACATCCCCCACATGCATACCTCGGGAGGCAAGCACACAACGGTGCATGGTCACCATGGCA
AB621319.1 - Nishinohoshi   ACATCCCCCACATGCATACCTCGGGAGGCAAGCACACAACGGTGCATGGTCACCATGGCA

        AB621318.1 - TR251   AAGTTGGTCGACACAGGGCTCTATGGCTGGCTCCATTGA
   AB621312.1 - CDC Fibar   AAGTTGGTCGACACAGGGCTCTATGGCTGGCTCCATTGA
        AB621313.1 - Azhul   AAGTTGGTCGACACAGGGCTCTATGGCTGGCTCCATTGA
  AB621325.1 - B83-12/21/3   AAGTTGGTCGACACAGGGCTCTATGGCTGGCTCCATTGA
         AB621322.1 - Beka   AAGTTGGTCGACACAGGGCTCTATGGCTGGCTCCATTGA
    AB621321.1 - CDC Bold   AAGTTGGTCGACACAGGGCTCTATGGCTGGCTCCATTGA
        AB621332.1 - Morex   AAGTTGGTCGACACAGGGCTCTATGGCTGGCTCCATTGA
AB621319.1 - Nishinohoshi   AAGTTGGTCGACACAGGGCTCTATGGCTGGCTCCATTGA
```

Fig. S5 Alignment of sequences for eight alleles of *HvCslF6*, showing the positions of polymorphisms (white text on black background) for which marker assays were developed. The sequence for Steptoe (AB621333.1) is identical to that of TR251. The sequences for Akashinriki (AB621314.1), Bowman (AB621309.1), Himalaya (AB621317.1), Logan (AB621323.1) and Shikoku Hadaka 84 (AB621320.1) are identical to that of CDC Bold. The sequences for Derkado (AB621324.1), Haruna Nijo (AB621310.1) and Sachiho Golden (AB621331.1) are identical to that of Beka. The sequence for Minerva (AB621305.1) is identical to that of B83-12/21/3
